# Networks of lexical borrowing and lateral gene transfer in language and genome evolution

**DOI:** 10.1002/bies.201300096

**Published:** 2013-12-27

**Authors:** Johann-Mattis List, Shijulal Nelson-Sathi, Hans Geisler, William Martin

**Affiliations:** 1 Research Center Deutscher Sprachatlas, Philipps-University MarburgMarburg, Germany; 2 Institute of Molecular Evolution, Heinrich-Heine University DüsseldorfDüsseldorf, Germany; 3 Institute of Romance Languages and Literature, Heinrich-Heine University DüsseldorfDüsseldorf, Germany

**Keywords:** borrowing, language evolution, lateral transfer, network approaches, prokaryotic evolution

## Abstract

Like biological species, languages change over time. As noted by Darwin, there are many parallels between language evolution and biological evolution. Insights into these parallels have also undergone change in the past 150 years. Just like genes, words change over time, and language evolution can be likened to genome evolution accordingly, but what kind of evolution? There are fundamental differences between eukaryotic and prokaryotic evolution. In the former, natural variation entails the gradual accumulation of minor mutations in alleles. In the latter, lateral gene transfer is an integral mechanism of natural variation. The study of language evolution using biological methods has attracted much interest of late, most approaches focusing on language tree construction. These approaches may underestimate the important role that borrowing plays in language evolution. Network approaches that were originally designed to study lateral gene transfer may provide more realistic insights into the complexities of language evolution.

## Introduction

For a long time, both biologists and linguists have been using family trees to model how species and languages evolve. But in contrast to biology – where the tree model is generally accepted to be the most realistic way to model how eukaryotic species (species with nucleated cells, such as animals and plants) evolve – linguists have always treated language trees with a certain suspicion. They have emphasized that – given the important role that horizontal transmission plays in language history – such trees can only capture vertical aspects of language evolution, while horizontal aspects (which linguists traditionally model as “waves” that spread out in circles around a center in geographic space) are ignored.

In the last decade, language trees have experienced a strong revival, especially in the public notion of linguistics as reflected in popular scientific literature and in articles addressed to a not exclusively linguistic readership 1. Earlier linguistic work on phylogenetic reconstruction was, with a few exceptions 2–8, qualitative in its nature. But starting about 10 years ago, computer methods originally designed to infer trees from molecular sequence data made their way into the analysis of large linguistic datasets, leading to a resurgence of language trees 9–15. If the reconstruction of trees had only played a minor role in historical linguistics up to that point, it has now become a specific field of interest, and some scholars even go so far as proclaiming tree construction as a priority for historical linguistic endeavor 16.

In traditional historical linguistics, these new approaches are met with a certain amount of reservation, since their results are often not in concordance with those achieved by traditional methods 17–20. One important reason for such discrepancies is the relatively large number of individual and methodological errors in linguistic datasets 19; this is reflected by numerous cases of wrong translations, wrong homology assessments (incorrect identification of cognate words), and undetected cases of lateral transfer (borrowing) 17,18.

In this paper, we argue that the problem of the new quantitative methods is that they focus too much on the vertical aspects of language evolution, thereby forcing the data into tree-like structures. We show that network approaches that were originally designed to study reticulation and lateral gene transfer in the evolution of prokaryotic species (microbes without cell nuclei, such as bacteria and archaea) can cope with these problems, hence providing a more realistic way to model the complexities of language history by combining both its tree-like (vertical) and its wave-like (horizontal) aspects.

## Historical linguists were always skeptical about language trees

**Figure 1:**
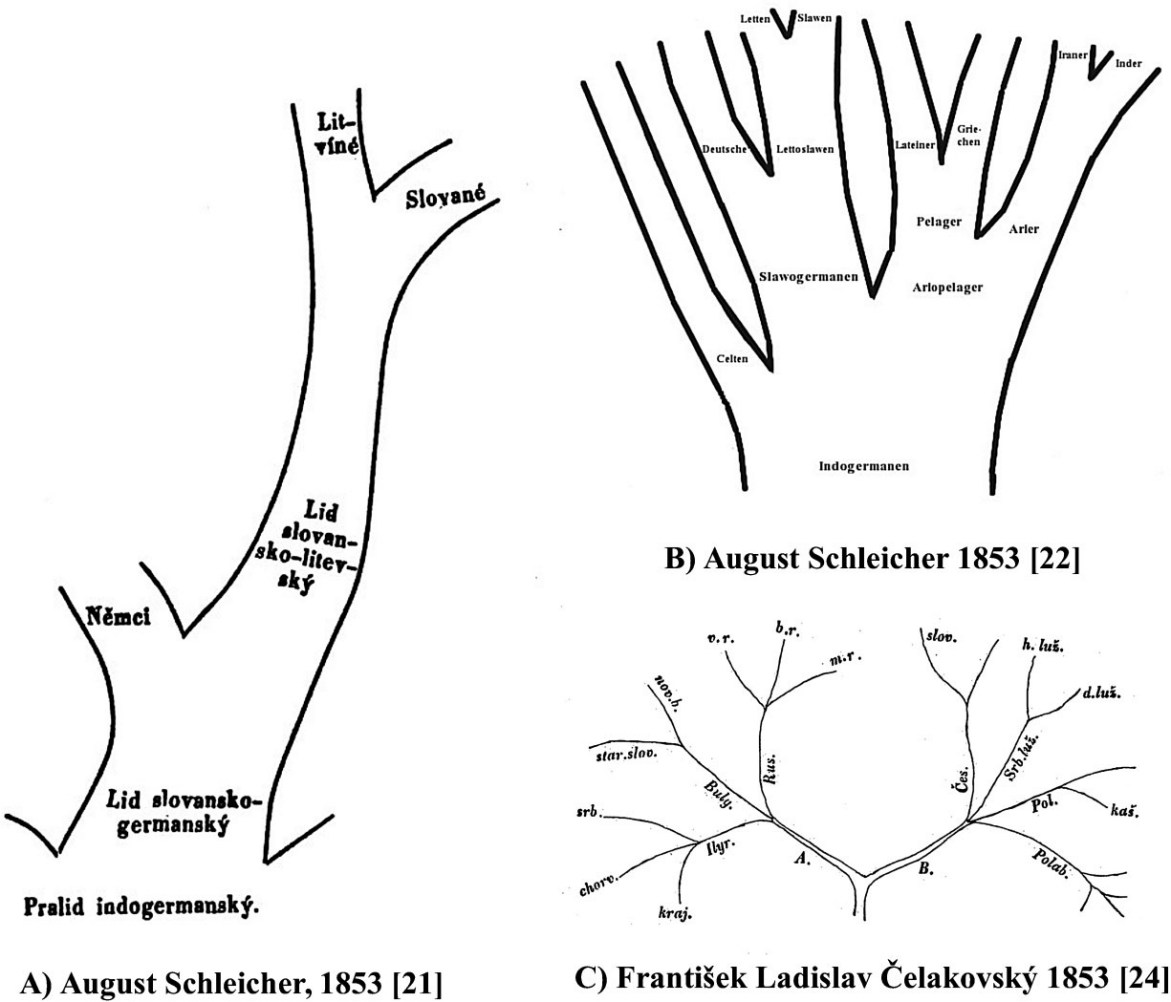
Three early language trees in the history of linguistics. A: August Schleicher's first tree of Germanic and Balto-Slavic languages. B: Schleicher's first tree of the Indo-European language family. C: An early tree of the Slavic languages by František Ladislav Čelakovský.

In 1853 the German linguist August Schleicher (1821–1868) published two articles 21,22 (Fig. 1A and B) in which he showed how branching trees can be used to illustrate the historical development of languages (Table 1A). It is possible 23 that Schleicher himself adopted the idea from a colleague, the Czech linguist František Ladislav Čelakovský (1759–1852), whose posthumously published lectures contain an early tree diagram of the Slavic languages 24 (Fig. 1C). Schleicher was very interested in biology, especially botany, and in his work we find many passages where he compares languages with organisms, assuming that they went through stages of birth, youth, middle age, old age, and – finally – death 25. He emphasized that language classification was quite similar to biological classification of animals or plants 25. He also mentioned the problem of distinguishing vertically from horizontally transmitted traits, drawing a parallel between “foreign influence” due to language contact in language history, and “crossbreeding” in evolutionary biology 26 (Table 1B).

**Table 1:** Early quotes on language history from August Schleicher and Hugo Schuchardt

(A) August Schleicher 26	
We know both the Old Latin and the Romance languages which demonstrably descended from the former via differentiation and – you would call it crossbreeding – foreign influence	*Wir kennen sowohl das Altlateinische, als auch die durch Differenzierung und durch fremden Einfluss – Ihr würdet sagen durch Kreuzung – nachweislich aus ihm hervorgegangenen romanischen Sprachen*
(B) August Schleicher 22	
These assumptions which logically follow from the previous research can be best illustrated with the help of a branching tree	*Diese Annahmen, logisch folgend aus den Ergebnissen der bisherigen Forschung, lassen sich am besten unter dem Bilde eines sich verästelnden Baumes anschaulich machen*
(C) Hugo Schuchardt 33	
We connect the branches and twigs of the family tree with countless horizontal lines and it ceases to be a tree	*Wir verbinden die Äste und Zweige des Stammbaums durch zahllose horizontale Linien, und er hört auf ein Stammbaum zu sein*

In biology, the concept of evolutionary trees was not introduced until Charles Darwin's (1809–1882) mentioning of the “Great Tree of Life” in 1859 27, but it soon became deeply ingrained in thinking on the topic. Notably, it was later reinforced by many influential drawings from Ernst Haeckel (1837–1919, see 28 for details), culminating in the inference of trees from molecular sequences 29, and the reconstruction of phylogenetic trees for all organisms using ribosomal and informational gene phylogenies 30.

In linguistics the popularity of language trees began to fade soon after it was first proposed 31. In 1872 Johannes Schmidt (1843–1901) pointed out that linguistic data contradicted the idea of simple, tree-like differentiation 32. Instead of the family tree theory he proposed the “wave theory” (*Wellentheorie* in German), which states that certain changes spread like waves in concentric circles over neighboring speech communities. And before Schmidt, Hugo Schuchardt (1842–1927) had criticized the idea of split and independent differentiation 33, emphasizing that languages diverge gradually while at the same time mutually influencing each other (Table 1C). Even today, historical linguists continue to hold strong reservations about the tree model. In text books on historical linguistics, both the tree and the wave theory are usually introduced as two complementary models, each of which only depicts one aspect of language history 34–35. Thus, if linguists are asked whether language evolves in a tree-like manner, most linguists would probably answer as Hoenigswald did in 1990: “Yes, of course it does, if we so wish; but we had better be very careful” 36.

## Borrowing is a constitutive part of language history

If we take the most frequent 1,000 Latin words and look at how they survived in its daughter languages, we will find that 67% of all words were directly inherited in at least one language, yet only 14% were inherited in all Romance languages 37. However, this drastic loss of Latin words during Romance language history is only part of the story: Since Latin never ceased to serve as a *cultural adstrate language* (a language that co-exists in some form in parallel with another language with which it is in contact), with a particularly great impact on written vernaculars, only 33% of all 1,000 words were completely lost, and about 50% survive as borrowings from the ancestor language in the daughter languages 37. Moreover, lexical transfer during the history of the Romance languages was not restricted to the influence of Latin alone, and contact among the Romance languages and other neighboring Indo-European languages was very frequent and vivid. According to a recent survey of 2,137 common words in Romanian 38, for example, 894 (41.8%) were classified as loanwords from other languages. The majority of these borrowed words were transferred from Slavic donor languages (about 14%). Only a small number of words were borrowed from Latin (about 3%).

On the “borrowability scale” 39, which ranks the ease with which different elements of language are assimilated by recipient languages, borrowing of *words* ranks highest. Lexical borrowing can affect only small parts of the vocabulary of a given language (such as specific terms for religious concepts, cultural items, or artifacts), or result in a situation where large parts of the language's original lexicon are replaced. This can even result in complete relexification, as in Creole languages. In the World Loanword Database 40 the frequency of direct borrowing events documented for 41 languages varies greatly, ranging from 1% for Mandarin Chinese to 62% for Selice Romani, with an average of 25% and a standard deviation of 13% 41.

## Borrowing cannot be ignored in quantitative approaches

With few exceptions 42,43, the majority of the new biological methods for tree construction makes use of lexical language data. This is due to the fact that it is much easier to compile lexical datasets for large numbers of languages: in many cases – especially for less-well studied language families – wordlists are the only things available for study. However, analysis of lexical items also reflects the basic practice of the traditional method for linguistic reconstruction, which starts with the comparison of words and morphemes 35,45. Similarly to earlier quantitative approaches in historical linguistics 8, the biological methods require that borrowings be filtered out of the data before the analysis is applied. Since reliable automatic methods are lacking, cognate and borrowing assignments are usually carried out manually. In order to make this painstaking process easier, scholars revived an old idea proposed in the 1950s 4,5, and restrict the lexical comparison to words that belong to the realm of the so-called “basic vocabulary” 12. Basic vocabulary is merely a technical term that refers to a list of about 100–200 basic concepts (such as “hand”, “foot”, “stone”) that are translated into the languages under investigation. These lists are usually called *Swadesh lists*, in acknowledgement of Morris Swadesh (1909–1967), who popularized their use in linguistics. The basic assumption regarding Swadesh lists is that (a) every language has words that express the concepts, (b) the words evolve slowly (enabling us to recognize similarities across languages), and (c) the words are rather resistant to borrowing 16. Unfortunately, the last assumption, in particular, is highly problematic. Although the use of Swadesh lists may decrease the number of borrowings to a certain degree, it cannot exclude all of them. In a recent survey of 1,504 common words in English, for example, 616 (41%) were judged to be loanwords 48, yet in the traditional English Swadesh list there are still 32 borrowings out of 200 (16.5%), mostly from Old Norse and Old French 18; and in a recent revision of the Albanian Swadesh list, 34 out of 107 words (31.8%) were identified as possible borrowings 49.

Manual detection of borrowings can range between trivial and impossible, depending on the case in point. Some borrowing processes are very transparent. Neither a linguist nor a German speaker has problems in identifying the word *Job* “job” as a recent borrowing from English, since the initial sound of the word is not yet “integrated” into the German sound system. But the situation is not always that simple. Thus, while no German native speaker would hesitate to assume that *Fett* “grease” is a “normal” German word, the word has in fact been borrowed from Low German dialects 50, as can be proven from its irregular correspondence with English *fat*: If the words were truly cognate, we would expect the German word to end with an [s] (spelled as *ß* in German) instead of a [t], as in German *heiß* “hot,” which is truly cognate with English *hot* 50. Identifying borrowings with help of these techniques requires expert knowledge of the languages under investigation, and the deeper one goes back in time, the harder it becomes even for the experts, since the available phonological information may be lost.

Recent tests on simulated data have shown how crucial it is to screen the linguistic data carefully before applying quantitative analyses 51. How difficult it is to prepare the data and to filter out all borrowings correctly is reflected by the fact that the most frequently used datasets, the *Comparative Indo-European Database* (52, http://www.wordgumbo.com/ie/cmp/), and the *Austronesian Basic Vocabulary Database* (53, http://language.psy.auckland.ac.nz/austronesian/), contain many undetected borrowings and various levels of erroneous cognate judgments 17–49. But “scrubbing” the data of false cognate assignments does not seem to be feasible for large datasets. Quantitative studies that are based on the *Indo-European Lexical Cognacy Database* (IELex, http://ielex.mpi.nl/), whose goal was to significantly enhance the notoriously flawed database composed by 52, still yield subgroupings that contradict traditional genetic classification (compare, for example, the strange grouping of Polish in 13 and 54). One reason for these problems is that the database still contains many undetected borrowings and other errors. The other reason is that the exclusion of borrowings necessarily yields a loss of information that can have large impacts on the results 49. It seems that the a priori exclusion of suspected borrowings from the data is not enough, especially in cases where the history of a language family is not yet well understood. Instead of making tree reconstruction the key objective of historical linguistics, we need quantitative methods that can deal with borrowings and – ideally – handle both vertical and lateral transmission.

## Language history bears a close resemblance to prokaryote evolution

If historical linguists want to profit from biological expertise in large-scale analyses of big datasets, they need to make up their mind regarding the methods they need in linguistics, and the methods that biology can provide. That evolutionary biology has developed some sophisticated tools to reconstruct phylogenetic trees, and that these tools can be easily applied to linguistic datasets, has been demonstrated frequently during the last decade. Yet is this really all that biology has to offer?

In several fundamental aspects, the genomes of eukaryotic species – such as animals and plants – and prokaryotic species – such as bacteria and archaea – evolve in very different ways, and lateral gene transfer is generally at the root of those differences. Gene families are one example. Gene families are sets of homologous (cognate) genes that were formed by duplication of an ancestral gene, quite similar to the reflexes of the root of a word in the same or different language. In eukaryotes, gene families arise through duplication: a resident gene duplicates, perhaps several times, and the resulting gene family consists of members that are closely related at the outset and undergo divergence and functional specialization 55. In prokaryotes, gene families arise via the acquisition of related sequences through lateral gene transfer, not through duplication 56. As another example, in eukaryotes, meiosis ensures that only members of the same species exchange genes, and recombination is reciprocal. In prokaryotes, there are well-studied mechanisms that mediate gene transfer, both within and across species boundaries 57.

Furthermore, if we sequence 61 human genomes, we will find – to all intents and purposes – the same collection of about 30,000 genes in each individual, with allelic variants at many loci, and the 46 chromosomes will almost always be colinear: the genes appearing at similar positions. If we sequence 61 genomes of *Escherichia coli*, a bacterium usually found in the intestines of warm-blooded species, we will find about 4,500 genes in each individual genome, but only about 1,000 genes that are present in all genomes. Summing up the different genes we find in all individuals, there are about 18,000 different genes distributed among them, and this count will further increase if we add more individual genomes to this calculation, hence yielding an ever growing pangenome of *Escherichia coli* 58. These examples underscore fundamental differences in the nature of the processes of evolutionary divergence in prokaryotic and eukaryotic populations: Eukaryotic populations generate tree-like structures of divergence over time 59, while genome evolution in prokaryotes generates both tree-like and net-like components of relatedness over time 60.

Recalling the scores on shared inherited words and borrowings we reported for the Romance languages earlier, it seems obvious that language history shows a much closer resemblance to prokaryotic evolution than to eukaryotic evolution. Thus, if one says that language history and genome evolution have a lot in common, it seems much more appropriate to emphasize that language evolution may resemble prokaryotic evolution much more than it resembles eukaryotic evolution. We do not claim to make a binary distinction here: As the amount of contact-induced change differs from language to language, so do the underlying evolutionary processes, and it is rather a continuum between strictly tree-like and strictly network-like evolution that we are dealing with. Nevertheless, if we want to employ quantitative methods from biology to supplement our research in historical linguistics, it could be much more fruitful to get away from focusing exclusively on those methods that yield simple family trees, and instead look for methods that were designed to handle lateral transfer.

## Network approaches offer new possibilities for quantitative analyses in language evolution

Despite the dissatisfaction of many historical linguists with both the tree and the wave model, there are – to our knowledge – only a few attempts to combine both approaches within a new framework 35,61; furthermore, unfortunately most of these proposals remain a mere visualization of the scholars' intuitions regarding the data, from which no further insights can be drawn. If one wants to include both the vertical and the horizontal aspects, it seems natural to turn to networks as a format to represent language history.

In evolutionary biology, different network approaches have been developed in order to study reticulation in biological datasets (see the overviews in 63 and 64). Among the most popular of these methods are those that produce unrooted networks (splits graphs) such as *split decomposition* 65 or *NeighborNet* 66. These methods enjoy some popularity in recent quantitative studies in historical linguistics, and have been applied to quite a few different datasets 67–71. In contrast to the popular quantitative methods for tree construction, such as *Neighbor-Joining* 72, or *Bayesian inference* 73, they are unbiased with respect to “tree-likeness”, and provide a direct visualization of the degree of conflict in a given dataset 74. They have proven to be a very useful tool for data exploration, and have even been used to measure reticulation directly from lexical distance matrices across the world's language families 75. The drawback of these methods is that they are distance-based, hence aggregating lexical information on the taxonomic level. The information on shared cognates in the underlying datasets is converted to distance scores, and the result is an unrooted network that only indicates whether there are conflicting signals in the data, but does not directly point to the cognate sets that are responsible for these conflicts.

A more realistic modeling of language history could be achieved by methods that automatically infer hidden borrowings in the data. While quite common in evolutionary biology 76–77, these methods are still in their infancy in historical linguistics. Two early approaches 70–78 are distance-based, and therefore do not allow the direct identification of the characters that conflict in the reference trees. The first character-based approach to this problem 79 uses maximum parsimony to determine the characters that conflict with an inferred family tree. Unfortunately, the method has only been tested on a very small dataset, and no further applications are known to us. An alternative proposal expands the notion of *perfect phylogenetic trees* 10 to the notion of *perfect phylogenetic networks* 80. The method yields direct statements as to which characters have been inferred as being borrowed in a given dataset. Unfortunately, the algorithm is very time-consuming, and it is thus not feasible to apply it to larger datasets 81.

## Ancestral genome sizes reveal the minimum amount of lateral transfer in microbial evolution

**Figure 2:**
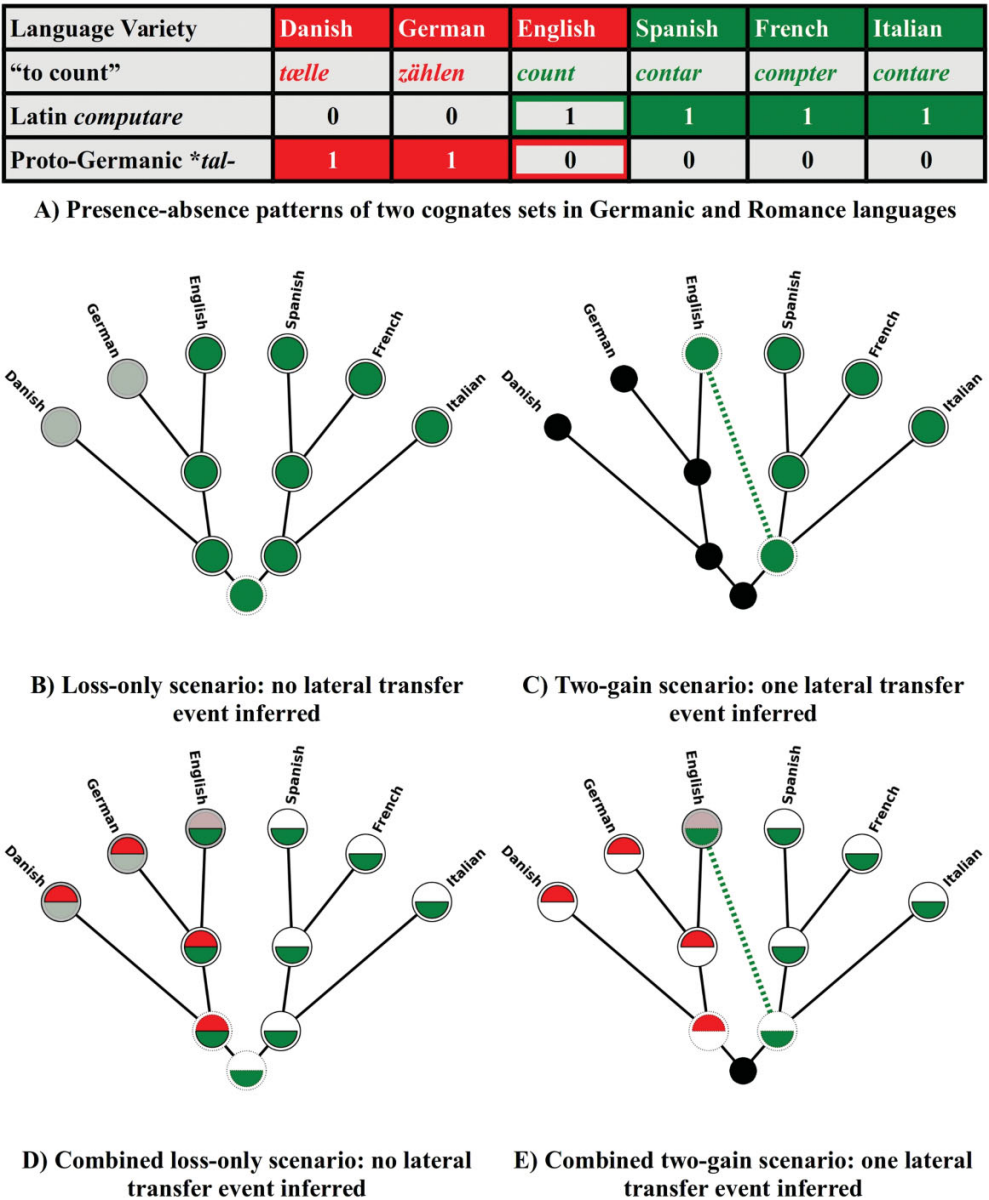
Illustration of the MLN method. A: Two cognate sets for “to count” in three Germanic and three Romance languages. The English word is a known borrowing from Old French. The original reflex of Proto-Germanic *tal- is still preserved in English “to tell,” but its original meaning has shifted under the influence of the borrowing from Old French, and it is thus not listed in this sample. B: The loss-only scenario assumes that the cognate set with reflexes of Latin originated in the root and was then lost independently in both German and Danish. C: The two-gain scenario infers two separate origins of the cognate sets. The pattern is thus suggestive of lateral transfer, and one lateral transfer event is inferred. This is marked by the link drawn between the two nodes where the characters first originate. D: Combination of scenarios for both cognate sets based on the loss-only scenario in B. Note that this scenario forces us to assume that the ancestor of the Germanic languages had two words expressing the concept “to count.” While this is not improbable per se, cases of inferred overwhelming amounts of synonymy are suspicious in language history. E: Combination of scenarios for both cognate sets based on the two-gain scenario in C. This scenario is preferred by the MLN method, since the number of synonyms in the ancestral languages is in balance with the modern languages. Note that the inference does not tell us which language is the real donor (which is Old French). According to our model, it could be any of the three Romance languages. For this reason, the edge is drawn between the ancestor off all languages.

A more recent method for lateral gene transfer detection in prokaryotic genomes is the so-called *minimal lateral network approach* (MLN, 82). This method applies the technique of gain-loss mapping 83,84 to presence-absence patterns of gene families in order to infer patterns that are suggestive of lateral transfer. Gain-loss mapping starts from a given reference tree that should reflect the vertical component of evolution as closely as possible. With help of the reference tree, specific gain-loss scenarios for all gene families in the dataset are inferred. A gain-loss scenario provides an explanation of how a given character could have evolved along the reference tree when character evolution is modeled as a simple process of gain and loss events. In order to confirm the assumption that a given character evolves in an exclusively vertical manner, the inferred gain-loss scenario should contain only one gain event. If more than one gain event is inferred, the character is judged to be suggestive of lateral transfer (see Fig. 2 for an example applied to linguistic data).

The crucial point of the MLN method is to select the best gain-loss scenarios out of the multitude of possible ones. The key argument in biology is the notion of ancestral genome size distributions 84: If, for example, all gene families are assumed to originate only once along the reference tree, this may result in ancestral genomes that contain much more genes than are observed in the contemporary genomes. If, on the other hand, one assumes that all gene families are explained by lateral gene transfer only, then the vertical component of genome evolution disappears, and ancestral genome sizes become too tiny to support life. Between those extremes there are amounts of vertical and lateral inheritance that will bring the distribution of inferred ancestral genome sizes into agreement with the attested distribution of contemporary genome sizes. Those distributions can be tested statistically, and the gain-loss scenarios with the amount of lateral gene transfer that best fits the data can be determined. Having selected the best scenarios, a rooted phylogenetic network can be reconstructed. Here, multiple origins of the same gene family on different branches of the reference tree are connected by lateral links; edges connecting the same two nodes for different gene families are joined to form weighted edges 82.

## How minimal lateral networks can be applied to linguistic data

Technically, the application of the MLN approach to language data can be carried out in a rather straightforward way, by investigating presence-absence patterns of cognate sets instead of presence-absence patterns in gene families. Theoretically, however, the application of the approach requires some caveats: while genomes are physical entities whose size can be directly determined, the linguistic data consist of samples based on meaning lists. We can restate the genome size criterion for scenario selection in such a way that we prefer those scenarios in which the number of words used to express specific meanings does not differ much between ancestral and contemporary languages. However, we need to keep in mind that new words can also shift into the meaning slots from outside the sample. Although parallel semantic shift involving cognate words in different branches of a language family is surely much rarer than borrowing, this has to be considered when applying the method to linguistic data.

The MLN approach was first applied to the well-known Comparative Indo-European Database 52, and revealed a rather high degree of non-tree-like signal: 61% of all 2,346 cognate sets in the data were found to be suggestive of borrowing 86. Since the study employed a very simple top-down algorithm for gain-loss mapping 84, the inferred amount of cognate sets contradicting the reference tree is surely too high. In order to test whether more refined techniques of gain-loss mapping can yield more realistic results, we applied a refined variant of the MLN approach to a subset of 40 Indo-European languages taken from the IELex (dump from May 2013 kindly provided by M. Dunn). The modified MLN approach is implemented as part of a freely available Python library for quantitative tasks in historical linguistics 87. It employs weighted parsimony for the task of gain-loss mapping 83 and also allows for a certain proportion of parallel evolution. A Python script along with the data to run all analyses can be downloaded from: https://gist.github.com/LinguList/7475830. The advantage of the IELex is that known borrowings are not only marked as such, but that they are also assigned to the cognate sets to which they would belong, if they were not borrowings. Thus, English *mountain* is clustered with the reflexes of Vulgar Latin *montanea (derived from Latin *mōns*) in the Romance languages, such as, among others, French *montagne*, Italian *montagna*, and Spanish *montaña*. This gives us the possibility to test the usefulness of the refined MLN approach. We corrected some obvious errors in the data, especially in some of the Slavic languages (the whole dataset is provided in Supplementary Material I). Excluding 1,864 words that could not be shown to be cognate to any other word in the data, this yielded a total of 1,190 cognate sets. As a reference tree, we chose the one provided by Ethnologue 88. The choice of this tree is for practical reasons, since it was proposed independently of quantitative methods, and reflects an openly available “quasi-standard”. This does not mean that we are unaware of the many problems that this tree contains, especially in the classification of the subgroups.

**Figure 3:**
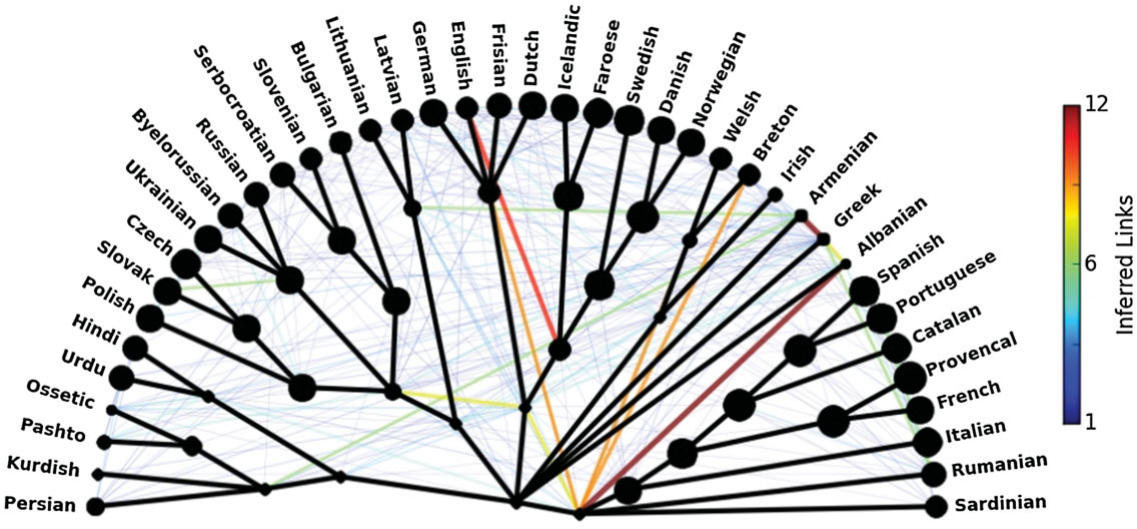
Minimal Lateral Network of 40 Indo-European languages. The size of the nodes reflects the number of cognate sets in each language as inferred by the MLN approach. The links reflect the minimal amount of lateral transfer events that is needed to bring the distributions of synonyms in the contemporary languages (leaves of the tree) and the ancestral languages (internal nodes of the tree) as closely together as possible.

Figure 3 shows the rooted phylogenetic network that the refined MLN approach reconstructed from the data. As can be seen, the method nicely recovers some well-known cases of contact relations among the languages in the sample. English, for example shows two heavily weighted edges, one with the ancestor of the Scandinavian languages, and one with the ancestor of the Romance languages, nicely reflecting two of its major donors: Scandinavian words made their way into the English lexicon as a result of Danish and northern Scandinavian invasions starting in the 8th–9th century 89, and Old Norman (a northern French dialect) came to England as a result of the Norman conquest in 1066. Old Norman even developed into a distinct variety called Anglo-Norman which was spoken in England by the higher social strata from 12th to 15th century. The ensuing intensive language contact results in a boom of “French” loans, which eventually became a formative element of the English lexicon 89. Albanian shows also strong connections with the ancestor of the Romance languages, reflecting the large number of Latin loanwords in the language 49.

Of the 105 cognate sets in the data that contain known hidden borrowings, the method identifies 76 correctly (see the specific results in Supplementary Material I). In total, the method identifies 369 out of 1,190 cognate sets (31%) that do not correspond to the reference tree. If the number of known borrowings reflected the true amount of borrowings in the data, and the reference tree displayed the true vertical history of the languages, this would mean that the method largely overstates the amount of lateral transfer. However, given the uncertainty regarding the subgrouping of the Indo-European languages that is also reflected in the reference tree, and the uncertainty of the cognate judgments in the data, we are confident that the results provide a good starting point for further research that may reveal further hidden borrowings and erroneous cognate judgments.

This can be exemplified by an inspection of the specific results that the method yields for English: Of the 32 borrowings into English 18, eight are singletons and five have reflexes in almost all Germanic languages in the sample and can thus technically not be identified by the MLN approach. Of the remaining 19 words, 17 (89%) are correctly identified. 17 further words are found to be not compatible with the reference tree, but three of these words are known borrowings in other languages. Of the remaining 14 words, four words (*belly*, *narrow*, *dull*, *smoke*), are obviously erroneously coded, since they are linked with words outside the Germanic branch, although their deeper etymology or the etymology of their presumed cognates is unclear; and four words (*at*, *leaf*, *small*, *know*) seem to be real cases of parallel semantic development (be it retention or innovation) with other languages (see Supplementary Material II). The remaining six words (*back*, *few*, *many*, *snake*, *tree*, *with*) are exclusively shared with the Scandinavian languages inside the Germanic branch. Whether this pattern results from innovations on the West Germanic mainland, by which the reflexes of the words in Frisian, German, and Dutch were replaced, or from hitherto unnoticed Scandinavian influence requires further investigation. A full list of all words with further comments is supplied in Supplementary Material II.

The modified MLN approach is surely not perfect. It heavily relies on the underlying data, and especially the selection of the reference tree can have a strong influence on the results. Furthermore, it can only recover those cases of borrowing that occur inside a given language family. External influences cannot be recovered. Further research is required in order to assess to which degree it overestimates borrowing rates because of its incapacity of handling independent parallel developments. However, it is a first step en route to more realistic quantitative models of language evolution, and could prove useful for scholars working on quantitative applications in historical linguistics, since it not only tests the tree-likeness of datasets but also provides direct hints as to the characters that cause reticulation. It can help us to improve the quality of our datasets by identifying possible hidden borrowings and erroneous cognate assignments.

## Conclusion and outlook

Different metaphors and models have, over the past century or two, been developed to describe the evolution of languages, but realistic quantitative models that can explain horizontal evolutionary processes in addition to genealogical relationships were lacking. Since similar evolutionary processes shaped both genomes and languages into contemporary forms, it is possible to apply methods that are developed to study genome evolution to study language evolution. Since lateral transfer in language evolution constitutes a real form of natural variation, phylogenetic network approaches provide a better means to model language evolution than strictly bifurcating phylogenetic trees. We strongly support the recent attempts to strengthen the quantitative basis of historical linguistics by building large databases and adapting computational methods from biology. Great work has been done in the past 10 years, and we know that errors are unavoidable when building large databases that accumulate historical linguistic knowledge. However, since errors are not only unavoidable, but – in the case of undetected borrowings – also reflect one vivid aspect of language history, we think it is time to rethink claims about the major processes underlying language evolution. Applying network approaches in historical linguistics can provide new insights into both the vertical and the lateral components of language history, and help to bring traditional and more quantitative research closer together.

## Supporting Information

As a service to our authors and readers, this journal provides supporting information supplied by the authors. Such materials are peer reviewed and may be re-organized for online delivery, but are not copy-edited or typeset. Technical support issues arising from supporting information (other than missing files) should be addressed to the authors.
