# Supplementary material for: Networks of lexical borrowing and lateral gene transfer in language and genome evolution
Source: Bioessays. 2013 Dec 27;36(2):141–50. doi: 10.1002/bies.201300096 (PMC3910147; doi:10.1002/bies.201300096)
Supplement: Supplementary file 1 [file bies0036-0141-sd1.docx]

Supplemental Material I: The dataset and the results of the analysis

**A) Notes on the Coding of the Results and the Data**

In the following, we list all the data that was considered for our study. In order to avoid that the data keeps some reasonable size, we don't list the 1864 “singletons”, i.e. words that were excluded from the analysis since they could not be shown to be cognate with any other word. We gladly share all the data including the singletons upon request. We would like to express our deep gratitude to Michael Dunn (MPI Nijmwegen) for sharing the data of the IELex with us (dump from May 2013).

The results in this analysis are given on a cognate-set basis. The first heading displays the “basic concept”. Followed by this, we list the cognate sets. Each original cognate set is given a specific ID that was mainly used for computational reasons. Since we were using the data for further tests on phonetic alignment, we give the phonetic transcriptions (where provided in the original data) instead of the orthographical representation of the words.

The results of the analysis are displayed as follows:

- Known borrowings (as marked in the source) are marked by underlining the respective entry.
- Cognate sets suggestive of borrowing are split into those parts that can be explained with help of the reference tree. This subgrouping of the cognate sets into different subsets is marked with a color code. Those cognate sets that have the same color are all judged to represent tree-like evolution by the MLN method. If more than one color is found in a cognate set, the cognate set is generally judged to be *patchy* (suggestive of borrowing, erroneous coding).
- The cognate sets where the MLN method correctly identifies a patchy pattern, are marked with an asterisk.
- The cognate sets where the MLN method fails to detect a patchy pattern are marked with two asterisks. Note that we included those cognate sets where borrowing or cognacy is pervasive: If all words in a given cognate set are known borrowings from an unknown source, the MLN method cannot detect such a pattern. Similarly, if a borrowed word occurs among cognates that are reflected throughout a whole subbranch, it won't be able to find these cases. We did not adjust for these cases here.

**B) The Data**

- - 1. Basic Concept: "I"

**Cognate Set "2720":** Albanian_Standard [unë], Armenian_Mod [jɛs], Breton_List [ME], Bulgarian [as], Byelorussian [JA], Catalan [ʒɔ], Czech [jaː], Danish [jɑj], Digor_Ossetic [ɐz], Dutch_List [ɪk], English [aɪ], Faroese [e:], French [ʒə], Frisian [ɪk], German [ɪç], Greek_Mod [e̞ˈɣo̞], Hindi [mɛ̃], Icelandic_ST [jɛɣ], Irish_A [ME], Italian [ˈio], Kurdish [ez], Kurdish [min], Latvian [es], Lithuanian_ST [ɐʃ], Norwegian [jæɪ], Pashto [ZE], Persian [mæn], Polish [ja], Portuguese_ST [eu], Provencal [jew], Rumanian_List [jew], Russian [ja], Sardinian_C [DEU], Serbocroatian [JA], Slovak [JA], Slovenian [JEST], Spanish [ʝo], Swedish [ˈjɑːɡ], Ukrainian [JA], Urdu [mɛ̃]

- - 1. Basic Concept: "all"

**Cognate Set "94":** Bulgarian [ˈfsit͡ʃki], Byelorussian [WSE], Czech [fʃɪxɲɪ], Latvian [visi], Lithuanian_ST [‘ʋʲɪskɐs], Polish [ˈfʃɨsʦɨ], Russian [vsʲo], Serbocroatian [SAV], Slovak [VSETKO], Slovenian [VSE], Ukrainian [UVESˈ]

**Cognate Set "815":** Kurdish [hemû], Persian [hæme]

**Cognate Set "677":** Danish [ɛl], Dutch_List [ˈɑləs], English [ɔ:l], Faroese [ALLIR], Frisian [ɔlə], German [ˈalə], Icelandic_ST [ALLIR], Norwegian [alə], Swedish [ˈala]

**Cognate Set "3019":** Greek_Mod [ˈo̞lo̞s], Breton_List [AN_HOLL], Irish_A [UILE]

**Cognate Set "2818":** Hindi [SEB], Urdu [səb]

**Cognate Set "2650":** Catalan [tot], French [tu], Italian [ˈtutto], Portuguese_ST [ˈtoðu], Provencal [TOUT], Rumanian_List [TOTI_(M._PL.)], Sardinian_C [TOTTU], Spanish [ˈtoðo]

- - 1. Basic Concept: "and"

**Cognate Set "95":** Bulgarian [i], Byelorussian [I], Czech [a], Polish [i], Russian [i], Serbocroatian [I], Slovak [A], Ukrainian [I]

**Cognate Set "835":** Hindi [OR], Urdu [ʔɔɾ]

**Cognate Set "744":** Rumanian_List [IAR], Lithuanian_ST [ir̃]

**Cognate Set "685":** Dutch_List [ɛn], English [ænd], Frisian [ɛn], German [ʊnt]

**Cognate Set "306":** Danish [ɒw], Faroese [oː], Icelandic_ST [ɔːɣ], Norwegian [OG], Swedish [ɔ(kː)]

**Cognate Set "3020":** Breton_List [HA], Irish_A [AGUS], Welsh_N [a:]

**Cognate Set "2899":** Catalan [i], French [e], Italian [e], Portuguese_ST [i], Provencal [E], Sardinian_C [E], Spanish [i]

**Cognate Set "2768":** Pashto [AU], Persian [VA]

- - 1. Basic Concept: "animal"

**Cognate Set "972":** Bulgarian [ʒiˈvɔtno], Latvian [dzîvnieks], Lithuanian_ST [GYVULYS], Russian [ʒɨˈvotnoe], Serbocroatian [ZIVOTINJA], Slovenian [ZIVALI], Irish_A [BEITHIDHEACH], Greek_Mod [ˈzo̞ˌo̞]

**Cognate Set "96":** Byelorussian [ZYVELA], Czech [zviːr̝ɛ], Latvian [zvērs], Lithuanian_ST [ZVERIS], Polish [ˈzvʲɛʒɛ̃], Slovak [ZVER]

**Cognate Set "816":** Pashto [HAJVAN], Persian [HEYVAN]

**Cognate Set "692":** Danish [ˈd̥yːʌ], Dutch_List [dir], Faroese [dʊir], Faroese [dʒɔur], Frisian [di.ər], German [ti:ɐ̯], Icelandic_ST [tiːr], Norwegian [dyːɾ], Swedish [jʉːr]

**Cognate Set "2769":** Hindi [ˈdʒanʋər], Persian [dʒɒːnˈvæɾ], Urdu [ʤɑnwəɾ]

***Cognate Set "2900":** English [ˈænɪməl], Breton_List [ANEVAL], Irish_A [AINMHE], Welsh_N [ANIFAIL], Catalan [əni’mal], French [animal], Italian [aniˈmale], Portuguese_ST [ɐniˈmaɫ], Provencal [ANIMAU], Rumanian_List [animal], Sardinian_C [ANIMALI], Spanish [aniˈmal]

- - 1. Basic Concept: "ashes"

**Cognate Set "97":** Bulgarian [ˈpɛpɛl], Byelorussian [POPEL], Czech [popɛl], Latvian [pelni], Lithuanian_ST [PELENAI], Polish [ˈpɔpʲuw], Serbocroatian [PEPEO], Slovak [POPOL], Slovenian [PEPJU], Ukrainian [POPIL]

**Cognate Set "693":** Danish [ˈasg̥ʰə], Dutch_List [ɑs], English [æʃɪz], Faroese [øska], Frisian [ˈjɪskə], German [ˈaʃə], Icelandic_ST [ˈaska], Norwegian [aske], Swedish [ˈaska]

**Cognate Set "548":** Hindi [rakʰ], Urdu [ɾɑkʰ]

**Cognate Set "426":** Greek_Mod [ˈstaxti], Swedish [stɔft]

**Cognate Set "3021":** Breton_List [LUDU], Irish_A [LUAITH], Welsh_N [LLUDW]

**Cognate Set "2902":** Albanian_Standard [hiri], Catalan [ˈsɛndrə], French [sɑ̃dʀ], Italian [ˈtʃenere], Portuguese_ST [ˈsĩzɐʃ], Provencal [CENDRE], Rumanian_List [cenușă], Sardinian_C [CINIZU], Spanish [θeˈniθas]

- - 1. Basic Concept: "at"

**Cognate Set "2141":** Digor_Ossetic [cor], Pashto [DE...SERA]

**Cognate Set "1824":** Dutch_List [tə]

**Cognate Set "1823":** Danish [veð], Faroese [VID], Icelandic_ST [VIO], Norwegian [VED], Swedish [viːd]

**Cognate Set "1820":** Irish_A [AIG], English [æt], Faroese [A], Catalan [a], French [a], Italian [a], Provencal [A], Rumanian_List [la], Sardinian_C [A], Spanish [a]

**Cognate Set "1819":** Bulgarian [na], Czech [NA], Slovenian [NE], Ukrainian [NA]

**Cognate Set "1818":** Czech [ʊ], Russian [u]

**Cognate Set "1816":** Czech [KU], Serbocroatian [KOD], Slovenian [K]

**Cognate Set "1815":** Byelorussian [PRY], Czech [pr̝̊ɪ], Latvian [pìe], Lithuanian_ST [PRIE], Lithuanian_ST [pɐs], Polish [pʃɨ], Slovak [PRI], Ukrainian [PRY]

**Cognate Set "1814":** Frisian [op], Hindi [-PER], Urdu [pəɾ]

**Cognate Set "1813":** Portuguese_ST [ɐ̃j], Breton_List [E], Welsh_N [YN], Polish [v], Russian [v], Ukrainian [V]

- - 1. Basic Concept: "back"

**Cognate Set "975":** Rumanian_List [spate], Catalan [əsˈpaʎʎə], Spanish [esˈpalda]

**Cognate Set "974":** Dutch_List [rʏχ], Faroese [RYGGUR], Frisian [rɛːx], German [ˈrijkn̩], Norwegian [RYGG], Swedish [rʏg]

**Cognate Set "706":** Latvian [mugura], Lithuanian_ST [NUGARA]

**Cognate Set "696":** English [bæk], Danish [b̥ɛj], Faroese [BAK], Icelandic_ST [paːk]

**Cognate Set "3022":** Breton_List [KEIN], Welsh_N [CEFN]

**Cognate Set "2903":** French [do], Italian [ˈdɔsso], Portuguese_ST [dorso], Provencal [DOS], Rumanian_List [DOS], Spanish [DORSO]

**Cognate Set "2770":** Hindi [piːʈʰ], Persian [POSHT]

**Cognate Set "1829":** Catalan [əsˈkɛnə], Provencal [ESQUINO]

***Cognate Set "889":** Albanian_Standard [shpinë], Russian [spiˈna], Ukrainian [SPYNA]

- - 1. Basic Concept: "bad"

**Cognate Set "982":** Italian [katˈtivo], Provencal [caitiu]

**Cognate Set "979":** Faroese [ILLUR], Icelandic_ST [ILLUR], Icelandic_ST [LELEGUR]

**Cognate Set "978":** Danish [ɔn], Icelandic_ST [VONDUR], Swedish [und]

**Cognate Set "871":** Norwegian [DARLIG], Swedish [ˈdoː.lɪ(ɡ)]

**Cognate Set "746":** Lithuanian_ST [‘blo:ɡɐs], Byelorussian [BLAGI]

**Cognate Set "429":** Greek_Mod [kaˈko̞s], Albanian_Standard [i_keq]

**Cognate Set "2904":** Catalan [mal], French [mɔvɛ], Portuguese_ST [maw], Sardinian_C [MALU], Spanish [ˈmalo]

**Cognate Set "2619":** Hindi [KHERAB], Urdu [χəɾɑb]

**Cognate Set "200":** Serbocroatian [ZAO], Czech [zliː], Polish [zwɨ], Slovak [ZLY]

**Cognate Set "1830":** Hindi [BURA], Urdu [bʊɾɑ]

***Cognate Set "977":** Armenian_Mod [vɑt], Pashto [BAD], Persian [BAD]

***Cognate Set "698":** Dutch_List [slɛχt], German [ʃlɛçt], Latvian [slikts]

- - 1. Basic Concept: "bark"

**Cognate Set "700":** Dutch_List [sχɔrs], Catalan [əsˈkɔrsə], French [ekɔʀs], Italian [korˈtettʃa], Italian [ˈskɔrdza], Portuguese_ST [CORTICA], Portuguese_ST [ˈkaʃkɐ], Rumanian_List [scoarță], Sardinian_C [KROZU], Spanish [koɾˈteθa], Bulgarian [kʊˈra], Byelorussian [KARA], Czech [kuːra], Polish [ˈkɔra], Russian [koˈra], Serbocroatian [KORA], Slovak [KORA]

**Cognate Set "2820":** Hindi [cʰal], Urdu [ʧɑl]

**Cognate Set "2689":** Pashto [POST], Persian [PUST]

***Cognate Set "308":** English [bɑ:k], Danish [b̥ɑːg̥ʰ], Faroese [ˈbœʃkʊr], Icelandic_ST [BORKUR], Norwegian [bark], Swedish [bark]

***Cognate Set "3025":** Provencal [RUSCO], Breton_List [RUSK], Welsh_N [RHISGL]

- - 1. Basic Concept: "because"

**Cognate Set "99":** Bulgarian [zɐˈʃtɔto], Byelorussian [TAGO_STO], Catalan [pərˈkɛ], French [paʀs(ə)_k(ə)], Greek_Mod [POU], Italian [perˈke], Pashto [DZEKA_CE], Persian [CHUN], Portuguese_ST [ˈpuɾkɨ], Provencal [PER-CO-QUE], Rumanian_List [FIINDCA], Russian [potomu_ˈʃto], Spanish [ˈpoɾke], Ukrainian [TOMU_SCO]

**Cognate Set "915":** Polish [bɔ], Slovak [BO], Lithuanian_ST [kɐˈda:ŋɡɪ]

**Cognate Set "748":** Latvian [tàdẽl_̜ka], Lithuanian_ST [TODEL_KAD]

**Cognate Set "704":** Byelorussian [TAGO_STO], Byelorussian [TAMU], Czech [protoʒɛ], Polish [dlaˈtɛgɔ_ʒɛ], Russian [potomu_ˈʃto], Serbocroatian [ZATO], Slovenian [ZATO], Ukrainian [TOMU_SCO], Danish [fʌˈd̥i], Dutch_List [ɔmˈdɑt], Frisian [OMDET], Frisian [TROCHDET], Norwegian [FORDI], Swedish [DARFOR_ATT]

**Cognate Set "584":** Hindi [KYOKE], Urdu [kjũ_ke]

**Cognate Set "365":** Faroese [tʊiː], Icelandic_ST [AF_THVI_AO]

**Cognate Set "282":** Czech [protoʒɛ], Danish [fʌˈd̥i], Norwegian [FORDI], Swedish [DARFOR_ATT], Catalan [pərˈkɛ], French [paʀs(ə)_k(ə)], Italian [perˈke], Portuguese_ST [ˈpuɾkɨ], Provencal [PER-CO-QUE], Spanish [ˈpoɾke]

**Cognate Set "1839":** Polish [dlaˈtɛgɔ_ʒɛ], Byelorussian [DZELJA]

**Cognate Set "1837":** Bulgarian [zɐˈʃtɔto], Serbocroatian [ZATO], Slovenian [ZATO]

**Cognate Set "1834":** Frisian [TROCHDET], Breton_List [DRE_MA]

**Cognate Set "1833":** Dutch_List [ɔmˈdɑt], Frisian [OMDET], Frisian [OMREDENEN]

- - 1. Basic Concept: "belly"

**Cognate Set "984":** Icelandic_ST [MAGI], Norwegian [MAVE], Swedish [MAGE]

**Cognate Set "709":** Danish [b̥u], Dutch_List [bœyk], Faroese [BUKUR], Frisian [buk], German [ba‿ux], Norwegian [buk], Swedish [bʉ:k]

**Cognate Set "2905":** Latvian [vę̂dęrs], Catalan [ˈbentrə], French [vɑ̃tʀ], Italian [ˈvɛntre], Portuguese_ST [ˈvẽtɾɨ], Provencal [VENTRE], Sardinian_C [BRENTI], Spanish [VIENTRE]

**Cognate Set "2821":** Hindi [PET], Urdu [peʈ]

**Cognate Set "2246":** Catalan [ˈpanʃə], Provencal [PANSO], Rumanian_List [PINTEC(E)]

**Cognate Set "201":** Serbocroatian [TRBUH], Slovenian [TREBUH]

**Cognate Set "110":** English [bɛlɪ], Irish_A [BOLG], Welsh_N [BOL]

**Cognate Set "1012":** Byelorussian [ZYVOT], Russian [ziˈvot], Ukrainian [ZYVIT]

***Cognate Set "220":** Byelorussian [BRUXA], Czech [br̝ɪxo], Polish [bʒux], Slovak [BRUCHO]

- - 1. Basic Concept: "big"

**Cognate Set "987":** Danish [ˈsd̥ʰoɐ̯], Faroese [ˈstɔurʊr], Icelandic_ST [stouːr], Norwegian [stor]

**Cognate Set "875":** Rumanian_List [ˈmare], Kurdish [mezin]

**Cognate Set "717":** Dutch_List [ɣrot], Frisian [GREAT], German [gro:s], Swedish [gru:v]

**Cognate Set "606":** Irish_A [MOR], Welsh_N [MAWR]

**Cognate Set "2906":** Breton_List [BRAS], Catalan [gran], French [gʀɑ̃], Italian [ˈgrande], Portuguese_ST [ˈɡɾɐ̃ðɨ], Provencal [GROS], Spanish [ˈɡɾande]

**Cognate Set "2822":** Hindi [bəɽɑː], Urdu [bəɽɛ]

**Cognate Set "2467":** Albanian_Standard [i_madh], Armenian_Mod [mɛʦ], Faroese [MIKILL], Greek_Mod [me̞ˈɣalo̞s], Sardinian_C [MANNU]

**Cognate Set "221":** Byelorussian [VJALIKI], Czech [vɛlkiː], Polish [ˈvʲɛlkʲi], Russian [veˈlikij], Serbocroatian [VELIK], Slovak [VEL_KY], Slovenian [VELIKA], Ukrainian [VELYKYJ]

- - 1. Basic Concept: "bird"

**Cognate Set "728":** Danish [ful], Dutch_List [ˈvoɣəl], Faroese [FUGLUR], Frisian [fu(:)gəl], German [ˈfo:gl̩], Icelandic_ST [fʏkl], Norwegian [FUGL], Swedish [ˈfo:gəl]

**Cognate Set "701":** Kurdish [balinde], Persian [pʰæɾænˈde], Urdu [pɾɪnd̪əh]

**Cognate Set "3028":** Breton_List [EVN], Irish_A [EAN], Welsh_N [ADERYN], Rumanian_List [pasăre], Spanish [ˈpaxaɾo]

**Cognate Set "2907":** Catalan [uˈseʎ], French [wazo], Italian [utˈtʃɛllo], Portuguese_ST [ˈavɨ], Provencal [awˈsɛl], Spanish [AVE]

**Cognate Set "2861":** Digor_Ossetic [marg], Pashto [MURGE], Persian [MORGH]

**Cognate Set "101":** Sardinian_C [PILLONI], Bulgarian [ˈptit͡sɐ], Byelorussian [PTUSKA], Czech [ptaːk], Latvian [putns], Lithuanian_ST [‘pɐukʲʃʲtʲɪs], Polish [ptak], Russian [ˈptitsa], Serbocroatian [PTICA], Slovak [VTAK], Slovenian [TICK], Ukrainian [PTAX]

- - 1. Basic Concept: "bite"

**Cognate Set "863":** Polish [ɡrɨɕʨ], Armenian_Mod [kəˈʦɛl], Pashto [CICEL], Serbocroatian [UGRISTI], Slovenian [GRIZT]

**Cognate Set "739":** Danish [ˈb̥iːðə], Dutch_List [ˈbɛitə(n)], English [baɪt], Faroese [BITA], Frisian [bitət], German [ˈba‿isn̩], Icelandic_ST [ˈpiːta], Norwegian [BITE], Swedish [ˈbi:ta]

**Cognate Set "585":** Digor_Ossetic [χwɐc-], Hindi [KHANA]

**Cognate Set "2908":** French [mɔʀdʀ], Italian [ˈmɔrdere], Portuguese_ST [muɾˈdeɾ], Provencal [MORDRE], Spanish [moɾˈðeɾ]

**Cognate Set "258":** Catalan [musəˈɣa], Rumanian_List [a_muʃˈka], Sardinian_C [MUSSIAI]

**Cognate Set "222":** Byelorussian [KUSACˈ], Czech [koʊ̯sat], Latvian [kôž], Lithuanian_ST [KASTI], Russian [ˈkusatʲ], Slovak [KUSNUT], Ukrainian [KUSATY]

- - 1. Basic Concept: "black"

**Cognate Set "750":** Danish [sɒ:d̥ʰ], Dutch_List [zwɑrt], Faroese [ˈsvaʃtʊr], German [ʃwart͜s], Icelandic_ST [ˈsvar̥tʏr], Norwegian [svart], Swedish [svart]

**Cognate Set "3030":** Breton_List [DU], Irish_A [DUBH], Welsh_N [DU]

**Cognate Set "2909":** Catalan [ˈnɛɣɾə], French [nwaʀ], Italian [ˈnero], Portuguese_ST [ˈneɡɾu], Provencal [NEGRE], Rumanian_List [ˈneɡru], Sardinian_C [NIEDDU], Spanish [ˈneɣɾo]

**Cognate Set "2824":** Hindi [ka:la:], Urdu [kɑlə]

**Cognate Set "2666":** Armenian_Mod [sɛv], Digor_Ossetic [saw], Persian [SIAH]

**Cognate Set "104":** Bulgarian [ˈt͡ʃɛrɛn], Byelorussian [CORNY], Czech [tʃɛrniː], Polish [ˈʧarnɨ], Russian [ˈtʃernɨj], Serbocroatian [CRN], Slovak [CIERNY], Slovenian [CRNU], Ukrainian [CORNYJ]

- - 1. Basic Concept: "blood"

**Cognate Set "760":** Danish [b̥loð], Dutch_List [blut], English [blʌd], Faroese [blɔuː], Frisian [blu.ət], German [blu:t], Icelandic_ST [plouːθ], Norwegian [BLOD], Swedish [blu:d]

**Cognate Set "3031":** Breton_List [GWAD], Welsh_N [GWAED]

**Cognate Set "2910":** Catalan [saŋ], French [sɑ̃], Italian [ˈsangwe], Portuguese_ST [ˈsɐ̃ɣɨ], Provencal [SANG], Rumanian_List [ˈsɨnʤe], Sardinian_C [SANGUINI], Spanish [ˈsaŋɡɾe]

**Cognate Set "2667":** Armenian_Mod [ɑˈɾɪ̯un], Latvian [asins]

**Cognate Set "105":** Bulgarian [krɤ̞f], Byelorussian [KROW], Czech [krɛf], Lithuanian_ST [KRAUJAS], Polish [krɛf], Russian [krovʲ], Serbocroatian [KRV], Slovak [KRV], Slovenian [KRI], Ukrainian [KROV]

****Cognate Set "2771":** Kurdish [xwîn], Pashto [VINA], Persian [xuːn], Urdu [χun]

- - 1. Basic Concept: "blow"

**Cognate Set "990":** Bulgarian [ˈduxɐm], Serbocroatian [DUVATI]

**Cognate Set "771":** Catalan [buˈfa], French [sufle], Italian [sofˈfjare], Portuguese_ST [suˈpɾaɾ], Provencal [BOUFA], Rumanian_List [a_sufla], Sardinian_C [SULAI], Spanish [soˈplaɾ], Danish [ˈb̥lεːsə], Dutch_List [ˈblazə(n)], English [bləʊ], Faroese [BLASA], Icelandic_ST [BLASA], Norwegian [BLASE], Swedish [ˈblo:sa]

**Cognate Set "586":** Persian [VAZIDAN], Hindi [BEHNA], Russian [ve.itʲ], Ukrainian [VIJATY], Dutch_List [ˈwayə(n)], Frisian [WAEIJE], German [ˈve:ən]

**Cognate Set "3032":** Breton_List [CˈHOUEZA], Irish_A [SEIDEADH], Welsh_N [GHWYTHU]

**Cognate Set "2668":** Greek_Mod [fiˈso̞], Latvian [pùš], Lithuanian_ST [PUSTI], Armenian_Mod [pʰəˈʧʰɛl], Urdu [pʰũknə], Czech [foʊ̯kat]

**Cognate Set "1843":** Russian [dutʲ], Ukrainian [DUTY], Slovak [DUT]

- - 1. Basic Concept: "bone"

**Cognate Set "781":** Danish [b̥en], Dutch_List [ɣrat], English [bəʊn], Faroese [bain], Frisian [bi.ən], German [das_Bein], Icelandic_ST [peiːn], Norwegian [ben], Swedish [beːn]

**Cognate Set "707":** Latvian [kaũls], Lithuanian_ST [KAULAS]

**Cognate Set "2635":** Armenian_Mod [vosˈkoɾ], Breton_List [ASKOURN], Catalan [ɔs], Digor_Ossetic [ɐstɐg], French [ɔs], Italian [ˈɔsso], Kurdish [hestî], Persian [ostoxɒn], Portuguese_ST [ˈosu], Provencal [ɔs], Rumanian_List [os], Sardinian_C [OSSU], Spanish [ˈweso], Welsh_N [ASGWRN]

**Cognate Set "204":** Pashto [HED], Hindi [ɦəɖːiː], Urdu [həɖːi]

***Cognate Set "106":** Albanian_Standard [kockë], Bulgarian [kɔst], Byelorussian [KOSTKA], Czech [kost], Polish [kɔɕʨ], Russian [kostʲ], Serbocroatian [KOST], Slovak [KOST], Slovenian [KUST], Ukrainian [KISTˈ]

- - 1. Basic Concept: "breast"

**Cognate Set "999":** Latvian [krùts], Lithuanian_ST [krūtis]

**Cognate Set "995":** Welsh_N [brɔn], Dutch_List [borst], English [breast], Faroese [brœst], German [Brust], Icelandic_ST [ˈb̥rjousd̥], Norwegian [bryst]

**Cognate Set "993":** Italian [ˈpoppa], Latvian [pups]

**Cognate Set "991":** French [mamɛl], Italian [mamˈmɛlla], Irish_A [mama]

**Cognate Set "2495":** Kurdish [pêsîr], Persian [pistān]

**Cognate Set "2239":** French [tetɔ̃], Italian [ˈtetta], Rumanian_List [ƫâƫă], Spanish [teta]

***Cognate Set "1001":** Armenian_Mod [stin], Greek_Mod [ˈstiθo̞s], Urdu [pɪstɑn]

- - 1. Basic Concept: "breathe"

**Cognate Set "820":** Pashto [TANAFFUS_KAVEL], Persian [NAFAS_KASHIDAN]

**Cognate Set "789":** Dutch_List [ˈadəmə(n)], Frisian [AMJE], German [ˈa:tmən]

**Cognate Set "2912":** Breton_List [ALANAT], Irish_A [ANAL_DO_THARRAINMGT], Welsh_N [ANADLU], Catalan [ələˈna], Provencal [ALENA], Danish [ˈʌnə], Faroese [ANDA], Icelandic_ST [ˈanta], Swedish [ˈandas]

**Cognate Set "2669":** Hindi [SAS+LENA], Urdu [sɑ̃s_lenə]

**Cognate Set "1847":** French [ʀɛspiʀe], Italian [respiˈrare], Portuguese_ST [ɾɨʃpiˈɾaɾ], Provencal [RESPIRA], Rumanian_List [a_respira], Sardinian_C [RESPIRAI], Spanish [respiˈɾaɾ]

**Cognate Set "107":** Bulgarian [ˈdiʃɐm], Byelorussian [DYXACˈ], Czech [diːxat], Latvian [dvašõ], Polish [ɔdˈdɨxaʨ], Russian [ˈduʃatʲ], Serbocroatian [DISATI], Slovak [DYCHAT], Slovenian [DIHAT], Ukrainian [VIDDYXATY]

- - 1. Basic Concept: "burn"

**Cognate Set "794":** Danish [ˈb̥ʁanə], Dutch_List [ˈbrɑndə(n)], English [bɜ:n], Faroese [BRENNA], Frisian [ˈbanə], German [ˈbrɛnən], Icelandic_ST [BRENNA], Norwegian [BRENNE], Swedish [ˈbrɛna]

**Cognate Set "388":** Italian [ˈardere], Portuguese_ST [ɐɾˈdeɾ], Rumanian_List [a_arde], Spanish [ARDER]

**Cognate Set "3033":** Breton_List [LESKI], Welsh_N [LLOSGI]

**Cognate Set "303":** Digor_Ossetic [sodz-], Kurdish [sotîn], Kurdish [şewtî], Pashto [SVADZEDEL], Persian [SUKHTAN]

**Cognate Set "2826":** Hindi [dʒəlna], Urdu [ʤəlnɑ]

**Cognate Set "2486":** Albanian_Standard [djeg], Latvian [de̥g], Lithuanian_ST [DEGTI]

**Cognate Set "2240":** French [bʀyle], Italian [bruˈtʃare], Provencal [BRULA], Sardinian_C [ABRUZAI]

**Cognate Set "1017":** Bulgarian [ɡoˈrjɐ], Czech [hor̝ɛt], Russian [goˈrʲetʲ], Slovak [ZHORET], Slovenian [ZGORET], Ukrainian [GORITY]

**Cognate Set "1016":** Catalan [krəˈma], Spanish [keˈmaɾ]

**Cognate Set "1015":** Byelorussian [PALICˈ], Polish [ˈpaʎiʨ], Serbocroatian [PALITI], Slovak [PALIT]

- - 1. Basic Concept: "child"

**Cognate Set "798":** Dutch_List [kɪnt], Frisian [kin], German [kɪnt]

**Cognate Set "390":** French [ɑ̃fɑ̃], Italian [fanˈtʃullo], Provencal [ENFANT]

**Cognate Set "309":** Danish [b̥ɑn], Faroese [BARN], Icelandic_ST [ˈpartn], Norwegian [BARN], Swedish [bɑːrn], Frisian [bɛ̃ːn]

**Cognate Set "2913":** Catalan [kriəˈturə], Portuguese_ST [kɾiˈɐ̃sɐ]

**Cognate Set "2827":** Hindi [BECCA], Persian [BACHCHE], Urdu [bəʧəh]

**Cognate Set "259":** Catalan [nɔi], Catalan [nɛn], Portuguese_ST [MENINO], Spanish [ˈniɲo]

**Cognate Set "108":** Bulgarian [dɛˈtɛ], Byelorussian [DZICE], Czech [ɟiːcɛ], Polish [ˈʥɛʦkɔ], Russian [rʲeˈbʲonok], Serbocroatian [DETE], Slovak [DIETA], Ukrainian [DYTYNA]

- - 1. Basic Concept: "cloud"

**Cognate Set "805":** Dutch_List [wɔlk], Frisian [volk], German [ˈvɔlkə]

**Cognate Set "550":** Hindi [bɑːdəl], Urdu [bɑd̪əl]

**Cognate Set "310":** Danish [sg̥ʰy], Faroese [skʊdʒ], Icelandic_ST [SKY], Norwegian [sky], Swedish [ʃy:]

**Cognate Set "3035":** Breton_List [KOUMOUL], Welsh_N [CWMWL]

**Cognate Set "2914":** Catalan [ˈnußul], French [nyaʒ], Italian [ˈnuvola], Portuguese_ST [ˈnuvɐ̃j], Provencal [NIVO], Rumanian_List [nor], Sardinian_C [NUI], Spanish [ˈnuβe]

**Cognate Set "2672":** Armenian_Mod [ɑmp], Greek_Mod [ˈsine̞ˌfo̞], Kurdish [ewr], Latvian [padebesis], Lithuanian_ST [DEBESIS], Persian [ABR]

**Cognate Set "223":** Byelorussian [XMARA], Polish [ˈxmura], Ukrainian [XMARA]

**Cognate Set "109":** Bulgarian [ˈɔblɐk], Czech [oblak], Polish [ˈɔbwɔk], Serbocroatian [OBLAK], Slovak [OBLAK], Slovenian [OBLAK]

- - 1. Basic Concept: "cold"

**Cognate Set "812":** Danish [g̥ʰʌl], Dutch_List [kɑut], English [kəʊld], Faroese [KALDUR], Frisian [kɔ:t], German [kalt], Icelandic_ST [ˈkʰaltʏr], Norwegian [KALD], Swedish [kal]

**Cognate Set "611":** Irish_A [FUAR], Welsh_N [OER], Latvian [aũksts]

**Cognate Set "470":** Armenian_Mod [ʦʰuɾt], Latvian [sal̂ts], Lithuanian_ST [SALTAS], Digor_Ossetic [wazal], Kurdish [sar], Pashto [SOR], Persian [SARD]

**Cognate Set "2915":** Catalan [fɾɛt], French [fʀwɑ], Italian [ˈfreddo], Portuguese_ST [ˈfɾiu], Provencal [FRE], Sardinian_C [FRIRU], Spanish [ˈfɾio]

**Cognate Set "2830":** Hindi [THENDA], Urdu [səɾd̪]

**Cognate Set "189":** Slovenian [hladen]

**Cognate Set "1789":** Bulgarian [stoˈdɛn], Czech [stʊdɛni], Slovak [STUDENY]

**Cognate Set "111":** Byelorussian [SˈCJUDZENA], Czech [xladni], Russian [xoˈlodnɨj], Serbocroatian [HLADNO], Ukrainian [XOLODNO]

- - 1. Basic Concept: "come"

**Cognate Set "817":** Catalan [bəˈni], French [vəniʀ], Italian [veˈnire], Portuguese_ST [viɾ], Provencal [VENI], Rumanian_List [a_veˈni], Sardinian_C [BENNI], Spanish [beˈniɾ], Danish [ˈg̥ʰʌmə], Dutch_List [ˈkomə(n)], English [kʌm], Faroese [KOMA], Frisian [ˈkomə], German [ˈkɔmən], Icelandic_ST [ˈkʰɔːma], Norwegian [KOMME], Swedish [ˈkɔmːa]

**Cognate Set "551":** Hindi [aːn̪aː], Urdu [ʔɑnə]

**Cognate Set "3037":** Breton_List [DONET], Welsh_N [doːd]

**Cognate Set "112":** Bulgarian [ˈidvɐm], Byelorussian [PRYXODZICˈ], Czech [pr̝̊ɪjiːt], Lithuanian_ST [ATEITI], Polish [pʃɨjɕʨ], Polish [pʃɨˈxɔʥiʨ], Russian [prixoˈditʲ], Serbocroatian [DOCI], Slovak [PRIST], Slovenian [PRTDI], Ukrainian [PRYXODYTY]

- - 1. Basic Concept: "count"

**Cognate Set "823":** Danish [ˈd̥ʰεlə], Dutch_List [tellən], Faroese [TELJA], Frisian [ˈtɛlə], German [ˈt͜sɛ:lən], Icelandic_ST [TELJA], Norwegian [TELLE]

**Cognate Set "442":** Digor_Ossetic [nimaj-], Greek_Mod [me̞ˈtro̞]

**Cognate Set "2773":** Hindi [GINNA], Urdu [ɡɪnnə]

**Cognate Set "224":** Byelorussian [LIZYCˈ], Polish [ˈpɔʎiʧɨʨ], Ukrainian [CYSLYTY]

**Cognate Set "202":** Czech [potʃiːtat], Latvian [skàita], Lithuanian_ST [SKAICIUOTI], Russian [stʃɨˈtatʲ], Slovak [POCITAT], Slovenian [STET]

***Cognate Set "2916":** English [kaʊnt], Catalan [kumˈta], French [kɔ̃te], Italian [konˈtare], Portuguese_ST [kõˈtaɾ], Provencal [COUMTA], Sardinian_C [KONTAI], Spanish [konˈtaɾ]

***Cognate Set "1051":** Ukrainian [RAXUBATY], Serbocroatian [RACUNATI], Swedish [ˈrɛ:kna]

***Cognate Set "1039":** Armenian_Mod [HAMREL], Pashto [SMEREL], Persian [SHOMORDAN]

***Cognate Set "1004":** Rumanian_List [a_număra], Albanian_Standard [numëroj], Portuguese_ST [NUMERAR], Breton_List [NIVERI]

- - 1. Basic Concept: "cut"

**Cognate Set "833":** Dutch_List [ˈsnɛidə(n)], Frisian [ˈsnɛiə], German [ˈʃna‿idn̩]

**Cognate Set "2831":** Danish [ˈsg̥ʰεːʌ], Faroese [SKERA], Icelandic_ST [ˈscɛːra], Norwegian [SKJAERE], Swedish [ˈsko:ra], Armenian_Mod [kətˈɾɛl], Digor_Ossetic [kɐrd-], Hindi [KATNA], Urdu [kɑʈnə], Portuguese_ST [kuɾˈtaɾ], Spanish [koɾˈtaɾ]

**Cognate Set "260":** French [kupe], Provencal [COUPA]

**Cognate Set "1854":** Catalan [təˈʎa], Italian [taʎˈʎare], Rumanian_List [a_tăia]

**Cognate Set "115":** Bulgarian [ˈrɛʒɐ], Byelorussian [REZACˈ], Czech [r̝ɛzat], Russian [ˈrʲezatʲ], Slovak [REZAT], Slovenian [REZAT], Ukrainian [RIZATY]

**Cognate Set "1021":** Sardinian_C [SEGAI], Serbocroatian [SECI]

- - 1. Basic Concept: "day"

**Cognate Set "845":** Danish [dɛ], Dutch_List [dɑχ], English [deɪ], Faroese [ˈdɛavʊr], Frisian [dai], German [ta:k], Icelandic_ST [ˈtaːɣʏr], Norwegian [DAG], Swedish [dɑː(g)]

**Cognate Set "444":** Armenian_Mod [oɾ], Greek_Mod [ˈme̞ra]

**Cognate Set "2500":** Albanian_Standard [ditë], Breton_List [DEIZ], Bulgarian [dɛn], Byelorussian [DZENˈ], Catalan [ˈdiə], Czech [dɛn], French [ʒuʀ], Hindi [d̪ɪn̪], Italian [ˈdʒorno], Latvian [dìena], Lithuanian_ST [dʲjɛˈnɐ], Polish [ʥɛɲ], Portuguese_ST [ˈdiɐ], Provencal [JOUR], Rumanian_List [zi], Russian [dʲenʲ], Sardinian_C [DI], Serbocroatian [DAN], Slovak [DEN], Slovenian [DAN], Spanish [ˈdia], Ukrainian [DENˈ], Urdu [d̪ɪn], Welsh_N [dɨːð]

- - 1. Basic Concept: "die"

**Cognate Set "761":** Armenian_Mod [mɛrˈnɛl], Breton_List [MERVEL], Bulgarian [oˈmirɐm], Byelorussian [PAMIRACˈ], Catalan [muˈri], Czech [ʊmiːrat], Digor_Ossetic [mɐl-], French [muʀiʀ], Hindi [mərna], Italian [moˈrire], Kurdish [mirin], Latvian [mir̃st], Lithuanian_ST [‘mʲiɾʲtʲɪ], Pashto [MREL], Persian [MORDAN], Polish [uˈmʲɛraʨ], Portuguese_ST [muˈʀeɾ], Provencal [DEBANA], Provencal [MOURI], Rumanian_List [a_muˈri], Russian [umiˈratʲ], Sardinian_C [MORRI], Serbocroatian [UMRETI], Slovak [UMIERAT], Slovenian [UMRT], Spanish [moˈɾiɾ], Ukrainian [UMYRATY], Urdu [məɾnə], Welsh_N [MARW]

**Cognate Set "445":** Greek_Mod [pe̞ˈθe̞no̞], Albanian_Standard [vdes]

**Cognate Set "1023":** Dutch_List [ˈstɛrvə(n)], Frisian [FORSTJERRE], German [ˈʃtɛrbn̩]

****Cognate Set "855":** Danish [do], Faroese [DOYGGJA], Frisian [dɪ.ə], Icelandic_ST [ˈteiːja], Norwegian [dø], Swedish [dø:], English [daɪ]

- - 1. Basic Concept: "dig"

**Cognate Set "864":** Bulgarian [koˈpajɐ], Byelorussian [KAPACˈ], Czech [kopat], Polish [ˈkɔpat͡ɕ], Serbocroatian [KOPATI], Slovak [KOPAT], Slovenian [KOPAT], Ukrainian [KOPATY], Greek_Mod [ˈskavo̞]

**Cognate Set "3040":** Breton_List [KLEUZA], Welsh_N [CLODDIO_(DIG_FOR_GOLD)]

**Cognate Set "2917":** Breton_List [KAVA], Catalan [kəˈßa], Italian [skaˈvare], Portuguese_ST [kɐˈvaɾ], Provencal [CAVA], Rumanian_List [a_ek.skaˈva], Sardinian_C [SKAVAI], Spanish [kaˈβaɾ]

**Cognate Set "2832":** Hindi [KHODNA], Urdu [kʰod̪nə]

**Cognate Set "1857":** Irish_A [ROMHAR], Russian [rɨtʲ]

**Cognate Set "1856":** Danish [ˈg̥ʁɑːvə], Faroese [GRAVA], Frisian [ˈgra:və], German [ˈgra:bn̩], Icelandic_ST [ˈkraːva], Norwegian [GRAVE], Swedish [ˈgrɛ:va]

- - 1. Basic Concept: "dirty"

**Cognate Set "873":** Dutch_List [vœyl], Frisian [fi:s]

**Cognate Set "391":** Irish_A [SALACH], French [sal], Provencal [SALE]

**Cognate Set "311":** Danish [b̥eˈsg̥ʰid̥], Faroese [SKITIN], Icelandic_ST [ˈsciːtʏːɣʏr], Norwegian [SKIDDEN]

**Cognate Set "2918":** Catalan [pɔrk], Italian [ˈspɔrko], Portuguese_ST [PORCO]

**Cognate Set "2775":** Hindi [MELA], Urdu [mɛlə]

**Cognate Set "225":** Byelorussian [BRUDNY], Polish [ˈbrudnɨ], Slovak [BRUDNY], Ukrainian [BRUDNYJ]

**Cognate Set "1869":** Portuguese_ST [ˈsuʒu], Spanish [ˈsuθjo]

**Cognate Set "1866":** Catalan [bɾut], Sardinian_C [BRUTTU]

**Cognate Set "1865":** Icelandic_ST [ˈouːr̥eitn], Swedish [ˈure:n]

***Cognate Set "421":** German [ˈʃmʊt͜sɪç], Swedish [SMUTSIG]

- - 1. Basic Concept: "dog"

**Cognate Set "986":** Armenian_Mod [ʃun], Breton_List [KI], Danish [hun], Dutch_List [hɔnt], Faroese [HUNDUR], French [ʃi_ɛ̃], Frisian [hun], German [hʊnt], Icelandic_ST [ˈhʏntʏr], Italian [ˈkane], Kurdish [seg], Latvian [suns], Lithuanian_ST [ʃʊo], Norwegian [hund], Pashto [SPAJ], Persian [sæg], Portuguese_ST [kɐ̃ũ], Provencal [CHIN], Rumanian_List [ˈkɨjne], Sardinian_C [KANI], Swedish [hɵnd], Welsh_N [ki]

**Cognate Set "93":** Byelorussian [SABAKA], Russian [soˈbaka], Ukrainian [SOBAKA]

**Cognate Set "552":** Hindi [kʊt̪ːaː], Urdu [kʊt̪ɑ]

**Cognate Set "2258":** Catalan [gos], Provencal [gus]

**Cognate Set "2257":** Digor_Ossetic [kuj], Kurdish [kûçik]

**Cognate Set "117":** Czech [pɛs], Polish [pʲɛs], Russian [pʲos], Serbocroatian [PAS], Slovak [PES], Slovenian [PAS], Ukrainian [PES]

- - 1. Basic Concept: "drink"

**Cognate Set "883":** Danish [ˈdʁεg̥ʰə], Dutch_List [ˈdrɪŋkə(n)], English [drɪŋk], Faroese [DREKKA], German [ˈtrɪŋkn̩], Icelandic_ST [DREKKA], Norwegian [drikke], Swedish [ˈdrika]

**Cognate Set "802":** Digor_Ossetic [niwaz-], Persian [NUSHIDAN]

**Cognate Set "710":** Latvian [dzer], Lithuanian_ST [‘ɡʲæ:ɾʲtʲɪ]

**Cognate Set "2514":** Albanian_Standard [pi], Armenian_Mod [əmpem], Breton_List [EVA], Bulgarian [ˈpijɐ], Byelorussian [PICˈ], Catalan [ˈbɛwɾə], Czech [piːt], French [bwaʀ], Greek_Mod [ˈpino̞], Hindi [pinɑ], Irish_A [OL], Italian [ˈbere], Polish [pʲiʨ], Portuguese_ST [bɨˈbeɾ], Provencal [CHOURLA], Provencal [ˈbewre], Rumanian_List [a_beː], Russian [pitʲ], Sardinian_C [BUFFAI], Serbocroatian [PITI], Slovak [PIT], Slovenian [PIT], Spanish [beˈβeɾ], Ukrainian [PYTY], Urdu [pinə], Welsh_N [ˈəvɛd]

- - 1. Basic Concept: "dry"

**Cognate Set "890":** Dutch_List [droχ], English [draɪ], Frisian [drux], German [ˈtrɔkn̩]

**Cognate Set "2919":** Breton_List [SECˈH], Welsh_N [SYCH], Catalan [sɛk], French [sɛk], Portuguese_ST [ˈseku], Provencal [SE], Sardinian_C [SIKKU], Spanish [ˈseko]

**Cognate Set "2776":** Bulgarian [sux], Byelorussian [SUXI], Czech [sʊxiː], Latvian [sàuss], Lithuanian_ST [SAUSAS], Polish [ˈsuxɨ], Russian [suˈxoj], Serbocroatian [SUV], Slovak [SUCHY], Slovenian [SUHO], Ukrainian [SUXYJ], Albanian_Standard [i_thatë], Digor_Ossetic [χuskˈɐ], Hindi [su:kʰa], Pashto [VUC], Persian [KHOSHK], Urdu [sukʰə]

**Cognate Set "2636":** Irish_A [TIRIM], Danish [d̥ʰo], Faroese [TURRUR], Icelandic_ST [θʏrː], Norwegian [TORR], Swedish [tɔr]

**Cognate Set "1795":** Italian [aʃˈʃutto], Rumanian_List [uscat]

- - 1. Basic Concept: "dull"

**Cognate Set "711":** Latvian [neass], Lithuanian_ST [NEASTRUS]

**Cognate Set "702":** Persian [KOND], Urdu [kʊ̃d̪]

**Cognate Set "3042":** English [dʌl], Breton_List [DALLA]

**Cognate Set "2920":** Portuguese_ST [embotado], Spanish [emboˈtaðo]

**Cognate Set "262":** French [emuse], Provencal [MOUTU]

**Cognate Set "1875":** Italian [otˈtuzo], Breton_List [TALTOUZA]

**Cognate Set "1874":** Danish [slø̞w], Norwegian [SLOV]

**Cognate Set "118":** Dutch_List [stɔmp], Frisian [stomp], German [ʃtʊmp͜f], Bulgarian [tɤ̞p], Byelorussian [TUPY], Czech [tʊpiː], Polish [ˈtɛ̃pɨ], Russian [tuˈpoj], Serbocroatian [TUP], Slovak [TUPY], Ukrainian [TUPYJ]

- - 1. Basic Concept: "dust"

**Cognate Set "910":** Danish [sd̥ʰø̞w], Dutch_List [stɔf], Faroese [støv], Frisian [stɔf], German [ʃta‿up], Norwegian [STOV], Swedish [stɔft]

**Cognate Set "1878":** Byelorussian [PYL], Latvian [putekl̜I], Polish [pɨw], Russian [pɨl], Ukrainian [NYL]

***Cognate Set "2921":** Albanian_Standard [pluhur], Breton_List [POULTR], Catalan [pols], French [pusjɛʀ], Italian [ˈpolvere], Portuguese_ST [puˈejɾɐ], Provencal [POUSSO], Rumanian_List [pulbere], Sardinian_C [PRUINI], Spanish [ˈpolβo]

***Cognate Set "1801":** Pashto [GARZ], Urdu [ɡəɾd̪]

***Cognate Set "1780":** Irish_A [DUSTA], English [dʌst], Faroese [DUST], Hindi [DHUL], Lithuanian_ST [DULKES]

***Cognate Set "119":** Rumanian_List [praf], Bulgarian [prax], Czech [prax], Serbocroatian [PRASINA], Slovak [PRACH], Slovenian [PRAH], Ukrainian [POROX]

- - 1. Basic Concept: "ear"

**Cognate Set "914":** Albanian_Standard [vesh], Armenian_Mod [ɑˈkɑnʤ], Bulgarian [uˈxɔ], Byelorussian [VUXA], Catalan [uˈrɛʎə], Czech [ʊxo], Danish [ˈø̞:ʌ], Dutch_List [or], English [ɪə], Faroese [ˈɔira], French [ɔʀɛj], Frisian [ɔ.ər], German [o:ɐ̯], Greek_Mod [afˈti], Icelandic_ST [ˈeiːra], Italian [oˈrekkjo], Latvian [àuss], Lithuanian_ST [ɐʊˈsʲɪs], Norwegian [øre], Polish [ˈuxɔ], Portuguese_ST [oˈɾɐʎɐ], Provencal [awˈɾeʎo], Rumanian_List [uˈreke], Russian [ˈuxo], Sardinian_C [URIGA], Serbocroatian [UVO], Slovak [UCHO], Slovenian [USESU], Spanish [oˈɾexa], Swedish [œ̞ːra], Ukrainian [VUXO]

**Cognate Set "2833":** Hindi [KAN], Urdu [kɑn]

**Cognate Set "1868":** Digor_Ossetic [ʁos], Kurdish [guh], Pashto [GVAZ], Persian [ɡuːʃ]

**Cognate Set "1026":** Ukrainian [SLUX], Irish_A [CLUAS], Welsh_N [CLUST]

- - 1. Basic Concept: "earth"

**Cognate Set "918":** Danish [joɐ̯], Dutch_List [ˈardə], English [ɜ:θ], Faroese [jørð], Frisian [ˈi.ədə], German [ˈe:ɐ̯də], Icelandic_ST [jœrð], Norwegian [jord], Swedish [juːrd]

**Cognate Set "366":** Faroese [mɔld], Icelandic_ST [mold]

**Cognate Set "3045":** Breton_List [DOUAR], Welsh_N [DAEAR]

**Cognate Set "2923":** Catalan [ˈtɛrə], French [tɛʀ], Italian [ˈtɛrra], Portuguese_ST [ˈtɛʀɐ], Provencal [TERRO], Sardinian_C [TERRA], Spanish [ˈtjera]

**Cognate Set "1805":** Irish_A [CRE], Welsh_N [PRIDD]

**Cognate Set "1799":** Czech [PUDA], Slovak [PODA]

**Cognate Set "1796":** Kurdish [xak], Pashto [XAK]

***Cognate Set "2523":** Urdu [zəmin], Bulgarian [zɛˈmja], Czech [zɛmɲɛ], Latvian [zeme], Lithuanian_ST [‘ʒʲæ:mʲe:], Polish [ˈʑɛmʲa], Russian [zʲemˈlʲa], Serbocroatian [ZEMLJA], Slovak [ZEM], Slovenian [ZEMLA], Ukrainian [ZEMLJA], Persian [zamin]

- - 1. Basic Concept: "eat"

**Cognate Set "925":** Albanian_Standard [ha], Armenian_Mod [uˈtɛl], Bulgarian [DA_JADE], Byelorussian [ESˈCI], Czech [jiːst], Dutch_List [etə(n)], English [i:t], Faroese [ETA], Frisian [ˈitə], German [ˈɛsn̩], Icelandic_ST [ETA], Icelandic_ST [ˈpɔrða], Latvian [ę̂d], Polish [jɛɕʨ], Russian [jestʲ], Serbocroatian [JESTI], Slovak [JEST], Slovenian [JEST], Swedish [ˈɛ:ta], Ukrainian [JISTY]

**Cognate Set "314":** Danish [sb̥ʰi:sə], Norwegian [spise]

**Cognate Set "2924":** Portuguese_ST [kuˈmeɾ], Spanish [koˈmeɾ]

**Cognate Set "263":** Catalan [mənˈʒa], French [mɑ̃ʒe], Italian [manˈdʒare], Provencal [MANJA], Rumanian_List [a_mɨnˈka]

**Cognate Set "2528":** Hindi [kʰana], Urdu [kʰɑnə]

**Cognate Set "2005":** Digor_Ossetic [χwɐr-], Kurdish [xwarin], Pashto [XVAREL], Persian [KHORDAN]

- - 1. Basic Concept: "egg"

**Cognate Set "2834":** Hindi [ENDA], Urdu [ʔənɖɑ]

****Cognate Set "2075":** Albanian_Standard [vezë], Armenian_Mod [ʣu], Breton_List [VI], Bulgarian [jɐʲˈt͡sɛ], Byelorussian [JAJKO], Catalan [ɔu], Czech [vɛjt͡sɛ], Danish [εg̥], Digor_Ossetic [ajkɐ], Dutch_List [ɛi], Faroese [ɛɡː], French [œf], Frisian [EIKE], Frisian [aːɨ], German [a‿i], Greek_Mod [avˈɣo̞], Icelandic_ST [ɛkː], Irish_A [UBH], Italian [ˈwɔvo], Kurdish [hêk], Norwegian [EGG], Pashto [HAGEJ], Polish [ˈjajkɔ], Portuguese_ST [ˈovu], Provencal [IOU], Rumanian_List [ow], Russian [jajˈtso], Sardinian_C [OU], Serbocroatian [JAJE], Slovak [VAJCE], Slovenian [JAJCE], Spanish [ˈweβo], Swedish [ɛg], Ukrainian [JAJCE], Welsh_N [WY], English [ɛg]

- - 1. Basic Concept: "eye"

**Cognate Set "928":** Albanian_Standard [sy], Armenian_Mod [ɑʧʰkʰ], Bulgarian [oˈkɔ], Byelorussian [VOKA], Catalan [uʎ], Czech [oko], Danish [ˈʌjə], Dutch_List [oχ], English [aɪ], Faroese [ˈɛija], French [œj], Frisian [jɛx], German [ˈa‿ugə], Greek_Mod [ˈmati], Hindi [ɑ̃ŋkʰ], Icelandic_ST [ˈøyːɣa], Italian [ˈɔkkjo], Latvian [acs], Lithuanian_ST [ɐˈkʲɪs], Norwegian [øye], Polish [ˈɔkɔ], Portuguese_ST [ˈoʎu], Provencal [UEI], Rumanian_List [ˈoki], Sardinian_C [OGU], Serbocroatian [OKO], Slovak [OKO], Slovenian [OKO], Spanish [ˈoxo], Swedish [ˈøːɡa], Ukrainian [OKO], Urdu [ʔɑ̃kʰ]

**Cognate Set "3047":** Breton_List [LAGAD], Welsh_N [LLYGAD]

**Cognate Set "2778":** Digor_Ossetic [cɐstɐ], Kurdish [çav], Persian [tʃʰæʃm]

- - 1. Basic Concept: "fall"

**Cognate Set "930":** Danish [ˈfalə], Dutch_List [ˈvɑlə(n)], English [fɔ:l], Faroese [ˈfɛdla], Frisian [ˈfɔ(:)lə], German [ˈfalən], Icelandic_ST [FALLA], Norwegian [FALLE], Swedish [ˈfɛla], Lithuanian_ST [NUPULTI]

**Cognate Set "825":** Greek_Mod [ˈpe̞fto̞], Persian [OFTADAN]

**Cognate Set "392":** French [tɔ̃be], Provencal [TOUMBA]

**Cognate Set "2925":** Catalan [ˈkauwrə], Italian [kaˈdere], Portuguese_ST [kɐˈiɾ], Rumanian_List [a_kəˈde̯a], Spanish [kaˈeɾ]

**Cognate Set "1885":** Latvian [krìt], Lithuanian_ST [(NU)KRISTI]

**Cognate Set "1883":** Hindi [GIRNA], Urdu [ɡɪɾnə]

**Cognate Set "120":** Bulgarian [ˈpadɐm], Byelorussian [PADACˈ], Czech [UPADNOUTI], Czech [padat], Polish [ˈspadaʨ], Russian [ˈpadatʲ], Serbocroatian [PASTI], Slovak [PADAT], Slovenian [PADE], Ukrainian [PADATY]

- - 1. Basic Concept: "far"

**Cognate Set "936":** Armenian_Mod [hɛˈru], Danish [fjɛɐ̯n], Dutch_List [vɛr], English [fɑ:], Frisian [fi.ər], Swedish [ˈfjæran]

**Cognate Set "712":** Latvian [tâlu], Lithuanian_ST [TOLIMAS]

**Cognate Set "3049":** Breton_List [DIABELL], Breton_List [PELL], Welsh_N [PELL]

**Cognate Set "2836":** Hindi [DUR], Pashto [LIRI], Persian [DUR], Urdu [d̪uɾ]

**Cognate Set "2779":** Bulgarian [dɐˈlɛt͡ʃɛn], Byelorussian [DALEKI], Czech [dalɛkiː], Polish [daˈlɛkɔ], Russian [dalʲeˈko], Serbocroatian [DALEK], Slovak [D_ALEKY], Slovenian [DALEC], Ukrainian [DALEKO]

**Cognate Set "1028":** Faroese [LANGT_BURTUR], Icelandic_ST [LANGT_(I)_BURTU], Norwegian [LANGT_BORTE], Catalan [ʎuɲ], French [lwɛ̃], Italian [lonˈtano], Portuguese_ST [ˈlõʒɨ], Provencal [LUEN]

- - 1. Basic Concept: "fat"

**Cognate Set "264":** Catalan [gras], Catalan [greʃ], French [gʀɛs], Italian [ˈgrasso], Provencal [GRAISSO], Rumanian_List [grăsime], Sardinian_C [GRASSU], Spanish [ˈɡɾasa]

**Cognate Set "226":** Bulgarian [tlɤstiˈna], Byelorussian [TUK], Czech [tʊk], Latvian [tàuki], Lithuanian_ST [TAUKAI], Polish [twuʃʧ], Slovak [TLSTY], Ukrainian [TOVSC]

**Cognate Set "1806":** Irish_A [BLONAG], Welsh_N [BLONEG]

**Cognate Set "121":** Serbocroatian [MAST], Slovenian [MAST]

****Cognate Set "947":** Danish [fɛd̥], Dutch_List [vɛt], English [fæt], Faroese [FITI], Frisian [fɛt], Icelandic_ST [fita], Icelandic_ST [ˈfeiːtɪ], Norwegian [fett], Swedish [fet], German [fɛt]

****Cognate Set "2837":** Persian [tʃærbiː], Hindi [CERBI], Urdu [ʧəɾbi]

- - 1. Basic Concept: "father"

**Cognate Set "2655":** Albanian_Standard [baba], Hindi [BAP], Urdu [bɑp]

**Cognate Set "2539":** Czech [otɛts], Polish [ˈɔjʨɛʦ], Russian [oˈtʲets], Serbocroatian [OTAC], Slovak [OTEC], Slovenian [OCE]

**Cognate Set "2417":** Armenian_Mod [hɑɪ̯ɾ], Catalan [ˈpaɾə], Danish [ˈfɛ:ðʌ], Digor_Ossetic [fidɐ], Dutch_List [ˈvadər], English [fɑ:ðə], Faroese [FADIR], French [pɛʀ], Frisian [fa:r], German [ˈfa:tɐ], Greek_Mod [paˈte̞ras], Hindi [pit̪ɑː], Icelandic_ST [ˈfaːðɪr], Irish_A [ATHAIR], Italian [ˈpadre], Norwegian [FAR], Pashto [PLAR], Persian [PEDAR], Portuguese_ST [pai], Provencal [ˈpajɾe], Spanish [ˈpaðɾe], Swedish [ˈfɑ:dər]

**Cognate Set "205":** Byelorussian [BACˈKA], Ukrainian [BATˈKO]

***Cognate Set "3050":** Bulgarian [ˈtatko], Latvian [tę̃vs], Lithuanian_ST [‘tʲe:vɐs], Breton_List [TAD], Welsh_N [TAD], Rumanian_List [ˈtatə]

- - 1. Basic Concept: "fear"

**Cognate Set "958":** Dutch_List [ˈvrezə(n)], English [fɪə]

**Cognate Set "943":** Breton_List [KAOUT_AON], Welsh_N [OFNI]

**Cognate Set "367":** French [kʀɛ̃dʀ], Provencal [CREGNE], Faroese [RAEDAST], Faroese [VERA_RADDUR], Icelandic_ST [HRAEOASK], Icelandic_ST [VERA_HRAEDDR], Norwegian [VAERE_REDD]

**Cognate Set "315":** German [ˈfʏrçtn̩], Danish [ˈfʁœ̝g̥d̥ʰə], Swedish [ˈfrɵkta], Swedish [ˈfɑ:rho:ga]

**Cognate Set "2927":** Catalan [ˈtemə], Italian [teˈmere], Portuguese_ST [tɨˈmeɾ], Provencal [TEME], Rumanian_List [a_se_teme], Sardinian_C [TIMMI], Spanish [teˈmeɾ]

**Cognate Set "2780":** Byelorussian [BAJACCA], Czech [baːt_se], Latvian [baĩdâs], Latvian [bîstas], Lithuanian_ST [BIJOTI], Polish [ˈbatɕ_ɕɛ̃], Russian [boˈjatʲsʲa], Serbocroatian [BOJATI_SE], Slovak [BAT_SA], Ukrainian [BOJATYSˈ]

**Cognate Set "2637":** French [kʀɛ̃dʀ], Provencal [CREGNE], Digor_Ossetic [tɐrs-], Persian [TARSIDAN]

**Cognate Set "122":** Bulgarian [strɐˈxuvɐm_sɛ], Slovenian [STRAH]

**Cognate Set "1030":** Hindi [DERNA], Urdu [ɖəɾnə]

- - 1. Basic Concept: "feather"

**Cognate Set "2783":** Bulgarian [pɛˈrɔ], Byelorussian [PERA], Czech [pɛːro], Latvian [spal̃va], Polish [ˈpʲurɔ], Russian [pʲeˈro], Serbocroatian [PERO], Slovak [PERO], Slovenian [PERU], Ukrainian [PERO], Hindi [PER], Kurdish [per], Persian [pær], Urdu [pəɾ]

***Cognate Set "967":** Portuguese_ST [ˈpenɐ], Armenian_Mod [T`EW], Armenian_Mod [pʰɛˈtuɾ], Danish [fjeɐ̯], Dutch_List [ver], English [ˈfɛðə], Faroese [ˈfjøːvʊr], Frisian [fɪ.ər], German [ˈfe:dɐ], Greek_Mod [fte̞ˈro̞], Icelandic_ST [fjœːðʏr], Norwegian [FJAER], Rumanian_List [pană], Sardinian_C [PINNA], Swedish [ˈfjɛ:dər], Albanian_Standard [pendë]

***Cognate Set "3052":** Breton_List [PLUN], Welsh_N [PLUEN], Catalan [ˈplomə], French [plym_(dwazo)], Italian [ˈpjuma], Portuguese_ST [PLUMA], Provencal [PLUMO], Spanish [ˈpluma]

- - 1. Basic Concept: "few"

**Cognate Set "973":** Dutch_List [ˈwɛinəχ], German [ˈve:nɪç]

**Cognate Set "944":** Irish_A [BEAGAN], Welsh_N [YCHYDIG]

**Cognate Set "795":** Slovak [NIEKOLKO], Serbocroatian [NEKOLIKO], Breton_List [NEBEUT]

**Cognate Set "555":** Hindi [THORA], Urdu [ˈt̪ʰo.ɽə]

**Cognate Set "2928":** English [fju:], Danish [fɔ], Faroese [ˈfɔajɪr], Icelandic_ST [FAIR], Norwegian [FA], Swedish [foː], Catalan [pɔk], French [pø], Italian [ˈpɔko], Portuguese_ST [ˈpo(ou)ku], Provencal [PAU], Sardinian_C [PAGUS], Spanish [ˈpoko]

**Cognate Set "2784":** Lithuanian_ST [NEDAUG], Persian [KAM]

**Cognate Set "123":** Bulgarian [ˈmalko], Czech [maːlo], Polish [ˈmawɔ], Russian [ˈmalo], Slovak [MALO], Slovenian [MOLO], Slovenian [NEKAJ]

- - 1. Basic Concept: "fight"

**Cognate Set "976":** Dutch_List [ˈvɛχtə(n)], English [faɪt]

**Cognate Set "713":** Latvian [kaûjas], Lithuanian_ST [KOVOTI]

**Cognate Set "316":** German [ˈkɛmp͜fn̩], Danish [ˈg̥ʰɛmb̥ə], Swedish [ˈcɛmpa]

**Cognate Set "2838":** Hindi [LERNA], Urdu [ləɽnə]

**Cognate Set "206":** Faroese [BERJAST], Serbocroatian [BORITI_SE]

**Cognate Set "1893":** Catalan [bətəˈʎa], Catalan [kumˈbatrə], French [sə_batʀ], Italian [komˈbattere], Portuguese_ST [COMBATER], Provencal [SE_BATRE], Rumanian_List [A_(SE)_BATE], Spanish [BATALLAR], Spanish [COMBATIR]

**Cognate Set "1890":** Czech [ZAPASITI], Slovak [ZAPASIT]

**Cognate Set "1889":** Pashto [DZANGEDEL], Persian [JANGIDAN_(DAˈVA_KARDAN)]

**Cognate Set "126":** Bulgarian [ˈbijɐ_sɛ], Byelorussian [BICCA], Czech [bojovat], Polish [ˈbʲitɕ_ɕɛ̃], Slovak [BIT_SA], Slovak [BOJOVAT], Ukrainian [BYTYSˈ]

**Cognate Set "1034":** Norwegian [SLASS], Faroese [SLAAST]

***Cognate Set "1892":** Rumanian_List [a_lupta], Albanian_Standard [luftoj], Portuguese_ST [luˈtaɾ]

- - 1. Basic Concept: "fingernail"

**Cognate Set "1035":** Albanian_Standard [thua], Catalan [ˈuŋɡlə], Czech [nɛɦɛt], Danish [nɑjl], Dutch_List [ˈnaɣəl], English [neɪl], French [ɔ̃gl], German [ˈna:gl̩], Greek_Mod [ˈniçi], Icelandic_ST [nœkl], Italian [ˈungja], Kurdish [neynûk], Latvian [nags], Lithuanian_ST [nagá], Persian [NĀXUN], Polish [paˈznɔkʲɛʨ], Provencal [ˈuŋɡlo], Rumanian_List [unghie], Russian [ˈnogotʲ], Serbocroatian [NOKAT], Spanish [ˈuɲa], Swedish [ˈnɑːɡɛl], Urdu [nɑχʊn], Welsh_N [EWIN]

- - 1. Basic Concept: "fire"

**Cognate Set "985":** Greek_Mod [fo̞ˈtça], Dutch_List [vyr], English [faɪə], Frisian [fjuər], German [ˈfɔyɐ], Armenian_Mod [huɾ]

**Cognate Set "803":** Serbocroatian [VATRA], Digor_Ossetic [art], Kurdish [agir], Pashto [OR], Persian [ATASH]

**Cognate Set "317":** Danish [il], Faroese [ELDUR], Icelandic_ST [ˈɛltʏr], Norwegian [ild], Swedish [ɛld]

**Cognate Set "2929":** Catalan [fɔk], French [fø], Italian [ˈfwɔko], Portuguese_ST [ˈfogu], Provencal [FIO], Provencal [ˈfɥɛʎo], Rumanian_List [fok], Sardinian_C [FOGU], Spanish [ˈfweɣo]

**Cognate Set "2839":** Hindi [AG], Urdu [ʔɑɡ], Bulgarian [ˈɔɡɤn], Byelorussian [VAGONˈ], Czech [oɦɛɲ], Latvian [uguns], Lithuanian_ST [‘ʊɡʲnʲɪs], Polish [ˈɔɡʲɛɲ], Russian [oˈgonʲ], Slovak [OHEN], Slovenian [OGEN], Ukrainian [VOGONˈ]

**Cognate Set "2540":** Norwegian [VARME], Albanian_Standard [zjarr]

**Cognate Set "1":** Breton_List [TAN], Irish_A [TEINE], Welsh_N [TAN]

- - 1. Basic Concept: "fish"

**Cognate Set "2785":** Hindi [MECHLI], Kurdish [masî], Persian [mɒːhiː], Urdu [məʧʰli]

**Cognate Set "2679":** Armenian_Mod [ʣuk], Latvian [zivs], Lithuanian_ST [ZUVIS]

**Cognate Set "129":** Bulgarian [ˈribɐ], Byelorussian [RYBA], Czech [rɪba], Polish [rˈɨba], Russian [ˈrɨba], Serbocroatian [RIBA], Slovak [RYBA], Slovenian [RIBA], Ukrainian [RYBA]

****Cognate Set "992":** Breton_List [PESK], Catalan [peʃ], Danish [fesg̥ʰ], Dutch_List [vɪs], English [fɪʃ], Faroese [FISKUR], French [pwasɔ̃], Frisian [fɪsk], German [fɪʃ], Icelandic_ST [ˈfɪskʏr], Irish_A [IASC], Italian [ˈpeʃʃe], Norwegian [FISK], Portuguese_ST [ˈpɐiʃɨ], Provencal [PEIS], Rumanian_List [ˈpeʃte], Sardinian_C [PISSI], Spanish [pesˈkaðo], Spanish [peθ], Swedish [fisk], Welsh_N [PYSGODYN], Albanian_Standard [peshk]

- - 1. Basic Concept: "five"

**Cognate Set "2543":** Albanian_Standard [pesë], Armenian_Mod [hing], Breton_List [PEMP], Bulgarian [pɛt], Byelorussian [PJACˈ], Catalan [siŋk], Czech [pjɛt], Danish [fεm], Digor_Ossetic [fondz], Dutch_List [vɛif], English [faɪv], Faroese [fɪmː], French [s_ɛ̃k], Frisian [fi:f], German [fʏnf], Greek_Mod [ˈpe̞(n)de̞], Hindi [pãtʃ], Icelandic_ST [fɪm], Irish_A [CUIG], Italian [ˈtʃinkwe], Latvian [pìeci], Lithuanian_ST [PENKI], Norwegian [FEM], Pashto [PINDZE], Persian [PANJ], Polish [pʲɛ̃tɕ], Portuguese_ST [ˈsĩku], Provencal [siŋk], Rumanian_List [ʧinʧʲ], Russian [pʲatʲ], Sardinian_C [CINKU], Serbocroatian [PET], Slovak [PAT], Slovenian [PJT], Spanish [ˈθiŋko], Swedish [fɛm], Ukrainian [PˈJATˈ], Urdu [pɑnʧ], Welsh_N [pɪmp]

- - 1. Basic Concept: "float"

**Cognate Set "686":** Italian [galledˈdʒare], Sardinian_C [GALLEGAI]

**Cognate Set "556":** Hindi [UTERANA], Urdu [t̪ɛɾnə]

**Cognate Set "2":** Breton_List [NEUI], Irish_A [SNAMH], Welsh_N [NOFIO]

****Cognate Set "227":** Armenian_Mod [LUAL], Armenian_Mod [loˈʁɑl], Bulgarian [ˈplavɐm], Byelorussian [PLAVACˈ], Czech [ploʊ̯t], Danish [ˈfly:ðə], English [fləʊt], Faroese [FLOTA], Greek_Mod [ˈple̞ˌo̞], Icelandic_ST [ˈfljouːta], Latvian [pludõ], Lithuanian_ST [PLAUKTI], Norwegian [FLYTE], Polish [ˈpwɨvaʨ], Russian [ˈplavatʲ], Serbocroatian [PLOVITI], Slovak [PLAVAT], Slovenian [PLAVAT], Swedish [ˈfly:ta], Ukrainian [PLAVATY], French [flɔte], Portuguese_ST [flutuˈaɾ], Provencal [FLOUTA], Rumanian_List [a_pluti], Spanish [floˈtaɾ]

- - 1. Basic Concept: "flow"

**Cognate Set "994":** Faroese [RENNA], Icelandic_ST [RENNA], Norwegian [RENNE], Swedish [ˈrina]

**Cognate Set "926":** Catalan [ˈkorə], Italian [ˈskorrere], Portuguese_ST [CORRER], Rumanian_List [a_curge]

**Cognate Set "847":** Bulgarian [tɛˈkɐ], Czech [tɛːtst], Latvian [tęk], Lithuanian_ST [TEKETI], Polish [tɕɛc], Russian [tʲetʃ], Serbocroatian [TECI], Ukrainian [TEKTY]

**Cognate Set "458":** Dutch_List [STROOMEN], Frisian [STROME], Swedish [ˈstrøma]

**Cognate Set "2931":** French [kule], Provencal [COULA]

**Cognate Set "2552":** Pashto [BAHEDEL], Hindi [BEHNA], Urdu [bəhnə]

**Cognate Set "1908":** Catalan [rəˈʒa], Provencal [RAJA]

**Cognate Set "1907":** Italian [fluˈire], Portuguese_ST [fluˈiɾ], Sardinian_C [KURRI], Spanish [fluˈiɾ]

**Cognate Set "1005":** Danish [ˈfly:ðə], Dutch_List [ˈvlujə(n)], English [fləʊ], German [ˈfli:sn̩], Swedish [ˈfly:ta], Byelorussian [PLYCˈ], Latvian [plûst], Polish [ˈpwɨnɔ̃ʨ], Slovak [PLYNUT], Slovenian [PLAVATI]

- - 1. Basic Concept: "flower"

**Cognate Set "2786":** Hindi [pʰul], Urdu [pʰul]

**Cognate Set "131":** Bulgarian [ˈt͡svɛtɛ], Byelorussian [KVETKA], Czech [kvjɛt], Polish [kfʲat], Russian [tsvʲet], Serbocroatian [CVET], Slovak [KVET], Slovenian [CVET], Ukrainian [KVITKA]

**Cognate Set "1013":** Breton_List [BLEUN], Irish_A [BLATH], Welsh_N [BLODEUYN], Catalan [flɔ], French [flœʀ], Italian [ˈfjore], Portuguese_ST [floɾ], Provencal [FLOUR], Rumanian_List [ˈflware], Sardinian_C [FRORI], Spanish [floɾ], Danish [b̥lʌmsd̥ʰ], Dutch_List [blum], English [flaʊə], Faroese [ˈblɔuma], Frisian [blom], German [ˈblu:mə], Icelandic_ST [plouːm], Norwegian [BLOMST], Swedish [ˈblɔmstər]

***Cognate Set "2542":** Greek_Mod [luˈluði], Albanian_Standard [lule]

****Cognate Set "2558":** Persian [GOL], Pashto [GUL]

- - 1. Basic Concept: "fly"

**Cognate Set "827":** Kurdish [firrîn], Pashto [ALVOTEL], Persian [PARVAZ_KARDAN]

**Cognate Set "459":** Armenian_Mod [tʰərˈʧʰɛl], Welsh_N [HEDEG], Greek_Mod [pe̞ˈto̞]

**Cognate Set "2933":** French [vɔle], Italian [voˈlare], Portuguese_ST [vuˈaɾ], Provencal [VOULA], Rumanian_List [a_zbura], Sardinian_C [BOLAI], Spanish [boˈlaɾ]

**Cognate Set "2840":** Hindi [ʊɽ.na:], Urdu [ʔʊɽnə]

**Cognate Set "132":** Bulgarian [lɛˈtjɐ], Byelorussian [LETACˈ], Czech [lɛːtat], Lithuanian_ST [LEKTI], Polish [ˈlataʨ], Polish [ˈletɕɛtɕ], Russian [lʲeˈtʲetʲ], Serbocroatian [LETETI], Slovak [LETET], Slovenian [LETETI], Ukrainian [LITATY]

**Cognate Set "1038":** Latvian [skrìen], Lithuanian_ST [‘sʲkʲɾʲɪsʲtʲɪ]

**Cognate Set "1022":** Danish [ˈfly:və], Dutch_List [ˈvliɣə(n)], English [flaɪ], Faroese [FLUGVA], German [ˈfli:gn̩], Icelandic_ST [ˈfljuːa], Norwegian [fɽyː], Swedish [ˈfly:ga]

- - 1. Basic Concept: "fog"

**Cognate Set "948":** Welsh_N [NIWL], Dutch_List [ˈnevəl], German [ˈne:bl̩], Italian [ˈnebbja], Portuguese_ST [nɨvuˈejɾu], Provencal [NEBLO], Rumanian_List [NEGURA], Sardinian_C [NEBBIA], Spanish [ˈnjeβla]

**Cognate Set "5":** Catalan [ˈbromə], Breton_List [BRUMENN]

**Cognate Set "318":** Danish [d̥ʰɔ:wə], Faroese [ˈtoːka], Icelandic_ST [ˈθɔːka], Norwegian [TAKE], Swedish [TJOKA]

**Cognate Set "2565":** Dutch_List [mɪst], Albanian_Standard [mjegull], Armenian_Mod [məˈʃuʃ], Bulgarian [mɤˈɡla], Byelorussian [IMHLA], Czech [ml̩ɦa], Digor_Ossetic [meʁɐ], Greek_Mod [o̞ˈmixli], Latvian [migla], Lithuanian_ST [MIGLA], Pashto [MIH], Persian [MEH], Polish [mɡwa], Serbocroatian [MAGLA], Slovak [HMLA], Slovenian [MEGLA], Ukrainian [MRJAKA]

**Cognate Set "1041":** Byelorussian [TUMAN], Russian [tuˈman], Ukrainian [TUMAN]

- - 1. Basic Concept: "foot"

**Cognate Set "714":** Latvian [kãja], Lithuanian_ST [KOJA]

**Cognate Set "6":** Breton_List [TROAD], Irish_A [TROIGH], Welsh_N [trɔid]

**Cognate Set "254":** Czech [noɦa], Russian [noˈga], Serbocroatian [NOGA], Slovak [NOHA], Ukrainian [NOGA]

**Cognate Set "228":** Bulgarian [stɤˈpalo], Byelorussian [STAPA], Polish [ˈstɔpa], Slovenian [STAPOLU]

**Cognate Set "1031":** Armenian_Mod [votkʰ], Catalan [pɛuw], Danish [foð], Dutch_List [vut], English [fʊt], Faroese [ˈfɔutʊr], French [pje], Frisian [fuət], German [fu:s], Greek_Mod [ˈpo̞ði], Hindi [PER], Icelandic_ST [ˈfouːtʏr], Italian [ˈpjɛde], Kurdish [pê], Norwegian [fot], Persian [pɒ], Portuguese_ST [pɛ], Provencal [pɛ], Rumanian_List [piˈʧjor], Sardinian_C [PEI], Spanish [pje], Swedish [fuːt], Urdu [pɑũ]

- - 1. Basic Concept: "four"

**Cognate Set "2583":** Albanian_Standard [katër], Armenian_Mod [ʧʰoɾs], Breton_List [PEVAR_(M)], Bulgarian [ˈt͡ʃɛtiri], Byelorussian [CATYRY], Catalan [ˈkwatɾə], Czech [tʂtɪr̝ɪ], Danish [ˈfi:ʌ], Digor_Ossetic [cuppar], Dutch_List [vir], English [fɔ:], Faroese [ˈfʊiɹa], French [katʀ], Frisian [ˈfjo.uər], German [fi:ɐ̯], Greek_Mod [ˈte̞se̞ˌra], Hindi [CAR], Icelandic_ST [FJORIR], Irish_A [CEATHAIR], Italian [ˈkwattro], Latvian [četri], Lithuanian_ST [KETURI], Norwegian [FIRE], Pashto [CALOR], Persian [CHAHAR_(CHAR)], Polish [ˈʧtɛrɨ], Portuguese_ST [ˈkuatɾu], Provencal [ˈkatre], Rumanian_List [ˈpatru], Russian [tʃeˈtɨrʲe], Sardinian_C [KWATTRU], Serbocroatian [CETIRI], Slovak [STYRI], Slovenian [STJRI], Spanish [ˈkwatɾo], Swedish [ˈfyːra], Ukrainian [COTYRY], Urdu [ʧɑɾ], Welsh_N [PEDWAR]

- - 1. Basic Concept: "freeze"

**Cognate Set "715":** Latvian [sal̂st], Lithuanian_ST [SALDYTI]

**Cognate Set "2934":** Catalan [ʒəˈla], French [ʒəle], Italian [dʒeˈlare], Portuguese_ST [ʒɨˈlaɾ], Provencal [GELA], Rumanian_List [a_îngheța], Sardinian_C [GELAI], Spanish [eˈlaɾ]

**Cognate Set "2841":** Hindi [JEMNA], Urdu [ʤəmnə]

**Cognate Set "2591":** Pashto [JAX_KEDEL], Persian [YAKH_BASTAN]

**Cognate Set "2547":** Bulgarian [zɐˈmrɤ̞zvɐm], Byelorussian [ZJAMARATYVACˈ], Czech [zamr̩znoʊ̯t], Polish [zaˈmarzaʨ], Russian [zamʲerˈzatʲ], Slovak [MRZNUT], Slovenian [ZMRZNE], Ukrainian [MERZNUTY]

**Cognate Set "1037":** Breton_List [REVI], Irish_A [REODHADH], Welsh_N [RHEWI], Danish [ˈfʁy:sə], Dutch_List [ˈvrizə(n)], English [fri:z], Faroese [FRYSTA], Frisian [FRIEZE], German [ˈfri:rən], Icelandic_ST [ˈfrjouːsa], Norwegian [FRYSE], Swedish [ˈfry:sa]

- - 1. Basic Concept: "fruit"

**Cognate Set "2842":** Hindi [pʰəl], Urdu [pʰəl]

**Cognate Set "2599":** Pashto [MEVA], Persian [miːve]

**Cognate Set "1904":** Bulgarian [plɔt], Byelorussian [PLOD], Czech [plot], Russian [plod], Ukrainian [PLID]

***Cognate Set "134":** German [opst], Czech [OVOCE], Polish [ˈɔvɔʦ], Serbocroatian [VOCE], Slovak [OVOCIE], Ukrainian [OVOC]

****Cognate Set "1043":** Catalan [fruˈi], French [fʀɥi], Italian [ˈfrutto], Portuguese_ST [ˈfɾutɐ], Provencal [FRU], Rumanian_List [fruct], Sardinian_C [FRUTTA], Spanish [ˈfɾuta], Albanian_Standard [frut], Breton_List [FROUEZ], Danish [fʁɔg̥d̥ʰ], Dutch_List [vrʏχt], English [fru:t], Frisian [frøxt], German [fruxt], Greek_Mod [ˈfruto̞], Swedish [frɵkt], Welsh_N [FFRWYTH]

- - 1. Basic Concept: "full"

**Cognate Set "1009":** Albanian_Standard [plot], Armenian_Mod [li], Czech [pl̩niː], Dutch_List [vol], English [full], French [plɛ̃], German [fɔl], German [voll], Hindi [pu:ɾa:], Italian [ˈpjɛno], Kurdish [pirr], Latvian [pil_̃ns], Lithuanian_ST [pilnas], Norwegian [fʉl], Polish [ˈpɛwnɨ], Rumanian_List [plin], Russian [ˈpolnɨj], Spanish [ˈʎeno], Swedish [fɵl], Urdu [puɾə]

- - 1. Basic Concept: "give"

**Cognate Set "8":** Breton_List [REI], Welsh_N [RHODDI]

**Cognate Set "2548":** Armenian_Mod [tɑl], Bulgarian [ˈdavɐm], Byelorussian [DAVACˈ], Catalan [duˈna], Czech [daːvat], Digor_Ossetic [dɐtt-], French [dɔne], Greek_Mod [ˈðino̞], Hindi [d̪ena], Italian [ˈdare], Kurdish [dan], Latvian [dôd], Lithuanian_ST [‘duɔtʲɪ], Persian [DADAN], Polish [ˈdavaʨ], Portuguese_ST [daɾ], Provencal [DOUNA], Rumanian_List [a_da], Russian [daˈvatʲ], Sardinian_C [DONAI], Serbocroatian [DATI], Slovak [DAT], Slovenian [DATI], Spanish [daɾ], Ukrainian [DAVATY], Urdu [d̪enə]

****Cognate Set "1049":** Danish [g̥i], Dutch_List [ˈɣevə(n)], Faroese [GEVA], German [ˈge:bn̩], Icelandic_ST [ˈcɛːva], Norwegian [GI], Swedish [je:], English [gɪv]

- - 1. Basic Concept: "good"

**Cognate Set "9":** Breton_List [MAT], Irish_A [MAITH]

**Cognate Set "2935":** Catalan [bɔ], French [bɔ̃], Italian [ˈbwɔno], Portuguese_ST [bõ], Provencal [BON], Rumanian_List [bun], Sardinian_C [BONU], Spanish [ˈbweno]

**Cognate Set "2765":** Armenian_Mod [lɑv], Latvian [labs]

**Cognate Set "135":** Bulgarian [doˈbɤ̞r], Byelorussian [DOBRY], Czech [dobriː], Polish [ˈdɔbrɨ], Serbocroatian [DOBAR], Slovak [DOBRY], Slovenian [DOBRO], Ukrainian [DOBRYJ]

**Cognate Set "1055":** Danish [g̥o], Dutch_List [ɣut], English [gʊd], Faroese [GODUR], German [gu:t], Icelandic_ST [ˈkouːðʏr], Norwegian [GOD], Swedish [gu:d]

**Cognate Set "1046":** Hindi [ə:t:ʃʰa:], Urdu [ʔəʧʰɑ]

- - 1. Basic Concept: "grass"

**Cognate Set "534":** Latvian [zâle], Lithuanian_ST [ʒo’lʲe:]

**Cognate Set "2936":** Catalan [ˈerßə], French [ɛʀb], Italian [ˈɛrba], Portuguese_ST [RELVA], Portuguese_ST [ˈɛɾvɐ], Provencal [GERME], Provencal [ˈɛrβo], Rumanian_List [ˈjarbə], Sardinian_C [ERBA], Spanish [ˈjeɾβa]

**Cognate Set "2845":** Hindi [GHAS], Urdu [ɡʰɑs]

**Cognate Set "136":** Bulgarian [trɛˈva], Byelorussian [TRAVA], Czech [traːva], Polish [ˈtrava], Russian [traˈva], Serbocroatian [TRAVA], Slovak [TRAVA], Slovenian [TRAVA], Ukrainian [TRAVA]

**Cognate Set "1064":** Danish [g̥ʁas], Dutch_List [ɣrɑs], English [grɑ:s], Faroese [ɡras], Frisian [gɛːs], German [gra:s], Icelandic_ST [GRAS], Norwegian [GRESS], Swedish [grɛ:s]

- - 1. Basic Concept: "green"

**Cognate Set "588":** Hindi [HERA], Urdu [həɾɑ]

**Cognate Set "2937":** Breton_List [GWER], Welsh_N [ɡwɨrð], Catalan [bert], French [vɛʀ], Italian [ˈverde], Portuguese_ST [ˈveɾdɨ], Provencal [VERT], Rumanian_List [ˈverde], Sardinian_C [BIRDI], Spanish [ˈbeɾðe]

**Cognate Set "2618":** Breton_List [GLAS], Irish_A [GLAS], Kurdish [hêşîn], Pashto [SIN], Bulgarian [zɛˈlɛn], Byelorussian [ZJALENY], Czech [zɛlɛniː], Latvian [zal_̜š], Lithuanian_ST [ZALIAS], Polish [ʑɛˈlɔnɨ], Russian [zʲeˈlʲonɨj], Serbocroatian [ZELEN], Slovenian [ZELENO], Ukrainian [ZELENYJ]

**Cognate Set "1071":** Danish [g̥ʁɶ̝n], Dutch_List [ɣrun], English [gri:n], Faroese [GRONUR], Frisian [gri.ən], German [gry:n], Icelandic_ST [kraitn], Norwegian [ɡɾøn], Swedish [grøːn]

- - 1. Basic Concept: "guts"

**Cognate Set "545":** Greek_Mod [ANDERA], Serbocroatian [UTROBA], Hindi [AT], Urdu [bəɽi_ʔɑ̃t̪], Italian [intesˈtino], Portuguese_ST [ẽˈtɾɐɲɐʃ], Spanish [INTESTINO]

**Cognate Set "286":** Czech [vɲɪtr̝̊noscɪ], Polish [vnɛ̃tʃˈnɔɕʨi], Slovak [VNUTORNOSTI]

**Cognate Set "2685":** Polish [jɛˈʎita], Armenian_Mod [ɑˈʁikʰ]

**Cognate Set "265":** Catalan [buˈðeʎs], French [bwajo], Provencal [BUDEU]

**Cognate Set "229":** Byelorussian [KISKI], Polish [ˈkʲiʃkʲi], Russian [ˈkɨskɨ], Ukrainian [KISKI]

**Cognate Set "1915":** Albanian_Standard [zorrë], Faroese [GARNAR], Latvian [xar̂na], Lithuanian_ST [ZARNOS]

**Cognate Set "137":** Bulgarian [t͡ʃɛˈrva], Slovenian [CEJVA]

**Cognate Set "1078":** Danish [d̥ʰɑm], Dutch_List [dɑrm], Faroese [TARMAR], German [gəˈdɛrm], Icelandic_ST [THARMAR], Norwegian [TARMER], Swedish [tarm]

- - 1. Basic Concept: "hair"

**Cognate Set "831":** Kurdish [mû], Persian [mu]

**Cognate Set "2938":** Catalan [kəˈßɛʎ], French [ʃəvø], Italian [kaˈpello], Portuguese_ST [kɐˈβelu], Provencal [CABEU], Spanish [kaˈβeʎo]

**Cognate Set "2846":** Hindi [BAL], Urdu [bɑl]

**Cognate Set "2241":** Catalan [pɛl], Italian [ˈpelo], Provencal [PEU], Rumanian_List [păr], Sardinian_C [PILU], Spanish [PELO]

**Cognate Set "207":** Welsh_N [GWALLT], Pashto [VESTE], Byelorussian [VOLAS], Czech [vlasɪ], Polish [ˈvwɔsɨ], Russian [ˈvolosɨ], Slovak [VLAS], Slovenian [LASJE], Ukrainian [VOLOSSJA]

**Cognate Set "139":** Bulgarian [koˈsa], Serbocroatian [KOSA]

**Cognate Set "1085":** Danish [hɒ], Dutch_List [har], English [hɛə], Faroese [HAR], Frisian [hi.ər], German [ha:ɐ̯], Icelandic_ST [hauːr], Norwegian [HAR], Swedish [ho:r]

- - 1. Basic Concept: "hand"

**Cognate Set "632":** Irish_A [LAMH], Welsh_N [LLAW]

**Cognate Set "2939":** Catalan [ma], French [mɛ̃], Italian [ˈmano], Portuguese_ST [mɐ̃u], Provencal [ma], Rumanian_List [ˈmɨ.nə], Sardinian_C [MANU], Spanish [ˈmano]

**Cognate Set "2788":** Hindi [ɦaːt̪ʰ], Kurdish [dest], Pashto [LAS], Persian [dæstʰ], Urdu [hɑt̪ʰ]

**Cognate Set "2553":** Armenian_Mod [ʣɛrkʰ], Greek_Mod [ˈçe̞ri], Albanian_Standard [dorë]

**Cognate Set "140":** Bulgarian [rɤˈka], Byelorussian [RUKA], Czech [rʊka], Latvian [ròka], Lithuanian_ST [ɾɐŋ’kɐ], Polish [ˈrɛ̃ka], Russian [ruˈka], Serbocroatian [RUKA], Slovak [RUKA], Slovenian [RAKA], Ukrainian [RUKA]

**Cognate Set "1090":** Danish [hʌn], Dutch_List [hɑnt], English [hænd], Faroese [hɔnd], Frisian [hɔ:n], German [hant], Icelandic_ST [hœnt], Norwegian [hånd], Swedish [hand]

- - 1. Basic Concept: "he"

**Cognate Set "589":** Hindi [YEH], Hindi [vo], Urdu [woʰ]

**Cognate Set "2940":** Catalan [eʎ], French [il], Italian [ˈeʎʎi], Portuguese_ST [ˈelɨ], Provencal [EU], Rumanian_List [jel], Spanish [el]

**Cognate Set "1923":** Armenian_Mod [nɑ], Byelorussian [EN], Czech [on], Polish [ɔn], Russian [on], Serbocroatian [ON], Slovak [ON], Slovenian [ON], Ukrainian [VIN]

**Cognate Set "1921":** Bulgarian [tɔj], Albanian_Standard [ai], Breton_List [HEN], Greek_Mod [TOS], Greek_Mod [afˈto̞s], Irish_A [SE], Sardinian_C [ISSU]

**Cognate Set "1920":** Welsh_N [ɛv], Lithuanian_ST [jɪs], Frisian [ər], German [e:ɐ̯]

**Cognate Set "1098":** Danish [han], Dutch_List [hɛi], English [hi:], Faroese [HANN], Frisian [hɛi], Icelandic_ST [hanː], Norwegian [HAN], Swedish [han]

- - 1. Basic Concept: "head"

**Cognate Set "2646":** Digor_Ossetic [sɐr], Hindi [SIR], Kurdish [sar], Pashto [SAR], Persian [sær], Urdu [səɾ]

**Cognate Set "2249":** French [tɛt], Italian [ˈtɛsta], Provencal [TESTO]

**Cognate Set "141":** Armenian_Mod [gəˈluχ], Bulgarian [ɡlɐˈva], Byelorussian [GALAVA], Czech [ɦlava], Latvian [gal_̂va], Lithuanian_ST [GALVA], Polish [ˈɡwɔva], Russian [goloˈva], Serbocroatian [GLAVA], Slovak [HLAVA], Slovenian [GLAVA], Ukrainian [GOLOVA]

**Cognate Set "13":** Breton_List [PENN], Irish_A [CEANN], Welsh_N [PEN]

**Cognate Set "1104":** Catalan [kap], Portuguese_ST [kɐˈβesɐ], Provencal [kap], Rumanian_List [kap], Spanish [kaˈβeθa], Danish [ho:əð], Dutch_List [hoft], English [hɛd], Faroese [høːvʊr], Faroese [hœdː], Frisian [HAED], Frisian [kɔp], Icelandic_ST [HOFUO], Norwegian [HODE], Swedish [ˈhʉ:vɵd]

- - 1. Basic Concept: "hear"

**Cognate Set "716":** Latvian [dzìrd], Lithuanian_ST [GIRDE_TI]

**Cognate Set "2942":** Italian [uˈdire], Portuguese_ST [oˈviɾ], Provencal [AUSI], Rumanian_List [a_auzi], Spanish [oˈiɾ]

**Cognate Set "2687":** Armenian_Mod [ləˈsɛl], Breton_List [KLEVOUT], Bulgarian [ˈt͡ʃuvɐm], Byelorussian [CUCˈ], Czech [slɪʃɛt], Hindi [SUNNA], Irish_A [CLOS], Persian [SHENIDAN], Polish [ˈswɨʃɛʨ], Russian [ˈslɨʃatʲ], Serbocroatian [CUTI], Slovak [CUT], Slovenian [CUJES], Ukrainian [SLUXATY], Urdu [sʊnnə], Welsh_N [CLYWED]

**Cognate Set "1111":** Greek_Mod [aˈkuˌo̞], Danish [ˈho:ʌ], Dutch_List [ˈhorə(n)], English [hɪə], Faroese [HOYRA], Frisian [ˈjɛrə], German [ˈhørən], Icelandic_ST [HEYRA], Norwegian [HORE], Swedish [ˈhœ:ra]

**Cognate Set "1053":** Sardinian_C [INTENDI], French [ɑ̃tɑ̃dʀ], Provencal [ENTENDRE]

**Cognate Set "1052":** Catalan [sənˈti], Italian [senˈtire]

- - 1. Basic Concept: "heart"

**Cognate Set "2656":** Armenian_Mod [siɾt], Bulgarian [sɤˈrt͡sɛ], Byelorussian [SERCA], Catalan [kɔɾ], Czech [sr̩tsɛ], Danish [ˈjɛɐ̯d̥ʰə], Digor_Ossetic [zɐrdɐ], Dutch_List [hɑrt], English [hɑ:t], Faroese [ˈjar̥ta], French [kœʀ], Frisian [həːt], German [hɛrt͜s], Greek_Mod [karˈðʝa], Hindi [hṛday], Icelandic_ST [ˈçar̥ta], Irish_A [CROIDHE], Italian [ˈkwɔre], Latvian [siȓds], Lithuanian_ST [ʃʲɪrʲˈdʲɪs], Norwegian [hjerte], Pashto [ZRE], Polish [ˈsɛrʦɛ], Portuguese_ST [kuɾɐˈsɐ̃w], Provencal [kɔr], Russian [ˈsʲerdtse], Sardinian_C [KORI], Serbocroatian [SRCE], Slovak [SRDCE], Slovenian [SRCJ], Spanish [koɾaˈθon], Swedish [ˈjæʈa], Ukrainian [SERCE]

**Cognate Set "14":** Breton_List [KALON], Welsh_N [CALON]

****Cognate Set "2790":** Hindi [DIL], Urdu [d̪ɪl], Kurdish [dil]

- - 1. Basic Concept: "heavy"

**Cognate Set "807":** Digor_Ossetic [wɐzzaw], Persian [SANGIN]

**Cognate Set "633":** Irish_A [TROM], Welsh_N [TRWM]

**Cognate Set "2847":** Catalan [pəˈzan], Italian [peˈsante], Portuguese_ST [pɨˈzaðu], Spanish [peˈsaðo]

**Cognate Set "1929":** French [luʀ], Provencal [LOURD]

**Cognate Set "1927":** Greek_Mod [vaˈris], Hindi [BHARI], Urdu [ˈbʰɑ.ɾi], Italian [ˈgrɛve], Provencal [EVO], Provencal [GREU], Rumanian_List [greu], Sardinian_C [GRAI], Spanish [GRAVE]

**Cognate Set "15":** Frisian [POUNICH], Breton_List [POUNNER]

**Cognate Set "142":** Danish [d̥ʰɔŋ], Icelandic_ST [ˈθuŋkʏr], Norwegian [TUNG], Bulgarian [ˈtɛʒɤk], Byelorussian [CJAZKI], Czech [cɛʃkiː], Polish [ˈʨɛ̃ʃki], Russian [tʲaˈʒolɨj], Serbocroatian [TEZAK], Slovak [TAZKY], Slovenian [TEZKO], Ukrainian [TJAZKYJ]

**Cognate Set "1120":** Dutch_List [zwar], Frisian [svi.ər], German [ʃve:ɐ̯], Swedish [stur], Swedish [svo:r]

- - 1. Basic Concept: "here"

**Cognate Set "590":** Hindi [YEHA], Urdu [jəhɑ̃], Catalan [əˈki], French [isi], Italian [kwi], Portuguese_ST [ɐˈki], Provencal [EICI], Rumanian_List [INCOACE], Rumanian_List [aˈitʃʲ], Spanish [aˈki], Armenian_Mod [ɑɪ̯sˈtɛʁ]

**Cognate Set "1936":** Catalan [əˈki], French [isi], Italian [kwi], Portuguese_ST [ɐˈki], Provencal [EICI], Rumanian_List [INCOACE], Rumanian_List [aˈitʃʲ], Spanish [aˈki]

**Cognate Set "1934":** Czech [zdɛ], Russian [zdʲesʲ]

**Cognate Set "1932":** Hindi [YEHA], Urdu [jəhɑ̃]

**Cognate Set "1930":** Irish_A [ANNSO], Albanian_Standard [këtu], Bulgarian [tuk], Byelorussian [TUT], Lithuanian_ST [tʃʲɛ], Polish [ˈtu(taj)], Slovak [TU], Slovenian [TUKAJ], Ukrainian [TUT]

**Cognate Set "16":** Breton_List [AMAN], Welsh_N [YMA]

**Cognate Set "1126":** Armenian_Mod [ɑɪ̯sˈtɛʁ], Catalan [əˈki], Danish [hεɐ̯], Dutch_List [hir], English [hɪə], Faroese [HER], French [isi], Frisian [jɪr], German [hi:ɐ̯], Icelandic_ST [hjɛr], Italian [kwi], Latvian [šeĩt], Norwegian [HER], Portuguese_ST [ɐˈki], Provencal [EICI], Rumanian_List [INCOACE], Rumanian_List [aˈitʃʲ], Spanish [aˈki], Swedish [hɛːr]

- - 1. Basic Concept: "hit"

**Cognate Set "2943":** Catalan [kupəˈʒa], Italian [kolˈpire], Spanish [ɡolpeˈaɾ]

**Cognate Set "1947":** Provencal [PICA], Sardinian_C [PIGAI]

**Cognate Set "1946":** Catalan [ˈbatɾə], Italian [ˈbattere]

**Cognate Set "1943":** Faroese [SLAA], Frisian [SLAEN], German [ˈʃla:gŋ̩], Icelandic_ST [stlauː], Norwegian [SLA], Swedish [slo:]

**Cognate Set "1941":** Polish [bʲitɕ], Russian [bitʲ]

**Cognate Set "143":** Bulgarian [ˈudrjɐm], Byelorussian [UDARACˈ], Czech [ʊdɛr̝ɪt], Polish [uˈdɛ̃ʒaʨ], Serbocroatian [UDARITI], Slovak [UDERIT], Slovenian [UDARIT], Ukrainian [UDARJATY]

***Cognate Set "1944":** Danish [ˈd̥ʰʁafə], German [ˈtrɛfn̩]

- - 1. Basic Concept: "hold"

**Cognate Set "2944":** Catalan [təˈni], French [təniʀ], Italian [teˈnere], Portuguese_ST [ter], Provencal [TENI], Rumanian_List [a_ˈtsine], Sardinian_C [TENNI], Spanish [teˈneɾ]

**Cognate Set "2848":** Pashto [LAREL], Hindi [DHERNA], Bulgarian [dɤ̞rˈʒɐ], Czech [dr̩ʒɛt], Russian [dʲerˈzatʲ], Serbocroatian [DRZATI], Slovak [DRZAT], Slovenian [DRZAT_U_RAKI]

**Cognate Set "230":** Byelorussian [TPYMACˈ], Polish [ˈtʃɨmaʨ], Ukrainian [TRYMATY]

**Cognate Set "19":** Breton_List [DERCˈHEL], Welsh_N [DAL]

**Cognate Set "1353":** Danish [ˈhʌlə], Dutch_List [ˈhɑudə(n)], English [həʊld], Faroese [HALDA], Frisian [hɔːdə], German [ˈhaltn̩], Icelandic_ST [HALDA], Norwegian [HOLDE], Swedish [ˈhɔla]

- - 1. Basic Concept: "horn"

**Cognate Set "1777":** Bulgarian [rog], Czech [rox], Latvian [rags], Lithuanian_ST [ragas], Polish [ruk], Russian [ˈrog], Serbocroatian [rog]

****Cognate Set "1776":** Danish [hoɐ̯n], Dutch_List [horn], English [horn], French [kɔʀn], German [hɔrn], Greek_Mod [ˈce̞raˌto̞], Hindi [sĩːɡ], Icelandic_ST [hɔrtn], Italian [ˈkɔrno], Rumanian_List [korn], Spanish [ˈkweɾno], Swedish [hu:ɳ], Urdu [siŋɡ], Breton_List [korn], Welsh_N [corn]

- - 1. Basic Concept: "how"

**Cognate Set "232":** Byelorussian [JAK], Czech [jak], Polish [jak], Slovak [AKO], Ukrainian [JAK]

**Cognate Set "1528":** Albanian_Standard [si], Breton_List [PENAOS], Bulgarian [kak], Catalan [kɔm], Danish [vɒˈle:ðəs], Digor_Ossetic [kud], Dutch_List [hu], English [haʊ], Faroese [HVUSSUR], French [kɔmɑ̃], Frisian [HOˈT], German [vi:], Greek_Mod [po̞s], Hindi [KESA], Icelandic_ST [HVERNIG], Irish_A [CONAS], Italian [ˈkome], Latvian [kâ], Lithuanian_ST [KAIP], Norwegian [HVORLEDES], Pashto [CENGA], Persian [tʃʰetou̯ɾ], Portuguese_ST [ˈkomu], Provencal [COUME], Rumanian_List [kum], Russian [kak], Sardinian_C [KOMMENTI], Serbocroatian [KAKO], Slovenian [KAKO], Spanish [ˈkomo], Swedish [ˈhʉ:rɵ], Urdu [kɛse]

- - 1. Basic Concept: "hunt"

**Cognate Set "718":** Latvian [medĩ], Lithuanian_ST [mʲɛˈdʲʒʲotʲɪ]

**Cognate Set "635":** Irish_A [SEILG], Welsh_N [HELA]

**Cognate Set "368":** Faroese [VEIDA_(VEIDA)], Icelandic_ST [VEIOA]

**Cognate Set "2945":** Breton_List [KAS_KUIT], Catalan [kəˈsa], French [ʃase], Italian [katˈtʃare], Portuguese_ST [kɐˈsaɾ], Provencal [kaˈsa], Sardinian_C [KASSAI], Spanish [kaˈθaɾ]

**Cognate Set "144":** Bulgarian [loˈvuvɐm], Byelorussian [NALJARACˈ], Czech [lovɪt], Polish [pɔˈlɔvaʨ], Serbocroatian [LOVITI], Ukrainian [POLJUVATY]

***Cognate Set "1767":** Slovenian [JAGAT], Dutch_List [ˈjaɣə(n)], Frisian [JEIJE], German [ˈja:gn̩], Norwegian [GA_PA_JAKT], Danish [ˈjɛ:jə], Swedish [ˈjɑːɡa]

****Cognate Set "2700":** Pashto [SKAR_KAVEL], Persian [SHEKAR_KARDAN], Hindi [SIKAR_+_KERNA], Urdu [ʃɪkɑɾ_kəɾnə]

- - 1. Basic Concept: "husband"

**Cognate Set "559":** Latvian [vĩrs], Lithuanian_ST [‘ʋʲi:rɐs], Irish_A [FEAR], Welsh_N [gu:r]

**Cognate Set "2793":** Catalan [məˈɾit], French [maʀi], Italian [maˈrito], Portuguese_ST [mɐˈɾiðu], Provencal [MARIT], Sardinian_C [MARIRU], Spanish [maˈɾiðo]

**Cognate Set "1960":** Dutch_List [ˈɛχtχənot], German [ˈe:əman]

**Cognate Set "1957":** Catalan [ˈɔmə], Provencal [OME]

**Cognate Set "1956":** Catalan [əsˈpɔs], Italian [ˈspozo], Portuguese_ST [ɨʃˈpozu], Spanish [esˈposo]

**Cognate Set "1773":** Danish [man], Faroese [ˈmɛavʊr], Frisian [mɔn], German [ˈe:əman], Icelandic_ST [ˈeiːjɪn.maːðʏr], Swedish [man], Czech [manʒɛl], Polish [mɔ̃ʒ], Russian [muʒ], Serbocroatian [MUZ], Slovak [MANZEL], Slovenian [MAS], Ukrainian [MUZ]

***Cognate Set "1962":** English [hʌzbənd], Faroese [ˈhʏsˌbœndɪ], Swedish [HUSHALLA]

- - 1. Basic Concept: "ice"

**Cognate Set "2946":** Catalan [ʒɛl], French [glas], Italian [ˈgjattʃo], Portuguese_ST [ˈʒelu], Provencal [GLACO], Rumanian_List [gheață], Sardinian_C [GIACCU], Spanish [ˈjelo]

**Cognate Set "2849":** Hindi [BERPH], Urdu [bəɾf]

**Cognate Set "1963":** Irish_A [LEAC_OIDHRE], Welsh_N [IA], Pashto [JAX], Persian [YAKH]

**Cognate Set "1778":** Digor_Ossetic [jeχ], Danish [is], Dutch_List [ɛis], English [aɪs], Faroese [ˈʊisʊr], Frisian [iːs], German [a‿is], Icelandic_ST [i:s], Norwegian [IS], Swedish [i:s]

**Cognate Set "145":** Bulgarian [lɛt], Byelorussian [LED], Czech [lɛt], Latvian [lędus], Lithuanian_ST [LEDAS], Polish [lut], Russian [lʲod], Serbocroatian [LED], Slovak [L_AD], Slovenian [LETT], Ukrainian [LID]

- - 1. Basic Concept: "if"

**Cognate Set "319":** Danish [ves], Faroese [VISS], Norwegian [HVIS]

**Cognate Set "2947":** Catalan [si], French [si], Italian [se], Portuguese_ST [sɨ], Provencal [SE], Sardinian_C [SI], Spanish [si]

**Cognate Set "287":** Russian [ˈjesli], Czech [jɛstlɪ], Polish [jɛˈʒɛʎɨ]

**Cognate Set "2794":** Hindi [EGER], Persian [AGAR], Urdu [ʔəɡəɾ]

**Cognate Set "2737":** Digor_Ossetic [kɐd/ku], Pashto [KA]

**Cognate Set "21":** Breton_List [MA], Irish_A [MA]

**Cognate Set "1971":** Faroese [ʊmː], Swedish [ɔm]

**Cognate Set "1970":** German [vɛn], Slovak [KED], Lithuanian_ST [KAD]

**Cognate Set "1968":** Byelorussian [KALI], Ukrainian [KOLY]

**Cognate Set "1967":** Latvian [ja], Lithuanian_ST [jæj]

**Cognate Set "1966":** English [ɪf], Frisian [ɔf], Icelandic_ST [EF], Bulgarian [ɐˈkɔ], Serbocroatian [AKO], Slovak [AK], Ukrainian [JAKSCO]

- - 1. Basic Concept: "in"

**Cognate Set "1982":** Catalan [a], Dutch_List [vɑn]

**Cognate Set "1980":** Catalan [dins], French [dɑ̃], Provencal [DINS]

**Cognate Set "1978":** Serbocroatian [U], Byelorussian [U], Ukrainian [U]

**Cognate Set "1976":** Greek_Mod [se̞], Hindi [mẽ], Urdu [mẽ]

**Cognate Set "1974":** Albanian_Standard [në], Breton_List [E], Bulgarian [v/f], Catalan [dins], Czech [v], Danish [i], Dutch_List [ɪn], English [ɪn], Faroese [I], French [dɑ̃], Frisian [ˈbɪnən], German [ɪn], Hindi [ENDER], Icelandic_ST [I], Irish_A [I], Italian [in], Latvian [iekš], Lithuanian_ST [i:], Norwegian [I], Polish [v], Portuguese_ST [ɐ̃j], Provencal [DINS], Provencal [EN], Rumanian_List [ɨn], Russian [v], Sardinian_C [IN], Slovak [V], Spanish [en], Swedish [iː], Ukrainian [V], Urdu [ʔənd̪əɾ], Welsh_N [YN]

**Cognate Set "1825":** Catalan [də]

- - 1. Basic Concept: "kill"

**Cognate Set "394":** French [tɥe], Provencal [TUA]

**Cognate Set "2948":** Catalan [məˈta], Portuguese_ST [mɐˈtaɾ], Spanish [maˈtaɾ]

**Cognate Set "2795":** Kurdish [kuştin], Persian [KOSHTAN]

**Cognate Set "22":** Breton_List [LAZA], Welsh_N [LLADD]

**Cognate Set "1793":** Danish [ˈdʁε:bə], Faroese [DREPA], Icelandic_ST [ˈtrɛːpa], Norwegian [DREPE], Swedish [ˈdrɛ:pa]

**Cognate Set "146":** Bulgarian [oˈbivɐm], Byelorussian [ZABIVACˈ], Czech [zabiːjɛt], Polish [ˈzabʲiʨ], Russian [ubiˈvatʲ], Serbocroatian [UBITI], Slovak [ZABIT], Slovenian [UBIJAT], Ukrainian [UBYVATY]

**Cognate Set "1061":** Dutch_List [ˈdodə(n)], Frisian [DEIJE], Frisian [ˈdɪ.ədzjə], German [ˈtø:tn̩], Swedish [døːda]

**Cognate Set "1057":** Italian [utˈtʃidere], Rumanian_List [a_uˈtʃide]

***Cognate Set "2851":** Rumanian_List [a_omoˈrɨ], Irish_A [MARBHUGHADH], Digor_Ossetic [mar-], Hindi [marna], Urdu [mɑɾnə]

- - 1. Basic Concept: "knee"

**Cognate Set "2000":** Italian [roˈtɛlla], Spanish [roˈðiʎa]

**Cognate Set "1997":** Bulgarian [koljano], Czech [kolɛno], Latvian [celis], Lithuanian_ST [‘kælɪs], Polish [kɔˈlanɔ], Russian [kolʲeˈno]

**Cognate Set "1775":** Albanian_Standard [gju], Armenian_Mod [ʦunk], Catalan [ʒəˈnoʎ], Danish [knε], Dutch_List [kni], English [ni:], French [ʒənu], German [kni:], Italian [dʒiˈnɔkkjo], Provencal [(d)ʒeˈnul], Rumanian_List [dʒeˈnunkʲ], Swedish [knɛ:], Welsh_N [glin]

- - 1. Basic Concept: "know"

**Cognate Set "880":** Rumanian_List [a_ʃti], Sardinian_C [SIRI]

**Cognate Set "2949":** Catalan [səˈße], French [savwaʀ], Italian [saˈpere], Portuguese_ST [sɐˈbeɾ], Provencal [SABE], Spanish [saˈβeɾ]

**Cognate Set "1800":** English [nəʊ], Digor_Ossetic [zon-], Hindi [dʒan:a], Kurdish [zanîn], Persian [DANESTAN], Urdu [ʤɑnnə], Bulgarian [ˈznajɐ], Czech [zna:t], Latvian [zina], Lithuanian_ST [ʒʲɪˈno:tʲɪ], Polish [znaʨ], Russian [znatʲ], Serbocroatian [ZNATI], Slovak [ZNAT], Slovenian [ZNAS], Ukrainian [ZNATY]

**Cognate Set "1062":** Armenian_Mod [gitɛˈnɑl], Breton_List [GOUZOUT], Byelorussian [VEDACˈ], Czech [vjɛɟɛt], Danish [ˈvi:ðə], Dutch_List [ˈwetə(n)], Faroese [VITA], Frisian [ˈvɪtə], German [ˈvɪsn̩], Icelandic_ST [ˈvɪːta], Irish_A [TA_A_FHIOS_AIGE], Norwegian [VITE], Polish [ˈvʲɛʥɛʨ], Slovak [VEDIET], Swedish [ˈve:ta], Welsh_N [GWYBOD]

- - 1. Basic Concept: "lake"

**Cognate Set "370":** Faroese [vatn], Icelandic_ST [vaʰtn], Norwegian [VANN]

**Cognate Set "320":** German [ze:], Danish [so], Swedish [ɧøː]

**Cognate Set "1987":** Pashto [DARJACA], Persian [DARYACHE]

**Cognate Set "147":** Bulgarian [ˈɛzɛrɔ], Byelorussian [VOZERA], Czech [jɛzɛro], Latvian [ęzęrs], Lithuanian_ST [‘æ:ʒʲɛɾɐs], Polish [jɛˈʑɔrɔ], Russian [ˈozʲero], Serbocroatian [JEZERO], Slovak [JAZERO], Slovenian [JEZERO], Ukrainian [OZERO]

**Cognate Set "1008":** Lithuanian_ST [ʊpʲe:], Persian [âb]

***Cognate Set "2950":** English [leɪk], Breton_List [LENN], Irish_A [LOCH], Welsh_N [LLYN], Catalan [ʎak], French [lak], Italian [ˈlago], Portuguese_ST [ˈlagu], Provencal [LAU], Rumanian_List [lac], Sardinian_C [LAGU], Spanish [ˈlaɣo]

***Cognate Set "1985":** Pashto [DZIHIL], Hindi [JHIL], Urdu [ʤʰil]

- - 1. Basic Concept: "laugh"

**Cognate Set "2952":** Catalan [ˈriuwɾə], French [ʀiʀ], Italian [ˈridere], Portuguese_ST [ɾiɾ], Provencal [RIRE], Rumanian_List [a_râde], Sardinian_C [ARRIRI], Spanish [reˈiɾ]

**Cognate Set "2852":** Hindi [HESNA], Urdu [hə̃snə]

**Cognate Set "2772":** Pashto [XANDEL], Persian [KHANDIDAN]

**Cognate Set "2767":** Armenian_Mod [ʦiʦɑˈʁɛl], Greek_Mod [ʝe̞ˈlo̞]

**Cognate Set "23":** Breton_List [CˈHOARZIN], Welsh_N [CHWERTHIN]

**Cognate Set "1809":** Danish [le], Dutch_List [ˈlɑχə(n)], English [lɑ:f], Faroese [LAEA], Frisian [ˈlaitsjə], German [ˈlaxn̩], Icelandic_ST [ˈl̥aiːja], Norwegian [LE]

**Cognate Set "148":** Bulgarian [ˈsmɛjɐ_sɛ], Byelorussian [CˈMJAJACCA], Czech [smaːt_sɛ], Latvian [smejas], Polish [ɕmʲaʨ_ɕɛ̃], Russian [smʲeˈjatʲsʲa], Serbocroatian [SMIJATI_SE], Slovak [SMIAT_SA], Slovenian [SMEJAT], Ukrainian [SMIJATYCˈ]

- - 1. Basic Concept: "leaf"

**Cognate Set "719":** English [li:f], Latvian [lapa], Lithuanian_ST [LAPAS]

**Cognate Set "2854":** Hindi [pətːiː], Urdu [pətːɑ]

**Cognate Set "24":** Breton_List [DEIL], Irish_A [DUILLEOG], Welsh_N [DEILEN]

**Cognate Set "1812":** Kurdish [pêl], Greek_Mod [ˈfilo̞], Catalan [ˈfuʎa], French [fœj], Italian [ˈfɔʎʎo], Portuguese_ST [ˈfoʎɐ], Provencal [FUEIO], Rumanian_List [FOAIE], Sardinian_C [FOLLA], Spanish [ˈoxa], Danish [blad], Dutch_List [blɑt], Faroese [blɛaː], Frisian [blɛ:t], German [blat], Icelandic_ST [plaːð], Norwegian [blad], Swedish [blɑ:d]

**Cognate Set "150":** Bulgarian [list], Byelorussian [LIST], Czech [lɪst], Polish [ʎiɕʨ], Russian [list], Serbocroatian [LIST], Slovak [LIST], Ukrainian [LYST]

- - 1. Basic Concept: "left"

**Cognate Set "769":** Welsh_N [CHWITH], Lithuanian_ST [KAIRYS]

**Cognate Set "395":** French [goʃ], Provencal [GAUCHE]

**Cognate Set "321":** Danish [ˈvεnsd̥ʰʁʌ], Faroese [VINSTRI], Icelandic_ST [VINSTRI], Norwegian [VENSTRE], Swedish [ˈvɛnstər]

**Cognate Set "2954":** Catalan [siˈnistɾə], Italian [siˈnistro]

**Cognate Set "2855":** Hindi [BAYA], Urdu [bɑjɑ̃]

**Cognate Set "25":** Breton_List [KLEIZ], Irish_A [CLE]

**Cognate Set "1995":** Catalan [əsˈkɛrə], Portuguese_ST [ɨʃˈkeɾdɐ], Spanish [iθˈkjeɾða]

**Cognate Set "1817":** Dutch_List [lɪŋks], Frisian [lɪɪks], German [lɪŋk]

**Cognate Set "151":** Bulgarian [ljaf], Byelorussian [LEVY], Czech [lɛviː], Polish [ˈlɛvɨ], Russian [ˈlʲevɨj], Serbocroatian [LJEVICA], Slovak [L_AVY], Slovenian [LEVIC], Ukrainian [LIVYJ]

- - 1. Basic Concept: "leg"

**Cognate Set "954":** Irish_A [COS], Welsh_N [COES]

**Cognate Set "720":** Latvian [kãja], Lithuanian_ST [KOJA]

**Cognate Set "591":** Hindi [TAG], Urdu [ʈɑŋɡ]

**Cognate Set "2955":** Portuguese_ST [ˈpɛɾnɐ], Spanish [ˈpjeɾna]

**Cognate Set "2799":** Danish [ben], Dutch_List [ben], Faroese [bain], Frisian [ˈboŋkjə], German [ba‿in], Norwegian [BEN], Swedish [beːn]

**Cognate Set "233":** Byelorussian [NAGA], Czech [noɦa], Polish [ˈnɔɡa], Russian [noˈga], Serbocroatian [NOGA], Slovak [NOHA], Slovenian [NOGA], Ukrainian [NOGA]

**Cognate Set "1996":** Rumanian_List [PICIOR], Armenian_Mod [votkʰ], Greek_Mod [ˈpo̞ði], Icelandic_ST [ˈfouːtʏr], Persian [pɒ]

***Cognate Set "353":** Faroese [ˈlɛɡːʊr], English [lɛg]

***Cognate Set "266":** Albanian_Standard [këmbë], Catalan [ˈkamə], French [ʒɑ̃b], Italian [ˈgamba], Provencal [GARRO], Provencal [ˈkambo], Sardinian_C [KAMBA]

- - 1. Basic Concept: "lie"

**Cognate Set "838":** Kurdish [xwe_dirêj_kirin], Persian [KHABIDAN_(DERAZ_KASHIDAN)]

**Cognate Set "721":** Latvian [gul_̜], Lithuanian_ST [GULETI]

**Cognate Set "592":** Hindi [leʈna], Urdu [leʈnə]

**Cognate Set "2956":** Catalan [ˈʒɛuɾə], Italian [dʒaˈtʃere], Provencal [JAIRE], Spanish [YACER]

**Cognate Set "27":** Breton_List [SOUCHA], Welsh_N [GORWEDD]

**Cognate Set "1827":** Irish_A [LUIGHE], Bulgarian [lɛˈʒɐ], Czech [lɛʒɛt], Polish [ˈlɛʒɛʨ], Russian [ˈlʲeʒatʲ], Serbocroatian [LEZATI], Slovak [LEZAT], Slovenian [LEZI], Ukrainian [LEZATY], Danish [ˈleg̥ə], Dutch_List [ˈlɪɣə(n)], English [laɪ], Faroese [LIGGJA], German [ˈli:gn̩], Icelandic_ST [ˈlɪcːa], Norwegian [LIGGE], Swedish [ˈliga]

**Cognate Set "1063":** Rumanian_List [a_se_culca], French [sə_kuʃe], Provencal [COUCHA]

- - 1. Basic Concept: "live"

**Cognate Set "2804":** Breton_List [BEVA], Bulgarian [ʒiˈvɛjɐ], Byelorussian [ZYCˈ], Catalan [ˈbiwɾə], Czech [ʒiːt], French [vivʀ], Greek_Mod [zo̞], Hindi [JINA_(BE_ALIVE)], Italian [ˈvivere], Latvian [dzîvõ], Lithuanian_ST [ɡi’ʋæntɪ], Pashto [ZVAND_KAVEL], Persian [ZENDEGI_KARDAN], Polish [ʒɨʨ], Portuguese_ST [viˈveɾ], Provencal [VIEURE], Russian [ʒɨtʲ], Sardinian_C [BIVI], Serbocroatian [ZIVITI], Slovak [ZIT], Slovenian [ZIVI], Spanish [biˈβiɾ], Ukrainian [ZYTY], Urdu [ʤinə], Welsh_N [BYW]

**Cognate Set "1835":** Danish [ˈle:və], Dutch_List [levə(n)], English [lɪv], Faroese [LIVA], Frisian [LEVE], German [ˈle:bn̩], Icelandic_ST [LIFA], Norwegian [LEVE], Swedish [ˈle:va]

- - 1. Basic Concept: "liver"

**Cognate Set "2957":** Catalan [ˈfedʒə], French [fwa], Italian [ˈfegato], Portuguese_ST [ˈfiɣɐðu], Provencal [FEGE], Rumanian_List [ficat], Sardinian_C [FIGAU], Spanish [ˈiɣaðo]

**Cognate Set "28":** Breton_List [AVU], Irish_A [AE], Welsh_N [IAU]

**Cognate Set "2797":** Latvian [aknas], Digor_Ossetic [igɐr], Kurdish [ceger], Persian [JEGAR], Armenian_Mod [lɪ̯ɑrtʰ]

**Cognate Set "255":** Slovak [PECEN], Russian [ˈpʲetʃenʲ], Ukrainian [PECINKA]

**Cognate Set "234":** Byelorussian [VANTROBA], Czech [jaːtra], Polish [vɔ̃ˈtrɔba], Serbocroatian [JETRA], Slovenian [JTRA]

**Cognate Set "1845":** Danish [ˈlewʌ], Dutch_List [ˈlevər], English [ˈlɪvə], Faroese [ˈliːvʊr], Frisian [ˈle:vər], German [ˈle:bɐ], Icelandic_ST [ˈlɪːvʏr], Norwegian [lever], Swedish [ˈle:vər]

- - 1. Basic Concept: "long"

**Cognate Set "29":** Breton_List [HIR], Welsh_N [HIR]

**Cognate Set "2856":** Hindi [LEMBA], Urdu [ˈləm.bə]

**Cognate Set "267":** Catalan [ʎaɾk], Spanish [ˈlaɾɣo]

**Cognate Set "2410":** Kurdish [bilind], Persian [bolænd]

**Cognate Set "1851":** Albanian_Standard [i_gjatë], Bulgarian [ˈdɤ̞lɤk], Byelorussian [DAWHI], Czech [dloʊ̯ɦiː], Danish [lɑŋ], Digor_Ossetic [darʁ], Dutch_List [lɑŋ], English [lɒŋ], Faroese [LANGUR], French [lɔ̃], Frisian [laŋ], German [laŋ], Icelandic_ST [ˈlaŋkʏr], Italian [ˈlungo], Lithuanian_ST [ILGAS], Norwegian [LANG], Persian [DERAZ], Polish [ˈdwuɡi], Portuguese_ST [ˈlõɣu], Provencal [LONG], Rumanian_List [lung], Russian [ˈdlinnɨj], Sardinian_C [LONGU], Serbocroatian [DUG], Slovak [DLHY], Slovenian [DOVGU], Swedish [lɔŋ], Ukrainian [DOVGYJ]

- - 1. Basic Concept: "louse"

**Cognate Set "722":** Latvian [uts], Lithuanian_ST [UTELE]

**Cognate Set "581":** Hindi [dʒũː], Urdu [ʤũ]

**Cognate Set "2958":** Catalan [poʎ], French [pu], Italian [piˈdɔkkjo], Portuguese_ST [piˈoʎu], Provencal [PESOU], Rumanian_List [păduche], Sardinian_C [PRIOGU], Spanish [ˈpjoxo]

**Cognate Set "1859":** Breton_List [LAOU], Welsh_N [LLO], Bulgarian [ˈvɤ̞ʃkɐ], Byelorussian [VOS], Czech [vɛʃ], Polish [vɛʃ], Russian [voʃ], Serbocroatian [US], Slovak [VOS], Slovenian [USI], Ukrainian [VOSA], Danish [lus], Dutch_List [vlo], English [laʊs], Faroese [lʉus], Frisian [lu:s], German [la‿us], Icelandic_ST [luːs], Norwegian [LUS], Swedish [lʉ:s]

****Cognate Set "2828":** Kurdish [sipî], Pashto [SPEZA], Persian [ʃeˈpeʃ], Digor_Ossetic [šistɐ]

- - 1. Basic Concept: "man"

**Cognate Set "87":** Irish_A [FEAR], Welsh_N [gu:r], Latvian [vĩrs], Lithuanian_ST [‘ʋʲi:rɐs]

**Cognate Set "2959":** Breton_List [DEN], Welsh_N [DYN], Catalan [ˈɔmə], French [ɔm], Italian [ˈwɔmo], Portuguese_ST [ˈɔmɐ̃ĩ], Provencal [ˈɔme], Sardinian_C [OMINI], Spanish [ˈombɾe]

**Cognate Set "2835":** Pashto [SARAJ], Bulgarian [ˈmɤ̞ʃ], Byelorussian [MUZCYNA], Czech [mʊʃ], Polish [mɛ̃ˈʃʧɨzna], Russian [muʒˈtʃɨna], Slovak [MUZ], Slovenian [MOZ], Danish [man], Dutch_List [mɑn], English [mæn], Faroese [KALLMADUR], Faroese [ˈmɛavʊr], Frisian [mɔn], German [man], Icelandic_ST [ˈkʰartlˌmaːðʏr], Icelandic_ST [ˈmaːðʏr], Norwegian [mann], Swedish [man]

**Cognate Set "1075":** Hindi [ADMI], Persian [ADAM], Urdu [ʔɑd̪mi]

**Cognate Set "1074":** Armenian_Mod [təʁɑˈmɑɾtʰ], Kurdish [mêr], Persian [mæɾd], Urdu [məɾd̪]

**Cognate Set "1070":** Faroese [KALLMADUR], Icelandic_ST [KARL], Icelandic_ST [ˈkʰartlˌmaːðʏr], Swedish [kɑ:r]

**Cognate Set "1069":** Serbocroatian [COVEK], Byelorussian [CALAVEK]

- - 1. Basic Concept: "many"

**Cognate Set "723":** Latvian [daũdzi], Lithuanian_ST [dɐuɡ], Polish [ˈduʒɔ], Slovenian [DOSTI]

**Cognate Set "2960":** Catalan [moɫt], Italian [ˈmolti], Portuguese_ST [ˈmũĩtu], Provencal [MANT], Rumanian_List [mulʦʲ], Spanish [ˈmuʧos]

**Cognate Set "2798":** Hindi [bəˈɦʊt], Urdu [bəˈhʊt̪]

**Cognate Set "1867":** Greek_Mod [po̞liˈariθˌmo̞s], Breton_List [KALZ], Dutch_List [vel], German [fi:l]

**Cognate Set "154":** English [ˈmɛnɪ], Danish [ˈmɑŋən], Faroese [MANGUR], Icelandic_ST [MARGIR], Norwegian [mange], Swedish [ˈmɔŋa], Bulgarian [ˈmnɔɡo], Czech [mnoɦo], Russian [ˈmnogo], Serbocroatian [MNOGI], Slovak [MNOHY], Slovak [NEJEDEN]

**Cognate Set "1081":** Irish_A [GO_LEOR], Welsh_N [LLAWER]

**Cognate Set "1080":** Polish [ˈvʲɛlɛ], Slovak [VEL_A]

- - 1. Basic Concept: "meat"

**Cognate Set "983":** French [vjɑ̃d], Provencal [VIANDO]

**Cognate Set "371":** Faroese [hold], Icelandic_ST [HOLD]

**Cognate Set "322":** Danish [kø̞ð], Faroese [tʃøːt], Icelandic_ST [kʰjœːt], Norwegian [kjøtt], Swedish [ɕœtː]

**Cognate Set "30":** Breton_List [KIG], Welsh_N [CIG]

**Cognate Set "2961":** Catalan [kaɾn], Italian [ˈkarne], Portuguese_ST [ˈkaɾnɨ], Provencal [CAR], Rumanian_List [ˈkarne], Spanish [ˈkaɾne]

**Cognate Set "2853":** Kurdish [goşt], Pashto [GVASA], Persian [ɡuʃt], Urdu [ɡoʃt]

**Cognate Set "2571":** Albanian_Standard [mish], Armenian_Mod [mis], Bulgarian [mɛˈsɔ], Byelorussian [MJASA], Czech [maso], Hindi [MAS], Lithuanian_ST [MESA], Polish [ˈmʲɛ̃sɔ], Russian [ˈmʲaso], Serbocroatian [MESO], Slovak [MASO], Slovenian [MESO], Swedish [mɑ:t], Ukrainian [MˈJASO]

**Cognate Set "1877":** Dutch_List [vles], Frisian [flais], German [fla‿iʃ]

- - 1. Basic Concept: "moon"

**Cognate Set "1084":** Albanian_Standard [hëna], Urdu [ʧɑnd̪]

**Cognate Set "1083":** Welsh_N [ˈɬəɨad], Russian [luˈna], French [lyn], Provencal [ˈlyno], Rumanian_List [ˈlunə], Spanish [ˈluna], Armenian_Mod [luˈsin]

**Cognate Set "1007":** Armenian_Mod [amis], Kurdish [meh], Czech [mɲɛsiːts], Dutch_List [maan], English [moon], Faroese [ˈmɔanɪ], German [Mond], Swedish [ˈmoːˌnɛ], Latvian [mẽness], Lithuanian_ST [mʲe:’nʊlʲɪs]

- - 1. Basic Concept: "mother"

**Cognate Set "414":** Frisian [mɛm], Breton_List [MAMM], Welsh_N [mam], Rumanian_List [mamă], Sardinian_C [MAMMA]

**Cognate Set "2860":** Armenian_Mod [maɪ̯ɾ], Byelorussian [MACI], Catalan [ˈmaɾə], Czech [matka], Danish [moɐ̯], Digor_Ossetic [madɐ], Dutch_List [ˈmudər], English [ˈmʌðə], Faroese [ˈmɔuwɪr], French [mɛʀ], German [ˈmʊtɐ], Greek_Mod [miˈte̞ra], Hindi [MA], Icelandic_ST [ˈmouːðɪr], Irish_A [MATHAIR], Italian [ˈmadre], Latvian [mãte], Lithuanian_ST [MOTINA], Norwegian [MOR], Pashto [MOR], Persian [mɒːdær], Polish [ˈmatka], Portuguese_ST [mɐ̃i], Provencal [ˈmajɾe], Russian [matʲ], Slovak [MATKA], Slovenian [MATI], Spanish [ˈmaðɾe], Swedish [muːr], Swedish [ˈmu:dər], Ukrainian [ˈmatɪ], Urdu [mɑ̃]

**Cognate Set "155":** Bulgarian [ˈmajkɐ], Serbocroatian [MAJKA]

- - 1. Basic Concept: "mountain"

**Cognate Set "809":** Kurdish [sax], Persian [KUH]

**Cognate Set "724":** Latvian [kal_̂-ns], Lithuanian_ST [‘ka:lnɐs]

**Cognate Set "2870":** Byelorussian [HARA], Czech [ɦora], Polish [ˈɡura], Russian [goˈra], Serbocroatian [GORA], Slovak [HORA], Slovenian [GORE], Ukrainian [HORA], Pashto [GAR]

**Cognate Set "2009":** Faroese [fjadl], Icelandic_ST [fjatl], Norwegian [FJELL]

**Cognate Set "2007":** Hindi [pəhɑːɽ], Urdu [pəhɑɽ]

**Cognate Set "1884":** Danish [ˈb̥jɛɐ̯w], Dutch_List [bɛrχ], Frisian [bɛrx], German [bɛrk], Swedish [ˈbærj]

***Cognate Set "2962":** English [ˈmaʊntɪn], Breton_List [MENEZ], Welsh_N [MYNYDD], Catalan [mon], French [mɔ̃taɲ], Italian [monˈtaɲɲa], Portuguese_ST [mõˈtɐɲɐ], Provencal [MOUNTAGNO], Rumanian_List [munte], Sardinian_C [MONTI], Spanish [monˈtaɲa]

- - 1. Basic Concept: "mouth"

**Cognate Set "31":** Breton_List [GENOU], Welsh_N [genou]

**Cognate Set "2963":** Catalan [ˈbokə], French [buʃ], Italian [ˈbokka], Portuguese_ST [ˈbokɐ], Provencal [BOUCO], Sardinian_C [BUKKA], Spanish [ˈboka]

**Cognate Set "2857":** Bulgarian [oˈsta], Czech [uːsta], Polish [ˈusta], Serbocroatian [USTA], Slovak [USTA], Slovenian [VUJSTA]

**Cognate Set "2800":** Digor_Ossetic [kom], Kurdish [dev]

**Cognate Set "2706":** Armenian_Mod [bɛˈɾɑn], Lithuanian_ST [bʊr’nɐ]

**Cognate Set "235":** Byelorussian [ROT], Russian [rot], Ukrainian [ROT]

**Cognate Set "1888":** Danish [mɔn], Dutch_List [mɔnt], English [maʊθ], Faroese [MUNNUR], German [mʊnt], Icelandic_ST [ˈmʏnːʏr], Norwegian [MUNN], Swedish [mɵn]

**Cognate Set "1086":** Hindi [mʊkʰ], Urdu [mʊ̃ʰ], Frisian [mulə], Latvian [mute]

***Cognate Set "2575":** Rumanian_List [gură], Albanian_Standard [gojë]

- - 1. Basic Concept: "name"

**Cognate Set "725":** Latvian [vā̀rds], Lithuanian_ST [ˈvardas]

**Cognate Set "2882":** Albanian_Standard [emër], Armenian_Mod [ɑˈnun], Breton_List [HANO], Bulgarian [ˈimɛ], Byelorussian [IMJA], Catalan [nɔm], Czech [jmɛːno], Danish [nɑwn], Digor_Ossetic [nom], Dutch_List [nam], English [neɪm], Faroese [naun], French [nɔ̃], Frisian [ˈnamə], German [ˈna:mə], Greek_Mod [ˈo̞no̞ˌma], Hindi [na:m], Icelandic_ST [napn], Irish_A [AINM], Italian [ˈnome], Kurdish [nav], Norwegian [navn], Pashto [NUM], Polish [ˈimʲɛ̃], Portuguese_ST [ˈnɔmɨ], Provencal [NOUM], Rumanian_List [ˈnume], Russian [ˈimʲa], Sardinian_C [NOMINI], Serbocroatian [IME], Slovak [MENO], Slovenian [IMJ], Spanish [ˈnombɾe], Swedish [namn], Ukrainian [IMˈJA], Urdu [nɑm], Welsh_N [ENW]

- - 1. Basic Concept: "narrow"

**Cognate Set "644":** Irish_A [CAOL], Welsh_N [CUL]

**Cognate Set "324":** Danish [smal], Norwegian [SMAL], Swedish [smɑ:l]

**Cognate Set "2018":** Faroese [TRONGUR], Icelandic_ST [ˈθrøyŋkʏr], Swedish [trɔŋ]

**Cognate Set "2017":** Icelandic_ST [MJOR], Breton_List [MOAN]

**Cognate Set "2015":** Armenian_Mod [nɛʁ], English [ˈnærəʊ]

**Cognate Set "2013":** Dutch_List [nɑu], Frisian [nɔ.u]

**Cognate Set "2012":** Latvian [šàurs], Lithuanian_ST [SIAURAS]

**Cognate Set "2011":** Catalan [əˈstɾɛt], French [etʀwa], Italian [ˈstretto], Portuguese_ST [ɨʃˈtɾɐitu], Provencal [ESTRE], Rumanian_List [STRIMT], Sardinian_C [STRINTU], Spanish [esˈtɾeʧo], Breton_List [STRIZ]

**Cognate Set "2010":** Bulgarian [ˈtɛsɛn], Slovenian [TESEN]

***Cognate Set "2890":** Pashto [TANG], Hindi [TENG], Urdu [t̪əŋ]

****Cognate Set "1894":** Breton_List [ENK], Byelorussian [VUZKI], Czech [uːskiː], Dutch_List [ɛŋ], German [ɛŋ], Lithuanian_ST [ANKSTAS], Polish [ˈvɔ̃ski], Portuguese_ST [APERTADO], Provencal [ECHO], Rumanian_List [INGUST], Russian [ˈuzkɨj], Serbocroatian [UZAK], Slovak [UZKY], Slovenian [VASKO], Spanish [ANGOSTO], Ukrainian [VUZˈKYJ], Albanian_Standard [e_ngushtë]

- - 1. Basic Concept: "near"

**Cognate Set "268":** Catalan [əˈpɾɔp], Provencal [PROCHE], Rumanian_List [apropiat]

**Cognate Set "1901":** Danish [nεɐ̯], Dutch_List [naˈbɛi], English [nɪə], Faroese [NAR], German [ˈna:ə], Icelandic_ST [ˈnauːlaiːɣʏr], Norwegian [NAER], Swedish [ˈnæ:ra]

**Cognate Set "156":** Bulgarian [ˈblizɤk], Byelorussian [BLIZKI], Czech [bliːskiː], Polish [ˈbʎiskɔ], Russian [ˈblizko], Serbocroatian [BLIZU], Slovak [BLIZKY], Slovenian [BLIZII], Ukrainian [BILJA]

****Cognate Set "2901":** Pashto [NEZDE], Persian [NAZDIK], Urdu [nəzd̪ik]

- - 1. Basic Concept: "neck"

**Cognate Set "726":** Latvian [kakls], Lithuanian_ST [KAKLAS]

**Cognate Set "33":** Breton_List [GOUZOUG], Welsh_N [GWDDF]

**Cognate Set "2911":** Bulgarian [vrat], Serbocroatian [VRAT], Slovenian [VRAT], Hindi [GERDEN], Kurdish [gerden], Pashto [GARA], Persian [GARDAN], Urdu [ɡəɾd̪ən]

**Cognate Set "208":** Czech [SIJE], Polish [ˈʃɨja], Russian [ˈʃeja], Ukrainian [SYJA]

**Cognate Set "1906":** Catalan [kɔʎ], French [ku], Italian [ˈkollo], Provencal [COU], Spanish [ˈkweʎo], Danish [hals], Dutch_List [hɑls], Faroese [HALSUR], Frisian [hɔls], German [hals], Icelandic_ST [hauls], Norwegian [HALS], Swedish [hals]

**Cognate Set "1091":** Byelorussian [KARAK], Czech [kr̩k], Slovak [KRK]

**Cognate Set "1089":** Dutch_List [nɛk], English [nɛk], Frisian [ˈnɛkə], German [ˈnakn̩]

- - 1. Basic Concept: "new"

**Cognate Set "2922":** Armenian_Mod [noɾ], Breton_List [NEVEZ], Bulgarian [nɔf], Byelorussian [NOVY], Catalan [nɔuw], Czech [noviː], Danish [ny], Digor_Ossetic [nɐwɐg], Dutch_List [niw], English [nju:], Faroese [NYGGJUR], French [nuvo], Frisian [nɛi], German [nɔ‿y], Hindi [n̪əja:], Icelandic_ST [niːr], Irish_A [NUA], Italian [ˈnwɔvo], Kurdish [nû], Lithuanian_ST [NAUJAS], Norwegian [NY], Pashto [NEVAJ], Polish [ˈnɔvɨ], Portuguese_ST [ˈnovu], Provencal [NOUVEU], Rumanian_List [NOU], Russian [ˈnovɨj], Sardinian_C [NOU], Serbocroatian [NOV], Slovak [NOVY], Slovenian [NOVA], Spanish [ˈnweβo], Swedish [ny], Ukrainian [NOVYJ_-_A_-_E], Urdu [nəjɑ], Welsh_N [NEWYDD]

- - 1. Basic Concept: "night"

**Cognate Set "2932":** Digor_Ossetic [ɐχsɐvɐ], Kurdish [şev], Pashto [SPA], Persian [SHAB]

**Cognate Set "2859":** Hindi [ra:t̪], Urdu [ɾɑt̪]

**Cognate Set "1914":** Albanian_Standard [natë], Breton_List [NOZ], Bulgarian [nɔʃt], Byelorussian [NOC], Catalan [nit], Czech [nots], Danish [nad̥ʰ], Dutch_List [nɑχt], English [naɪt], Faroese [nɔtː], French [nɥi], Frisian [JOUN], Frisian [naxt], German [naxt], Greek_Mod [nýchta], Icelandic_ST [nouht], Italian [ˈnɔtte], Latvian [nakts], Lithuanian_ST [nɐk’tɪs], Norwegian [NATT], Polish [nɔʦ], Portuguese_ST [ˈnɔjtɨ], Provencal [NUE], Provencal [nɥɛʧ], Rumanian_List [ˈno̯ap.te], Russian [notʃ], Sardinian_C [NOTTI], Serbocroatian [NOC], Slovak [NOC], Slovenian [NOC], Spanish [ˈnoʧe], Swedish [nat], Ukrainian [NIC], Welsh_N [noːs]

- - 1. Basic Concept: "nose"

**Cognate Set "34":** Breton_List [FRI], Irish_A [SRON]

**Cognate Set "2941":** Kurdish [poz], Pashto [PAZA]

**Cognate Set "1922":** Bulgarian [nɔs], Byelorussian [NOS], Catalan [nas], Czech [nos], Danish [ˈnε:sə], Dutch_List [nøs], English [nəʊz], Faroese [nøːs], French [nɛ], Frisian [no.əs], German [ˈna:zə], Hindi [NAK], Italian [ˈnaso], Lithuanian_ST [NOSIS], Norwegian [nese_(nase)], Polish [nɔs], Portuguese_ST [nɐˈɾiʃ], Provencal [NAS], Rumanian_List [nas], Russian [nos], Sardinian_C [NASU], Serbocroatian [NOS], Slovak [NOS], Slovenian [NUS], Spanish [naˈɾiθ], Swedish [ˈnɛːsa], Ukrainian [NIS], Urdu [nɑk]

- - 1. Basic Concept: "not"

**Cognate Set "325":** Danish [eg̥ʰə], Faroese [ˈɪtʃɪ], Icelandic_ST [ˈɛhcɪ], Norwegian [ikke], Swedish [ˈɪntɛ]

**Cognate Set "2951":** Albanian_Standard [nuk], Breton_List [NAMM], Bulgarian [nɛ], Byelorussian [NE], Catalan [no], Czech [nɛ], Digor_Ossetic [nɐ/ma], Dutch_List [nit], English [nɒt], French [nɔ̃], Frisian [ne:], German [nɪçt], Hindi [nəɦĩː], Irish_A [NI], Italian [nɔ], Kurdish [na], Kurdish [ne], Latvian [ne], Lithuanian_ST [NE-_(PREFIX)], Pashto [NA], Persian [NA], Polish [ɲɛ], Portuguese_ST [nɐ̃ũ], Provencal [NOUN], Rumanian_List [nu], Russian [nʲe], Sardinian_C [NO], Serbocroatian [NE], Slovak [NIE], Slovenian [NJE], Spanish [no], Ukrainian [NE], Urdu [nəhĩ]

**Cognate Set "2711":** Greek_Mod [OCHI], Armenian_Mod [voʧʰ]

- - 1. Basic Concept: "old"

**Cognate Set "326":** Danish [ˈg̥ɑməl], Faroese [GAMAL], Icelandic_ST [ˈkaːmatl], Norwegian [GAMMEL], Swedish [ˈgamal]

**Cognate Set "2862":** Hindi [BURHA_(ANIMATE)], Persian [PIR], Urdu [buɽʰɑ]

**Cognate Set "2712":** Armenian_Mod [hin], Lithuanian_ST [‘sʲæ:nɐs], Irish_A [SEAN], Welsh_N [HEN]

**Cognate Set "2025":** Hindi [pʊrana], Urdu [pʊɾɑnɑ]

**Cognate Set "2024":** Greek_Mod [YEROS], Digor_Ossetic [zɐrond], Pashto [ZOR]

**Cognate Set "1931":** Dutch_List [ɑut], English [əʊld], Frisian [ɔːᵘt], German [alt]

**Cognate Set "157":** Bulgarian [star], Byelorussian [STARY], Czech [stariː], Polish [ˈstarɨ], Russian [ˈstarɨj], Serbocroatian [STAR], Slovak [STARY], Slovenian [STAR], Ukrainian [STARYJ]

***Cognate Set "2965":** Latvian [vęcs], Catalan [bɛʎ], French [vjø], Italian [ˈvekkjo], Portuguese_ST [ˈvɛʎu], Provencal [VIEI], Rumanian_List [BATRIN], Rumanian_List [vechi], Sardinian_C [BECCU], Spanish [ˈbjexo], Albanian_Standard [i_ftohtë]

- - 1. Basic Concept: "one"

**Cognate Set "2971":** Armenian_Mod [mɛk], Breton_List [UNAN], Bulgarian [ɛˈdin], Byelorussian [ADZIN], Catalan [un], Czech [jɛdɛn], Danish [en], Digor_Ossetic [jew], Dutch_List [en], English [wʌn], Faroese [EIN], French [œ̃], German [a‿in], Hindi [EK], Icelandic_ST [EINN], Irish_A [AON], Italian [ˈuno], Kurdish [yek], Kurdish [êk], Latvian [viêns], Lithuanian_ST [NIENAS], Norwegian [en], Pashto [JAV], Persian [jek], Polish [ˈjɛdɛn], Portuguese_ST [ũ], Provencal [UN], Rumanian_List [uˈnu], Russian [oˈdin], Sardinian_C [UNU], Serbocroatian [JEDAN], Slovak [JEDEN], Slovenian [ADEN], Spanish [ˈuno], Swedish [ɛn], Ukrainian [ODYN], Urdu [ʔek], Welsh_N [UN]

**Cognate Set "2580":** Armenian_Mod [MI], Albanian_Standard [një]

- - 1. Basic Concept: "other"

**Cognate Set "727":** Latvian [cits], Lithuanian_ST [KITAS]

**Cognate Set "593":** Hindi [DUSRA], Urdu [ˈd̪us.ɾɑ]

**Cognate Set "2714":** Armenian_Mod [ɑɪ̯l], Breton_List [ALL], French [otʀ], Greek_Mod [ˈalo̞s], Irish_A [EILE], Italian [ˈaltro], Portuguese_ST [ˈo(ou)tɾu], Provencal [AUTRE], Rumanian_List [alt], Sardinian_C [ATRU], Spanish [ˈotɾo], Welsh_N [LLALL]

**Cognate Set "2029":** Dutch_List [nɔχ_en], Byelorussian [INSY], Czech [jɪniː], Polish [ˈinnɨ], Slovak [INY], Ukrainian [INSYJ]

**Cognate Set "1937":** Digor_Ossetic [ɐndɐr/innɐ], Danish [ˈanən], Dutch_List [ˈɑndər], English [ˈʌðə], Faroese [ANNAR], Frisian [o.ər], German [ˈandɐ], Icelandic_ST [ˈanːar], Norwegian [ANNEN], Swedish [ˈanan]

**Cognate Set "158":** Bulgarian [druk], Czech [DRUHY], Polish [ˈdruɡi], Russian [druˈgoj], Serbocroatian [DRUGI], Slovenian [DRUGI]

- - 1. Basic Concept: "person"

**Cognate Set "36":** Spanish [ˈombɾe], Breton_List [DEN], Irish_A [DUINE], Rumanian_List [om], Provencal [ˈɔme]

**Cognate Set "2992":** Dutch_List [mɛns], Faroese [MADUR], Frisian [mẽːskə], German [mɛnʃ], Icelandic_ST [ˈmaːðʏr], Norwegian [mennske]

**Cognate Set "236":** Byelorussian [ASOBA], Czech [OSOBA], Serbocroatian [OSOBA], Slovak [OSOBA], Slovenian [OSJBA], Ukrainian [OSOBA]

**Cognate Set "1108":** Hindi [INSAN], Urdu [ʔɪnsɑn]

***Cognate Set "2966":** Welsh_N [PERSON], English [ˈpɜ:sən], Danish [b̥ʰæɐ̯ˈson], Swedish [pæˈʂu:n], Catalan [pəɾˈsonə], French [pɛʀsɔn], Italian [perˈsona], Portuguese_ST [PESSOA], Provencal [PERSOUNO], Rumanian_List [PERSOANA], Sardinian_C [PERSONA], Spanish [PERSONA]

****Cognate Set "1103":** Bulgarian [ʧovɛk], Polish [ˈʧwɔvʲjɛk], Russian [tʃeloˈvʲek], Latvian [cìlvę̄ks]

- - 1. Basic Concept: "play"

**Cognate Set "38":** Breton_List [CˈHOARI], Welsh_N [CHWARAE]

**Cognate Set "3002":** Pashto [BAZI_KAVEL], Persian [BAZI_KARDAN]

**Cognate Set "2967":** Catalan [ʒuˈɣa], French [ʒwe], Italian [dʒoˈkare], Portuguese_ST [ʒuˈɡaɾ], Provencal [JOUGA], Rumanian_List [a_juca], Sardinian_C [GOGAI], Spanish [xuˈɣaɾ]

**Cognate Set "2863":** Hindi [KHELNA], Urdu [bəʤɑnə]

**Cognate Set "2031":** Danish [ˈlɑjə], Faroese [LEIKA], Icelandic_ST [LEIKA_SER], Norwegian [LEKE], Swedish [ˈle:ka]

**Cognate Set "2030":** Byelorussian [ZABAWLJACCA], Polish [ˈbavʲiʨ_ɕɛ], Slovak [BAVIT__SA]

**Cognate Set "161":** Bulgarian [iˈɡrajɐ], Czech [ɦraːt], Polish [graʨ], Russian [iˈgratʲ], Serbocroatian [IGRATI_SE], Slovak [HRAT], Slovenian [SE_JGRAT], Ukrainian [HRATY]

***Cognate Set "1945":** Dutch_List [ˈspelə(n)], Frisian [ˈspiljə], German [ˈʃpi:lən], Swedish [ˈspe:la], Latvian [spẽlẽjas]

- - 1. Basic Concept: "pull"

**Cognate Set "39":** Breton_List [TENNA], Welsh_N [TYNNU]

**Cognate Set "372":** German [ˈt͜si:ən], Faroese [TOGA], Icelandic_ST [ˈtʰɔːɣa]

**Cognate Set "3013":** Pashto [KSEL], Persian [KASHIDAN]

**Cognate Set "2864":** Hindi [KHICNA], Urdu [kʰenʧnə]

**Cognate Set "269":** Catalan [əstiˈɾa], Catalan [ˈtɾɛuwɾə], French [tiʀe], Italian [tiˈrare], Provencal [TIRA], Sardinian_C [TIRAI], Spanish [tiˈɾaɾ]

**Cognate Set "2584":** Serbocroatian [VUCI], Albanian_Standard [tërheq], Latvian [vę̀lk]

**Cognate Set "2034":** Rumanian_List [a_trage], Faroese [DRAGA], Icelandic_ST [ˈtraːɣa], Swedish [drɑ:]

**Cognate Set "2033":** Byelorussian [CJAHNUCˈ], Czech [taːɦnoʊ̯t], Polish [ˈʨɔ̃ɡnɔ̃ʨ], Russian [ˈtʲanutʲ], Slovak [T_AHAT], Ukrainian [TJAHNUTY]

**Cognate Set "1951":** Danish [d̥ʰʁag̥ʰə], Dutch_List [ˈtrɛkə(n)], Frisian [ˈlukə], Frisian [ˈtrɛkə], Norwegian [TREKKE]

- - 1. Basic Concept: "push"

**Cognate Set "687":** Italian [ˈspindʒere], Rumanian_List [a_împinge], Sardinian_C [SPINGI]

**Cognate Set "40":** Provencal [BUTA], Breton_List [LUSKA]

**Cognate Set "343":** Norwegian [SKYVE], Dutch_List [ˈsχœyvən]

**Cognate Set "288":** Czech [STRKATI], Slovak [STRKAT]

**Cognate Set "270":** Catalan [əmˈpɛɲə], Provencal [EMPEGNE]

**Cognate Set "238":** Byelorussian [PXACˈ], Polish [pxaʨ], Ukrainian [PXATY]

**Cognate Set "209":** Czech [tlatʃɪt], Russian [tolˈkatʲ]

**Cognate Set "2037":** Albanian_Standard [shtyj], Latvian [stumj], Lithuanian_ST [STUMTI], Danish [ˈsd̥ø:ðə], Dutch_List [ˈdywə(n)], German [ˈʃto:sn̩], Swedish [ˈstø:ta]

***Cognate Set "398":** English [pʊʃ], French [puse], Spanish [empuˈxaɾ]

- - 1. Basic Concept: "rain"

**Cognate Set "729":** Latvian [LIT_(LIETUS)], Latvian [liêtus], Lithuanian_ST [ljɛˈtʊs]

**Cognate Set "41":** Breton_List [GLAOIA], Welsh_N [BWRWGLAW]

**Cognate Set "2970":** Catalan [ˈplɔuwɾə], French [pløvwaʀ], Italian [ˈpjɔvere], Provencal [PLOURE], Rumanian_List [A_PLOUA], Rumanian_List [ˈplʷaje], Sardinian_C [PROI], Spanish [ˈʎuβja]

**Cognate Set "289":** Czech [PRSETI], Slovak [PRSAT]

**Cognate Set "210":** Czech [dɛːʃtʲ], Polish [dɛʃʧ], Russian [doʒdʲ], Slovenian [DEZ], Ukrainian [(PADE)_DOSC]

**Cognate Set "1961":** Danish [ʁɑjn], Dutch_List [ˈreɣənə(n)], English [reɪn], Faroese [REGNA], Frisian [rain], German [ˈre:gnən], Icelandic_ST [ˈrɪkna], Norwegian [REGNE], Swedish [rɛŋn]

****Cognate Set "562":** Digor_Ossetic [war-], Kurdish [baran], Pashto [BARAN_UREZI], Persian [BARIDAN], Hindi [bɑrɪʃ], Urdu [bəɾsɑt̪]

- - 1. Basic Concept: "red"

**Cognate Set "491":** Greek_Mod [ˈko̞ciˌno̞s], Welsh_N [koːx]

**Cognate Set "2972":** Catalan [bəɾˈmɛʎ], Portuguese_ST [vɨɾˈmɐjʎu]

**Cognate Set "2866":** Hindi [la:l], Urdu [lɑl]

**Cognate Set "1969":** Slovenian [RUDJCE], Breton_List [RUZ], Catalan [rɔtʃ], Danish [ʁoð], Dutch_List [rot], English [rɛd], Faroese [REYDUR], French [ʀuʒ], Frisian [rɪ.ə], German [ro:t], Icelandic_ST [ˈrøyːðʏr], Irish_A [rūad], Italian [ˈrosso], Lithuanian_ST [rɐʊˈdo:nɐs], Norwegian [ɾøː], Provencal [JO], Provencal [ROUGE], Sardinian_C [ARRUBIU], Spanish [ˈroxo], Swedish [røːd], Welsh_N [RHUDD]

**Cognate Set "163":** Bulgarian [t͡ʃɛˈrvɛn], Byelorussian [CYRVONY], Czech [tʃɛrvɛniː], Polish [ʧɛrˈvɔnɨ], Serbocroatian [CRVEN], Slovak [CERVENY], Ukrainian [CERVONYJ]

****Cognate Set "3043":** Digor_Ossetic [surχ], Kurdish [sor], Pashto [SUR], Persian [SORKH], Urdu [sʊɾχ]

- - 1. Basic Concept: "right"

**Cognate Set "42":** Breton_List [GWIRION], Welsh_N [CYWIR]

**Cognate Set "2867":** Hindi [THIK], Urdu [ʈʰik]

**Cognate Set "211":** Byelorussian [PRAVIDLOVY], Czech [spraːvniː], Russian [ˈpravilʲnɨj], Serbocroatian [ISPRAVNO], Slovak [SPRAVNY], Slovenian [PROV], Ukrainian [PRJAMYJ]

**Cognate Set "2044":** Catalan [ʒus], French [ʒyst], Italian [ˈdʒusto], Provencal [JUST], Sardinian_C [GUSTU]

**Cognate Set "2042":** Urdu [d̪ɾust], Digor_Ossetic [rast]

**Cognate Set "1977":** Rumanian_List [DREPT], Catalan [dɾɛt], Catalan [rɛktə], Portuguese_ST [diˈɾɐjtu], Spanish [DERECHO], Danish [ʁad̥ʰ], Dutch_List [rɛχt], Dutch_List [rɛχtˈmatəɣ], English [raɪt], Faroese [RAETTUR], German [rɛçt], Icelandic_ST [ˈrjɛhtʏr], Norwegian [RIKTIG], Swedish [rɛt], Swedish [rɪktɪɡ]

***Cognate Set "2045":** Rumanian_List [corect], Catalan [kuˈrektə], Portuguese_ST [kuˈʀɛtu], Spanish [koˈrekto]

- - 1. Basic Concept: "rightside"

**Cognate Set "327":** Danish [ˈhʌjʁʌ], Faroese [HOGRI], Icelandic_ST [HAEGRI], Norwegian [HOYRE], Swedish [ˈhø:gər]

**Cognate Set "2649":** Albanian_Standard [i_djathtë], Breton_List [DEHOU], Bulgarian [DJASNO], Greek_Mod [DHEKSIS], Greek_Mod [ðe̞ˌk͡siˈa], Irish_A [DEAS], Italian [ˈdɛstro], Lithuanian_ST [DESINYS], Serbocroatian [DESNICA], Slovenian [DESNA_RAKA], Welsh_N [DE]

**Cognate Set "239":** Byelorussian [PRAVY], Czech [praviː], Polish [ˈpravɨ], Russian [ˈpravɨj], Slovak [PRAVY], Ukrainian [PRAVORUC]

**Cognate Set "2049":** Hindi [DAYA], Urdu [d̪ɑjɑ̃]

**Cognate Set "1986":** Catalan [dɾɛt], French [dʀwɑ], Portuguese_ST [diˈɾejtɐ], Provencal [DRE], Rumanian_List [(PE_PARTA)_DREAPTA_(ON_THE_RIGHT_SIDE)], Rumanian_List [drept], Sardinian_C [DERETTU], Spanish [deˈɾeʧa], Persian [RAST], Dutch_List [rɛχts], English [raɪt], German [rɛçt]

- - 1. Basic Concept: "river"

**Cognate Set "872":** Norwegian [elv], Swedish [ælv]

**Cognate Set "653":** Latvian [upe], Lithuanian_ST [ʊpʲe:], Irish_A [ABHAINN], Welsh_N [AFON]

**Cognate Set "1903":** German [flʊs], Danish [floð], Swedish [flu:d]

**Cognate Set "1902":** Italian [ˈfjume], Rumanian_List [FLUVIU], Sardinian_C [FLUMINI]

**Cognate Set "1900":** Faroese [ɔaː], Icelandic_ST [auː]

**Cognate Set "164":** Catalan [ˈriw], Portuguese_ST [ˈʀiu], Provencal [ˈrriw], Rumanian_List [rɨw], Spanish [ˈrio], Bulgarian [rɛˈka], Byelorussian [RAKA], Czech [r̝ɛka], Polish [ˈʒɛka], Russian [rʲeˈka], Serbocroatian [REKA], Slovak [RIEKA], Ukrainian [RICKA]

***Cognate Set "2973":** French [ʀivjɛʀ], Provencal [RIBIERO], Frisian [rəˈvi.ər], Dutch_List [riˈvir], English [ˈrɪvə]

- - 1. Basic Concept: "road"

**Cognate Set "731":** Latvian [cel_̜š], Lithuanian_ST [‘kælʲɐs]

**Cognate Set "44":** Breton_List [HENT], Welsh_N [HEOL]

**Cognate Set "2974":** Catalan [kəˈmi], French [ʃ(ə)mɛ̃], Spanish [kaˈmino]

**Cognate Set "290":** Czech [tsɛsta], Slovak [CESTA], Slovenian [CESTA]

**Cognate Set "2869":** Hindi [SEREK], Urdu [səɽək]

**Cognate Set "2445":** Kurdish [rê], Pashto [LAR], Persian [RAH]

**Cognate Set "240":** Polish [ˈdrɔɡa], Russian [ˈdoraga], Ukrainian [DOROHA]

**Cognate Set "1999":** Danish [vɑj], Dutch_List [wɛχ], Faroese [ˈveːvʊr], German [vɛk], Icelandic_ST [vegr], Norwegian [VEI], Swedish [vɛ:g]

**Cognate Set "1119":** Hindi [RASTA], Urdu [ɾɑst̪ə]

**Cognate Set "1118":** Byelorussian [SLJAX], Ukrainian [SLJAX]

***Cognate Set "688":** Italian [ˈstrada], Portuguese_ST [ɨʃˈtɾadɐ], German [ˈlantʃtra:sə]

***Cognate Set "494":** Rumanian_List [drum], Greek_Mod [ˈðro̞mo̞s]

- - 1. Basic Concept: "root"

**Cognate Set "732":** Latvian [sakne], Lithuanian_ST [SAKNIS]

**Cognate Set "165":** Bulgarian [ˈkɔrɛn], Byelorussian [KARINˈ], Czech [kor̝ɛn], Polish [ˈkɔʒɛɲ], Russian [ˈkorʲenʲ], Serbocroatian [KOREN], Slovak [KOREN], Slovenian [KORERUKA], Ukrainian [KORINˈ]

****Cognate Set "2004":** Breton_List [GWRIZIENN], Catalan [əˈrɛl], Danish [ʁoð], Dutch_List [ˈwɔrtəl], Faroese [rɔut], French [ʀasin], Frisian [ˈvuatəl], German [ˈvʊt͜sl̩], Greek_Mod [ˈriza], Icelandic_ST [ROT], Irish_A [PREAMH], Italian [raˈditʃe], Kurdish [reh], Norwegian [ROT], Pashto [RISA], Persian [riʃe], Portuguese_ST [ɾɐˈiʃ], Provencal [RACINO], Rumanian_List [rădăcină], Sardinian_C [ARREZINA], Spanish [raˈiθ], Swedish [ru:t], Welsh_N [GWREIDDYN], Albanian_Standard [rrënjë], English [ru:t]

- - 1. Basic Concept: "rope"

**Cognate Set "213":** Bulgarian [vɤ̞ˈʒɛ], Byelorussian [VJAROWKA], Latvian [vìrve], Lithuanian_ST [VIRVE], Russian [vʲeˈrʲovka], Ukrainian [virjovka]

**Cognate Set "2053":** Czech [provas], Slovak [POVRAZ]

**Cognate Set "2051":** Hindi [RESSA], Urdu [ɾəsːə]

***Cognate Set "328":** Danish [ʁεb̥], Faroese [REIP], Icelandic_ST [REIPI], Swedish [re:p], English [rəʊp], Welsh_N [RHAFF], Irish_A [ROPA]

***Cognate Set "2975":** Breton_List [KORDENN], Catalan [ˈkuɾðiʎ], Catalan [ˈkɔɾðə], French [kɔʀd], Italian [ˈkɔrda], Portuguese_ST [ˈkɔɾðɐ], Provencal [CORDO], Spanish [ˈkweɾða], Dutch_List [kort]

***Cognate Set "2057":** Polish [ˈʎina], Ukrainian [LYNVA], Swedish [ˈli:na]

***Cognate Set "2054":** Ukrainian [SNUR], German [ʃnuːr], Polish [ʃnur]

***Cognate Set "2014":** Latvian [taũva], Dutch_List [tɑu], Faroese [to:], Frisian [tɔ.u], German [tau], Norwegian [TAU]

- - 1. Basic Concept: "rotten"

**Cognate Set "2060":** Armenian_Mod [pʰəˈtɑʦ], Catalan [puðɾit], Digor_Ossetic [ɐmbud], Faroese [FUGVIN], French [puʀi], German [fa‿ul], Icelandic_ST [FUINN], Latvian [sapuvis], Lithuanian_ST [SUPUVES], Persian [PUSIDE], Portuguese_ST [ˈpodɾɨ], Provencal [POURRI], Rumanian_List [putred], Spanish [poðˈɾiðo]

**Cognate Set "166":** Bulgarian [gnil], Byelorussian [HNILY], Czech [ZETLELY], Czech [sxɲɪliː], Polish [ˈzɡɲiwɨ], Russian [gniˈloj], Serbocroatian [GNJIO], Slovak [ZHNITY], Ukrainian [HNYLYJ]

****Cognate Set "2020":** Danish [ˈʁʌðən], Dutch_List [vəˈrɔt], Faroese [ROTIN], Frisian [rɔtsjə], Norwegian [ROTTEN], Swedish [ˈrɵtən], English [ˈrɒtən]

- - 1. Basic Concept: "round"

**Cognate Set "1774":** Hindi [ɡɔl], Urdu [ɡol]

**Cognate Set "1772":** Latvian [apaļš], Lithuanian_ST [apvalus]

**Cognate Set "1771":** Welsh_N [crwn], Bulgarian [krəgəl], Czech [okroʊ̯ɦliː], Polish [ɔˈkrɔ̃ɡwɨ], Russian [ˈkruglij], Serbocroatian [okrugao]

***Cognate Set "1770":** French [ʀɔ̃], Italian [ˈtondo], Rumanian_List [rotund], Spanish [reˈðondo], Danish [ʁɔn], Dutch_List [rɔnt], English [raʊnd], German [rʊnt], Norwegian [rund], Swedish [rɵnd]

- - 1. Basic Concept: "rub"

**Cognate Set "596":** Hindi [REGERNA], Urdu [ɾəɡəɽnə]

**Cognate Set "329":** Danish [ˈg̥ni:ðə], Faroese [ˈɡnʊdʒa], Icelandic_ST [NUA], Norwegian [GNI], Swedish [ˈgni:da], Swedish [ˈgnɵga]

**Cognate Set "2802":** Pashto [MUSEL], Persian [MALIDAN]

**Cognate Set "2065":** Armenian_Mod [K`OREL], Welsh_N [CRAFU]

**Cognate Set "2028":** Dutch_List [ˈvrɛivə(n)], Frisian [ˈvrjo.uə], German [ˈra‿ibn̩]

**Cognate Set "167":** Greek_Mod [ˈtrivo̞], Bulgarian [ˈtɤ̞rkɐm], Byelorussian [CERCI], Czech [tr̝̊iːt], Lithuanian_ST [TRINTI], Polish [ˈtʃɛʨ], Russian [ˈtʲerʲetʲ], Serbocroatian [TRENJE], Slovak [TRET], Ukrainian [TERTJA]

***Cognate Set "2976":** Albanian_Standard [fërkoj], Latvian [beȓž], Catalan [frəˈɣa], Italian [freˈgare], Portuguese_ST [ɨʃfɾɨˈɡaɾ], Rumanian_List [a_freˈka], Sardinian_C [FRIGAI]

***Cognate Set "2064":** Welsh_N [RHWBIO], English [rʌb]

****Cognate Set "194":** French [fʀɔte], Provencal [FRETA], Spanish [fɾoˈtaɾ]

- - 1. Basic Concept: "salt"

**Cognate Set "844":** Hindi [NEMEK], Pashto [MALGA], Persian [NAMAK], Urdu [nəmək]

**Cognate Set "2035":** Armenian_Mod [ɑʁ], Breton_List [HOLEN], Bulgarian [sɔl], Byelorussian [SOLˈ], Catalan [sal], Czech [suːl], Danish [sald̥ʰ], Dutch_List [zɑut], English [sɔ:lt], Faroese [sal̬t], French [sɛl], Frisian [sɔ:t], German [zalt͜s], Greek_Mod [aˈlati], Icelandic_ST [sal̥t], Irish_A [SALANN], Italian [ˈsale], Latvian [sā̀ls], Norwegian [SALT], Polish [sul], Portuguese_ST [sal], Provencal [SAU], Rumanian_List [sare], Russian [solʲ], Sardinian_C [SALI], Serbocroatian [SOL], Slovak [SOL], Slovenian [SOV], Spanish [sal], Swedish [salt], Ukrainian [SILˈ], Welsh_N [HALEN]

- - 1. Basic Concept: "sand"

**Cognate Set "733":** Latvian [smìlts], Lithuanian_ST [SMELIS]

**Cognate Set "2041":** Armenian_Mod [ɑˈvɑz], Danish [san], Dutch_List [zɑnt], English [sænd], Faroese [SANDUR], French [sɑbl], Frisian [sɔ:n], German [sant], Greek_Mod [ˈamo̞s], Icelandic_ST [ˈsantʏr], Italian [ˈsabbja], Norwegian [SAND], Provencal [ˈsaβlo], Swedish [sand]

**Cognate Set "168":** Bulgarian [ˈpjasɤk], Byelorussian [PJASOK], Czech [piːsɛk], Polish [ˈpʲasɛk], Russian [pʲeˈsok], Serbocroatian [PESAK], Slovak [PIESOK], Slovenian [PESK], Ukrainian [PISOK]

**Cognate Set "1127":** Kurdish [rik], Pashto [RIG]

**Cognate Set "1123":** Sardinian_C [ARENA], Portuguese_ST [ɐˈɾejɐ], Spanish [aˈɾena]

***Cognate Set "2977":** Albanian_Standard [rërë], Italian [ˈrena]

- - 1. Basic Concept: "say"

**Cognate Set "564":** Hindi [kəhna], Urdu [kɛhnə]

**Cognate Set "2978":** Catalan [di], French [diʀ], Italian [ˈdire], Portuguese_ST [diˈzeɾ], Provencal [DIRE], Rumanian_List [a_ziˈtʃe], Spanish [deˈθiɾ]

**Cognate Set "2048":** Latvian [saka], Lithuanian_ST [sɐˈkʲi:tʲɪ], Danish [si:], Dutch_List [ˈzɛɣə(n)], English [seɪ], Frisian [ˈsɪzə], German [ˈza:gn̩], Icelandic_ST [ˈseiːja], Norwegian [SI], Swedish [ˈsɛja]

**Cognate Set "169":** Slovenian [RECI], Czech [r̝iːkat], Slovak [RIEKNUT]

**Cognate Set "1190":** Bulgarian [ˈkazvɐm], Byelorussian [SKAZACˈ], Russian [skaˈzatʲ], Serbocroatian [KAZATI], Ukrainian [SKAZATY]

**Cognate Set "1130":** Slovak [HOVORIT], Russian [govoˈritʲ], Ukrainian [HOOORYTY]

- - 1. Basic Concept: "scratch"

**Cognate Set "866":** Polish [ˈdrapaʨ], Ukrainian [DRJAPADY]

**Cognate Set "50":** Armenian_Mod [kʰɛɾˈʦɛl], Bulgarian [ˈt͡ʃɛʃɐ], Byelorussian [CYXACCA], Greek_Mod [ˈk͡sino̞], Hindi [KHEROCNA], Latvian [kasa], Lithuanian_ST [KASYTI], Russian [tʃeˈsatʲsʲa], Serbocroatian [CESATI], Urdu [kʊɾəʧnə]

**Cognate Set "373":** Faroese [KLORA], Icelandic_ST [ˈkʰlouːra], Norwegian [KLO], Swedish [ˈklø:sa]

**Cognate Set "271":** Catalan [gɾəˈta], French [gʀate], Italian [gratˈtare], Provencal [GRATA]

**Cognate Set "2595":** Albanian_Standard [kruaj], Danish [ˈg̥ʰʁɑsə], English [skrætʃ], German [ˈkrat͜sn̩]

**Cognate Set "2069":** Portuguese_ST [ʀɐʃˈpaɾ], Spanish [rasˈkaɾ]

**Cognate Set "2066":** Frisian [skrab], Lithuanian_ST [KRAPSTYTI], Rumanian_List [A_SCARPINA], Sardinian_C [SKRAFFI], Armenian_Mod [CANKREL], Armenian_Mod [kʰoˈɾɛl], Breton_List [KRAVAT], Welsh_N [CRAFU], Czech [ʃkraːbat], Slovak [SKRABAT]

- - 1. Basic Concept: "sea"

**Cognate Set "734":** Latvian [jũra], Lithuanian_ST [‘ju:rɐ]

**Cognate Set "2805":** Hindi [SEMUDR], Urdu [səmənd̪əɾ]

**Cognate Set "2596":** Greek_Mod [ˈθalaˌsa], Albanian_Standard [det]

**Cognate Set "2061":** German [me:ɐ̯], Breton_List [MOR], Welsh_N [MOR], Catalan [mar], French [mɛʀ], Italian [ˈmare], Portuguese_ST [maɾ], Provencal [MAR], Rumanian_List [ˈmare], Sardinian_C [MARI], Spanish [maɾ], Bulgarian [moˈrɛ], Byelorussian [MORA], Czech [mor̝ɛ], Polish [ˈmɔʒɛ], Russian [ˈmorʲe], Serbocroatian [MORE], Slovak [MORE], Slovenian [MORJE], Ukrainian [MORE]

**Cognate Set "1989":** Danish [hɑw], Faroese [HAV], Swedish [hɑːv]

**Cognate Set "1988":** Dutch_List [ze], English [si:], Faroese [ˈʃɛɡvʊr], Frisian [seː], German [ze:], Icelandic_ST [SAER], Norwegian [SJO], Swedish [ɧøː]

***Cognate Set "1991":** Persian [OQYANUS], German [ˈo:t͜sea:n]

- - 1. Basic Concept: "see"

**Cognate Set "849":** Kurdish [dîtin], Persian [DIDAN]

**Cognate Set "51":** Breton_List [GWELOUT], Welsh_N [GWELD]

**Cognate Set "2980":** Catalan [ˈbɛwɾə], French [vwaʀ], Italian [veˈdere], Portuguese_ST [veɾ], Provencal [VEIRE], Rumanian_List [a_veˈde̯a], Sardinian_C [BIRI], Spanish [beɾ], Bulgarian [ˈviʒdɐm], Czech [vɪɟɛt], Polish [ˈvʲiʥɛʨ], Russian [ˈvidʲetʲ], Serbocroatian [VIDETI], Slovak [VIDET], Slovenian [VIDIS], Ukrainian [DYVYTYSˈ]

**Cognate Set "2873":** Hindi [dekʰna], Urdu [d̪ekʰnə]

**Cognate Set "2067":** Albanian_Standard [shoh], Danish [se], Dutch_List [zin], English [si:], Faroese [ˈsʊdʒːa], Frisian [sjɛn], German [ˈze:ən], Icelandic_ST [ˈsjauː], Norwegian [SE], Swedish [se:]

- - 1. Basic Concept: "seed"

**Cognate Set "850":** Kurdish [tov], Persian [toxm]

**Cognate Set "498":** Greek_Mod [ˈspo̞ro̞s], Albanian_Standard [farë]

**Cognate Set "374":** Danish [frø], Faroese [FRAE], Icelandic_ST [fraiː], Norwegian [FRO]

**Cognate Set "2806":** Hindi [BIJ], Kurdish [bizir], Urdu [biʤ]

**Cognate Set "273":** Catalan [gɾə], Provencal [GRANO]

**Cognate Set "2074":** Armenian_Mod [seɾm], Breton_List [HAD], Bulgarian [ZERNO], Byelorussian [NASENˈNE], Czech [ZRNO], Czech [sɛmɛno], Danish [sεð], Dutch_List [zat], English [si:d], French [s(ə)mɑ̃s], Frisian [si.ə(t)], German [za:t], Irish_A [SIOL], Italian [ˈseme], Latvian [sę̃kla], Lithuanian_ST [‘sʲe:klɐ], Polish [ˈʑarnɔ], Portuguese_ST [sɨˈmẽtɨ], Rumanian_List [səˈmɨnʦə], Russian [ˈzʲerno], Sardinian_C [SEMINI], Serbocroatian [SEME], Slovak [SEMENO], Slovenian [SEME], Spanish [seˈmiʎa], Swedish [sɛ:d], Ukrainian [NASINNJA], Welsh_N [HEDYN]

- - 1. Basic Concept: "sew"

**Cognate Set "52":** Breton_List [GWRAIT], Welsh_N [GWNIO]

**Cognate Set "2874":** Bulgarian [ˈʃijɐ], Byelorussian [SYCˈ], Catalan [kuˈzi], Czech [ʃiːt], Danish [sy], English [səʊ], Faroese [SEYMA], French [kudʀ], Hindi [SINA], Icelandic_ST [SAUMA], Italian [kuˈtʃire], Latvian [šuj], Lithuanian_ST [‘sʲu:tʲɪ], Norwegian [SY], Polish [ʃɨʨ], Portuguese_ST [kuˈzeɾ], Provencal [COURDURA], Rumanian_List [a_coase], Russian [ʃɨtʲ], Sardinian_C [KUSIRI], Serbocroatian [SITI], Slovak [SIT], Slovenian [SIVAT], Spanish [koˈseɾ], Swedish [sy:], Ukrainian [SYTY], Urdu [sinə]

**Cognate Set "2078":** Dutch_List [ˈnajə(n)], Frisian [NAEIJE], German [ˈnɛ:ən]

- - 1. Basic Concept: "sharp"

**Cognate Set "401":** French [tʀɑ̃ʃɑ̃], Provencal [TRANCHU]

**Cognate Set "375":** Faroese [ˈkvasːʊr], Swedish [vas]

**Cognate Set "274":** Italian [taʎˈʎɛnte], Provencal [TAIU], Rumanian_List [TAIOS]

**Cognate Set "2598":** Albanian_Standard [i_mprehtë], Portuguese_ST [ɐfiˈadu], Portuguese_ST [ɐɡuˈsadu], Rumanian_List [ascuțit], Sardinian_C [AKKUCCU], Spanish [afiˈlaðo], Spanish [aˈɣuðo], Bulgarian [ˈɔstɤr], Byelorussian [VOSTRY], Czech [ostriː], Latvian [ass], Lithuanian_ST [ASTRUS], Polish [ˈɔstrɨ], Russian [ˈostrɨj], Serbocroatian [OSTAR], Slovak [OSTRY], Slovenian [OSTER], Ukrainian [HOCTRYJ]

**Cognate Set "2085":** Danish [sg̥ʰɑ:b̥], Dutch_List [sχɛrp], English [ʃɑ:p], Frisian [skɛrp], German [ʃarf], Norwegian [SKARP], Swedish [skarp]

**Cognate Set "2070":** Faroese [BEITUR], Icelandic_ST [ˈpeihtʏr]

**Cognate Set "159":** Digor_Ossetic [čirʁ], Hindi [TEJ], Pashto [TERE], Persian [TIZ], Urdu [t̪ez]

- - 1. Basic Concept: "short"

**Cognate Set "597":** Hindi [CHOTA], Urdu [ˈʧʰo.ʈə]

**Cognate Set "54":** Breton_List [BERR], Welsh_N [BYR]

**Cognate Set "376":** Faroese [STUTTUR], Icelandic_ST [ˈstʏhtʏr]

**Cognate Set "2091":** Danish [g̥ʰɒ:d̥ʰ], Dutch_List [kɔrt], Frisian [ku̯at], German [kʊrt͜s], Norwegian [KORT], Swedish [kɔrt]

**Cognate Set "2071":** Portuguese_ST [BREVE], Provencal [BREU]

***Cognate Set "2729":** English [ʃɔ:t], Armenian_Mod [kɑɾʧ], Byelorussian [KAROTKI], Catalan [kuɾ], Czech [kraːtkiː], French [kuʀ], Italian [ˈkorto], Polish [ˈkrutkʲi], Portuguese_ST [ˈkuɾtu], Rumanian_List [scurt], Russian [koˈrotkɨj], Sardinian_C [KURCU], Serbocroatian [KRATAK], Slovak [KRATKY], Slovenian [KRATKO], Spanish [ˈkoɾto], Ukrainian [KOROTKYJ], Albanian_Standard [i_skhurtër]

- - 1. Basic Concept: "sing"

**Cognate Set "2875":** Hindi [ɡa:na], Urdu [ɡɑnə]

**Cognate Set "2096":** Danish [ˈsøŋə], Dutch_List [ˈzɪŋə(n)], English [sɪŋ], Faroese [SYNGJA], Frisian [ˈsjoŋə], German [ˈzɪŋən], Icelandic_ST [SYNGVA], Norwegian [SYNGE], Swedish [ˈʃɵŋa]

**Cognate Set "171":** Bulgarian [ˈpɛjɐ], Byelorussian [PJAJACˈ], Czech [spiːvat], Latvian [dziêd], Polish [ˈɕpʲɛvaʨ], Russian [pʲetʲ], Serbocroatian [PEVATI], Slovak [SPIEVAT], Slovenian [PETI], Ukrainian [SPIVATY]

***Cognate Set "2659":** Albanian_Standard [këndoj], Breton_List [KANA], Welsh_N [kænɪi], French [ʃɑ̃te], Italian [kanˈtare], Portuguese_ST [kɐ̃ˈtaɾ], Provencal [CANTA], Rumanian_List [a_kɨnˈta], Sardinian_C [KANTAI], Spanish [kanˈtaɾ]

- - 1. Basic Concept: "sit"

**Cognate Set "853":** Kurdish [danîşîn], Kurdish [rûniştin], Persian [NESHASTAN]

**Cognate Set "598":** Digor_Ossetic [bad-], Hindi [bɛʈʰn̪a:], Urdu [bɛʈʰnə]

**Cognate Set "2103":** Armenian_Mod [nəsˈtɛl], Breton_List [AZEZA], Bulgarian [sɛˈdjɐ], Byelorussian [SJADZECˈ], Catalan [əsənˈta], Czech [sɛɟɛt], Danish [ˈseðə], Dutch_List [ˈzɪtə(n)], English [sɪt], Faroese [SITA], French [aswaʀ], Frisian [SITTE], German [ˈsɪt͜sn̩], Icelandic_ST [ˈsɪːtja], Irish_A [SUIDHE], Italian [seˈdere], Latvian [sę̂d], Lithuanian_ST [sʲe:’dʲe:tʲɪ], Norwegian [SITTE], Polish [ˈɕɛʥɛʨ], Portuguese_ST [sẽˈtaɾse], Portuguese_ST [ɨʃˈtaɾ_sẽˈtadu], Provencal [ASSETA], Rumanian_List [a_şedea], Russian [siˈdʲetʲ], Sardinian_C [SI_SEZZI], Serbocroatian [SEDITI], Slovak [SEDET], Slovenian [SEDET], Spanish [senˈtaɾse], Swedish [ˈsita], Ukrainian [SYDITY], Welsh_N [EISTEDD]

- - 1. Basic Concept: "skin"

**Cognate Set "735":** Latvian [âda], Lithuanian_ST [ODA]

**Cognate Set "55":** Breton_List [KROCˈHEN], Irish_A [CRAICEANN], Welsh_N [CROEN]

**Cognate Set "2109":** Lithuanian_ST [plėnė̃], German [ha‿ut], Catalan [peʎ], French [po], Italian [ˈpɛlle], Portuguese_ST [ˈpɛlɨ], Provencal [PEAU], Provencal [pɛl], Rumanian_List [ˈpjele], Sardinian_C [PEDDI], Spanish [pjel]

**Cognate Set "203":** Greek_Mod [pe̞ˈʦi], Pashto [POST], Persian [pust]

**Cognate Set "172":** Bulgarian [ˈkɔʒɐ], Czech [kuːʒɛ], Russian [koʒa], Serbocroatian [KOZA], Slovak [KOZA], Slovenian [KOZA]

***Cognate Set "2877":** Digor_Ossetic [car], Hindi [CAM], Kurdish [çerm], Urdu [kʰɑl], Danish [huð], Dutch_List [hœyt], Faroese [HUO], Frisian [hu:t], Icelandic_ST [HUO], Norwegian [hud], Swedish [hʉ:d], Byelorussian [SKURA], Polish [ˈskura], Ukrainian [SKURA], Spanish [CUTIS]

***Cognate Set "1449":** English [skɪn], Faroese [SKINN], Icelandic_ST [SKINN], Swedish [ʃin]

- - 1. Basic Concept: "sky"

**Cognate Set "214":** Hindi [ASMAN], Pashto [ASMAN], Persian [ASEMAN], Urdu [ʔɑsmɑn]

**Cognate Set "2114":** Danish [ˈheməl], Dutch_List [ˈhɛməl], Faroese [ˈhɪmːal], Frisian [ˈhiməl], German [ˈhɪml̩], Icelandic_ST [HIMINN], Norwegian [HIMMEL], Swedish [ˈhiməl]

**Cognate Set "173":** Bulgarian [nɛˈbɛ], Byelorussian [NEBA], Czech [nɛbɛ], Latvian [debess], Polish [ˈɲɛbɔ], Russian [ˈnʲebo], Serbocroatian [NEBO], Slovak [NEBO], Slovenian [NEBO], Ukrainian [NEBO]

***Cognate Set "2982":** Albanian_Standard [qiell], Catalan [sɛl], French [sjɛl], Italian [ˈtʃɛlo], Portuguese_ST [sɛw], Provencal [CEU], Rumanian_List [ʧer], Sardinian_C [CELU], Spanish [ˈθjelo]

- - 1. Basic Concept: "sleep"

**Cognate Set "779":** Latvian [mieguot], Lithuanian_ST [MIEGOTI]

**Cognate Set "56":** Breton_List [KOUSKET], Welsh_N [CYSGU]

**Cognate Set "2983":** Catalan [duɾˈmi], French [doʀmiʀ], Italian [dorˈmire], Portuguese_ST [duɾˈmiɾ], Provencal [DOURMI], Rumanian_List [a_dorˈmi], Sardinian_C [DORMIRI], Spanish [doɾˈmiɾ]

**Cognate Set "2732":** Armenian_Mod [kʰəˈnɛl], Breton_List [HUNIA], Bulgarian [spjɐ], Byelorussian [SPACˈ], Czech [spaːt], Danish [ˈsɒwə], Dutch_List [ˈslapə(n)], English [sli:p], Faroese [SOVA], Frisian [ˈsli.əpə], German [ʃla:fn̩], Hindi [SONA], Icelandic_ST [ˈsɔːva], Kurdish [xewtin], Norwegian [SOVE], Persian [KHABIDAN], Polish [spaʨ], Russian [spatʲ], Serbocroatian [SPAVATI], Slovak [SPAT], Slovenian [SPATI], Swedish [ˈso:va], Ukrainian [SPATY], Ukrainian [ZASYPLJATY], Urdu [sonə]

- - 1. Basic Concept: "small"

**Cognate Set "736":** Latvian [mazs], Lithuanian_ST [‘ma:ʒɐs]

**Cognate Set "689":** Italian [ˈpikkolo], Provencal [PICHOUN]

**Cognate Set "57":** Breton_List [BIHAN], Irish_A [BEAG], Welsh_N [BACH]

**Cognate Set "2984":** Portuguese_ST [pɨˈkenu], Spanish [peˈkeɲo]

**Cognate Set "2879":** Hindi [tʃʰoˈʈa], Urdu [ˈʧʰo.ʈə]

**Cognate Set "2808":** Kurdish [biçûk], Pashto [KUCNAJ], Persian [kʰuːtʃʰekʲ]

**Cognate Set "275":** Catalan [pəˈtit], French [pəti], Provencal [PICHOT], Sardinian_C [PITTIKKU]

**Cognate Set "2128":** Dutch_List [klɛin], Frisian [kli.ən], German [kla‿in]

**Cognate Set "2077":** Danish [ˈlilə], Faroese [LITIL], Icelandic_ST [ˈliːtɪtl], Norwegian [LITEN], Swedish [ˈliːtɛn]

**Cognate Set "174":** English [smɔ:l], Bulgarian [ˈmalɤk], Byelorussian [MALY], Czech [maliː], Polish [ˈmawɨ], Russian [ˈmalʲenʲkɨj], Serbocroatian [MALI], Slovak [MALY], Slovenian [MAJKEN], Ukrainian [MALYJ]

***Cognate Set "507":** Rumanian_List [mik], Faroese [SMAUR], Greek_Mod [miˈkro̞s]

- - 1. Basic Concept: "smell"

**Cognate Set "599":** Hindi [SUGHNA], Urdu [sũɡʰnə]

**Cognate Set "59":** Breton_List [KLEVOUT], Welsh_N [AROGLEUO]

**Cognate Set "377":** Faroese [TEVJA], Icelandic_ST [THEFA]

**Cognate Set "330":** Danish [ˈlɔg̥d̥ʰə], Norwegian [LUKTE], Swedish [ˈlɵkta]

**Cognate Set "2985":** Sardinian_C [FRAGAI], Portuguese_ST [ʃejˈɾaɾ]

**Cognate Set "2733":** Armenian_Mod [hot_əzˈgɑl], Latvian [ôž], Lithuanian_ST [UZUOSTI], Catalan [uluˈra], Italian [odoˈrare], Spanish [oˈleɾ]

**Cognate Set "243":** Slovak [NUCHAT], Slovak [VONAT], Russian [nʲuxatʲ], Ukrainian [NJUXATY]

**Cognate Set "2139":** Dutch_List [ˈrœykə(n)], German [ˈri:çn̩]

**Cognate Set "2081":** Byelorussian [CUCˈ_PAX], Czech [tʃɪxat], Polish [ʧuʨ]

**Cognate Set "2079":** French [sɑ̃tiʀ], Italian [senˈtire], Provencal [SENTI]

***Cognate Set "508":** Bulgarian [miˈriʃɐ], Serbocroatian [MIRISATI], Slovenian [SMRDI], Rumanian_List [a_mirosi], Greek_Mod [miˈrizo̞ˌme̞], Latvian [smiȓd]

- - 1. Basic Concept: "smoke"

**Cognate Set "2734":** English [sməʊk], Breton_List [MOGED], Welsh_N [MWG]

**Cognate Set "241":** Byelorussian [DYM], Catalan [fum], Czech [KOUR], Czech [diːm], French [fyme], Hindi [DHUA], Irish_A [DEATACH_(TOIT_U.)], Italian [ˈfumo], Kurdish [dûkel], Kurdish [dûxan], Latvian [dũmi], Lithuanian_ST [DUMAI], Pashto [DUD], Persian [DUD], Polish [dɨm], Portuguese_ST [ˈfumu], Provencal [FUM], Rumanian_List [fum], Russian [dɨm], Sardinian_C [FUMU], Serbocroatian [DIM], Slovak [DYM], Slovenian [DIM], Spanish [ˈumo], Ukrainian [DYM], Urdu [d̪ʰʊwɑ̃]

**Cognate Set "2148":** Danish [ʁʌj], Dutch_List [rok], Faroese [ˈrɔikʊr], Frisian [re:k], German [ra‿ux], Icelandic_ST [ˈreiːkʏr], Norwegian [røyk_(røk)], Swedish [rø:k]

- - 1. Basic Concept: "smooth"

**Cognate Set "2641":** Catalan [ʎis], French [lis], Italian [ˈliʃʃo], Portuguese_ST [ˈlizu], Sardinian_C [LISU], Spanish [ˈliso]

**Cognate Set "251":** Pashto [SAF], Persian [SAF]

**Cognate Set "2155":** Danish [g̥lad̥ʰ], Dutch_List [ɣəˈlɛik], Frisian [GLAD], German [glat], Norwegian [GLATT], Swedish [glat], Bulgarian [ˈɡladɤk], Byelorussian [HLADKI], Czech [ɦlatkiː], Latvian [gludęns], Polish [ˈɡwatki], Russian [ˈgladkɨj], Serbocroatian [GLADAK], Slovak [HLADKY], Slovenian [GLADKO], Ukrainian [HLADKYJ]

**Cognate Set "2090":** Italian [ˈpjano], Provencal [PLANIE]

**Cognate Set "2087":** Digor_Ossetic [liʁz], Irish_A [SLEAMHAIN], Welsh_N [LLYFN], Faroese [ˈslatːʊr], Icelandic_ST [SLETTR], Swedish [slɛːt]

***Cognate Set "2086":** Pashto [HAVAR], Urdu [həmwɑɾ]

- - 1. Basic Concept: "snake"

**Cognate Set "60":** Breton_List [AER_(NAER)], Welsh_N [NEIDR]

**Cognate Set "379":** Faroese [(HOGG)ORMUR], Icelandic_ST [ˈɔrmʏr], Norwegian [ORM], Swedish [ʊrm]

**Cognate Set "357":** English [sneɪk], Icelandic_ST [ˈstnauːkʏr]

**Cognate Set "2986":** Portuguese_ST [ˈkɔβɾɐ], Spanish [CULEBRA]

**Cognate Set "292":** Ukrainian [HADJUKA], Czech [ɦat], Slovak [HAD]

**Cognate Set "2880":** Albanian_Standard [gjarpër], Hindi [sɑ̃ːp], Urdu [sɑ̃p], Catalan [seɾp], French [sɛʀpɑ̃], Italian [ˈsɛrpe], Portuguese_ST [sɨɾˈpẽtɨ], Provencal [SERP], Rumanian_List [ˈʃarpe], Sardinian_C [SERPENTI], Spanish [seɾˈpjente]

**Cognate Set "2735":** Greek_Mod [ˈfiði], Polish [vɔ̃ʒ], Armenian_Mod [oʦʰ]

**Cognate Set "261":** Pashto [MAR], Persian [mɒːɾ]

**Cognate Set "175":** Bulgarian [zmiˈja], Byelorussian [ZˈMJAJA], Russian [ˈzmʲeja], Serbocroatian [ZMIJA], Ukrainian [zmija]

****Cognate Set "2161":** Dutch_List [slɑŋ], Faroese [ALANGA], Frisian [ˈslaŋ(ə)], German [ˈʃlaŋə], Icelandic_ST [ˈstlauŋka], Danish [ˈslɑŋə]

- - 1. Basic Concept: "snow"

**Cognate Set "61":** Breton_List [ERCˈH], Welsh_N [EIRA]

**Cognate Set "2736":** Armenian_Mod [ʣɪ̯un], Greek_Mod [ˈço̞ni]

**Cognate Set "272":** Hindi [BERPH], Pashto [VAVRA], Persian [BARF], Urdu [bəɾf]

**Cognate Set "2165":** Bulgarian [ˈsnjak], Byelorussian [SˈNEH], Catalan [new], Czech [sniːx], Danish [sne], Dutch_List [snew], English [snəʊ], French [nɛʒ], Frisian [sni.ə], German [ʃne:], Icelandic_ST [SNJOR], Irish_A [SNEACHTA], Italian [ˈneve], Latvian [snìegs], Lithuanian_ST [sʲnʲjæɡɐs], Norwegian [SNE], Polish [ɕɲɛk], Portuguese_ST [ˈnɛvɨ], Provencal [nɛw], Russian [snʲeg], Sardinian_C [NI], Serbocroatian [SNEG], Slovak [SNEH], Slovenian [SNEK], Spanish [ˈnjeβe], Swedish [snøː], Ukrainian [SNIH]

***Cognate Set "2605":** Rumanian_List [zəˈpadə], Albanian_Standard [borë]

- - 1. Basic Concept: "some"

**Cognate Set "283":** Bulgarian [ˈŋʲakolko], Byelorussian [NEKATORY], Czech [NEKTERY], Czech [ɲɛkolɪk], Russian [nʲeˈmnogo], Serbocroatian [NEKI], Slovak [NIEKTORY]

**Cognate Set "2172":** Danish [ˈnoən], Faroese [ˈnakrɪr], Icelandic_ST [NOKKRIR], Norwegian [NOEN], Swedish [ˈnoːˌgra]

**Cognate Set "2101":** Dutch_List [ˈenəχ], Catalan [əl’ɣun], Italian [alˈkuni], Portuguese_ST [aɫˈɣũʃ], Spanish [alˈɣunos]

**Cognate Set "2100":** Dutch_List [ˈsɔməɣə], English [sʌm]

**Cognate Set "2098":** Polish [ˈtrɔxɛ̃], Ukrainian [XTOSˈ]

**Cognate Set "2094":** Hindi [KOI], Hindi [KUCH], Urdu [kʊʧʰ], Catalan [əl’ɣun], French [kɛlk], Italian [alˈkuni], Portuguese_ST [aɫˈɣũʃ], Provencal [QUAUQUIS-UN], Spanish [alˈɣunos], Bulgarian [ˈŋʲakolko], Czech [NEKTERY], Czech [ɲɛkolɪk], Polish [ˈkʲilka], Serbocroatian [NEKI], Slovak [NIEKTORY]

- - 1. Basic Concept: "spit"

**Cognate Set "813":** Digor_Ossetic [tu_kɐn-_/_liχstɐ_kɐn-_(spit.PL_do)], Persian [TOF_KARDAN]

**Cognate Set "694":** Hindi [KHUKNA], Urdu [t̪ʰuknə]

**Cognate Set "2104":** Provencal [SALIVA], Irish_A [SEILE_DO_CHAITHEAMH]

***Cognate Set "402":** Breton_List [KRANCHAT], French [kʀaʃe]

****Cognate Set "2179":** Albanian_Standard [pështyj], Armenian_Mod [T`UK`], Armenian_Mod [tʰəˈkʰɛl], Bulgarian [ˈpʎʲujɐ], Byelorussian [PLERACˈ], Catalan [əskuˈpi], Czech [plɪvat], Danish [ˈsb̥_ød̥ʰə], Dutch_List [ˈspywə(n)], English [spɪt], Faroese [SPYTA], Frisian [ˈspɔ.i̯ə], German [ˈʃpe‿iən], Greek_Mod [ˈftino̞], Icelandic_ST [SPYTA], Italian [spuˈtare], Latvian [spl_̜-aũj], Lithuanian_ST [SPIAUTI], Norwegian [SPYTTE], Polish [ˈpluʨ], Portuguese_ST [kuʃˈpiɾ], Provencal [ESCUPI], Rumanian_List [a_scuipa], Russian [plʲeˈvatʲ], Sardinian_C [SKUPPI], Serbocroatian [PLJUVATI], Slovak [PL_UT], Slovenian [PLUNI], Spanish [eskuˈpiɾ], Swedish [ˈspɔta], Ukrainian [PLJUVATY], Breton_List [SKOPA]

- - 1. Basic Concept: "split"

**Cognate Set "63":** Latvian [skal_̂-da], Lithuanian_ST [SKELTI], Breton_List [FAOUTA], Irish_A [SCOILTEADH], Welsh_N [HOLLTI]

**Cognate Set "331":** Danish [g̥ʰlɒw], Faroese [KLUGVA], Icelandic_ST [KLJUFA], Norwegian [KLOVE]

**Cognate Set "2987":** French [fɑ̃dʀ], Italian [ˈfɛndere], Portuguese_ST [FENDER], Provencal [FENDRE], Spanish [HENDER]

**Cognate Set "244":** Byelorussian [RAZSCAPLJACˈ], Czech [ROZSTIPNOUTI], Czech [STIPATI], Czech [rozɟɛlɪt], Polish [rɔzˈʃʧɛpʲiʨ], Slovak [STIEPAT]

**Cognate Set "2111":** Italian [spakˈkare], Provencal [ESPECA]

**Cognate Set "2110":** Portuguese_ST [ʀɐˈʃaɾ], Spanish [RAJAR]

**Cognate Set "2108":** Russian [rasˈkalɨvatʲ], Serbocroatian [KALATI]

**Cognate Set "2107":** Frisian [ˈspjɔltə], German [ˈʃpaltn̩]

**Cognate Set "2106":** Hindi [CIRNA], Urdu [do_ʈʊkɽe_kəɾnə]

**Cognate Set "2105":** Greek_Mod [ˈscizo̞], Bulgarian [ˈt͡sɛpjɐ], Slovenian [RASCEPAJ]

****Cognate Set "2187":** Dutch_List [ˈsplɛitə(n)], Frisian [ˈsplɪtsə], Swedish [ˈsplitra], English [splɪt]

- - 1. Basic Concept: "squeeze"

**Cognate Set "874":** Norwegian [KLEMME], Swedish [KLAMMA]

**Cognate Set "403":** French [sɛʀe], Provencal [SARRA]

**Cognate Set "380":** Faroese [ˈkrɔista], Icelandic_ST [ˈkʰreista]

**Cognate Set "332":** Danish [ˈd̥ʰʁœg̥ʰə], German [ˈdrʏkn̩]

**Cognate Set "2988":** Portuguese_ST [ɐpɨɾˈtaɾ], Spanish [apɾeˈtaɾ]

**Cognate Set "2192":** Dutch_List [ˈpɛrsə(n)], Catalan [kumpɾiˈmi], Italian [ˈprɛmere], Portuguese_ST [COMPRIMIR], Provencal [PRESSA], Sardinian_C [SPREMI], Spanish [COMORIMIR]

**Cognate Set "2115":** Catalan [əˈstɾɛɲə], Spanish [ESTRECHAR]

**Cognate Set "176":** Bulgarian [ˈstiskɐm], Czech [STISKNOUTI], Czech [ZMACKNOUTI], Slovak [TISNUT], Slovenian [STISNI], Ukrainian [TYSNUTY]

- - 1. Basic Concept: "stab"

**Cognate Set "738":** Latvian [durt], Lithuanian_ST [DURTI]

**Cognate Set "381":** Faroese [STINGA], Icelandic_ST [ˈstiŋka]

**Cognate Set "2989":** Catalan [əpunɲəˈla], French [pwaɲaʀde], Portuguese_ST [ɐpuɲɐˈlaɾ], Provencal [POUGNARDA], Sardinian_C [PUNNALAI], Spanish [apuɲaˈlaɾ]

**Cognate Set "256":** Czech [klati], Polish [kwuʨ], Russian [kalɔtʲ], Slovak [klatʲ], Ukrainian [KOLOTY]

**Cognate Set "2197":** Danish [ˈsd̥ʰeg̥ʰə], Dutch_List [stekə(n)], Frisian [ˈstikjə], German [ɛɐ̯ˈʃtɛçn̩], Norwegian [STIKKE], Swedish [ˈstika]

**Cognate Set "216":** Bulgarian [boda], Serbocroatian [UBOSTI]

**Cognate Set "2119":** Czech [piːxnoʊ̯t], Slovak [PICHAT]

- - 1. Basic Concept: "stand"

**Cognate Set "869":** Sardinian_C [ATTURAI], Portuguese_ST [ɨʃˈtaɾ_ɐ̃j_pɛ], Spanish [esˈtaɾ_de_pje]

**Cognate Set "404":** French [ɛtʀ], Provencal [ARRESTA/ESTRE_DEBOUT]

**Cognate Set "2202":** Breton_List [WAR_ZAV], Bulgarian [stoˈjɐ], Byelorussian [STAMCˈ], Czech [staːt], Danish [sd̥ʰɔ], Dutch_List [stan], English [stænd], Faroese [STANDA], Frisian [stɪ.ən], German [ˈʃte:ən], Greek_Mod [ˈste̞ko̞ˌme̞], Icelandic_ST [ˈstanta], Irish_A [SEASAMH], Italian [ˈstare_im_ˈpjɛdi], Kurdish [westîn], Latvian [stãv], Lithuanian_ST [STOVETI], Norwegian [STA], Persian [iːstʰɒːˈdæn], Polish [staʨ], Rumanian_List [a_sta_în_picioare], Russian [stoˈjatʲ], Serbocroatian [STAJATI], Slovak [STAT], Slovenian [STAJ], Swedish [sto:], Ukrainian [STOJATY], Welsh_N [SEFYLL]

- - 1. Basic Concept: "star"

**Cognate Set "323":** Armenian_Mod [ˈɑstəʁ], Breton_List [STERED], Catalan [əsˈtɛl], Danish [ˈsd̥ʰjæɐ̯nə], Digor_Ossetic [stˈalu], Dutch_List [stɛr], English [stɑ:], Faroese [ˈʃœdna], French [etwal], Frisian [stjɛ:r], German [ʃtɛrn], Greek_Mod [ˈastro̞], Hindi [TARA], Icelandic_ST [ˈstjartna], Italian [ˈstella], Kurdish [stêr], Norwegian [STJERNE], Pashto [STORAJ], Persian [SETARE], Portuguese_ST [ɨʃˈtɾelɐ], Provencal [ESTELLO], Rumanian_List [stea], Sardinian_C [STELLA], Spanish [esˈtɾeʎa], Swedish [ˈɧɛːrna], Urdu [t̪ɑɾə], Welsh_N [ˈsɛrɛn]

**Cognate Set "245":** Byelorussian [ZORKA], Ukrainian [ZIRKA]

**Cognate Set "177":** Bulgarian [zvɛzˈda], Czech [ɦvjɛzda], Latvian [zvàigzne], Lithuanian_ST [ʒʋɐjɡʲʒʲˈdʲe:], Polish [ˈɡvʲazda], Russian [zvʲezˈda], Serbocroatian [ZVEZDA], Slovak [HVIEZDA], Slovenian [ZVEZDO]

- - 1. Basic Concept: "stick"

**Cognate Set "870":** Portuguese_ST [PAO], Spanish [ˈpalo]

**Cognate Set "601":** Hindi [DENDA], Urdu [ɖənɖɑ]

**Cognate Set "405":** Breton_List [BAZ], French [bɑtɔ̃], Italian [basˈtone], Portuguese_ST [bɐʃˈtɐ̃ũ], Provencal [BASTOUN], Rumanian_List [băț], Sardinian_C [BASTONI]

**Cognate Set "246":** Byelorussian [KIJ], Polish [kʲij], Ukrainian [KYJOK]

**Cognate Set "2210":** Danish [sd̥ʰʌg̥ʰ], Dutch_List [stɔk], Frisian [stɔ:k], German [ʃtɔk], Norwegian [STOKK]

**Cognate Set "217":** Russian [ˈpalka], Slovenian [PALCA]

**Cognate Set "178":** Bulgarian [ˈprɤ̞t͡ʃkɐ], Slovak [PRUT]

***Cognate Set "2122":** Serbocroatian [STAP], Faroese [ˈstɛavʊr], Icelandic_ST [staːvʏr]

- - 1. Basic Concept: "stone"

**Cognate Set "2990":** Catalan [ˈpeðɾə], French [pjɛʀ], Italian [ˈpjɛtra], Portuguese_ST [ˈpɛdɾɐ], Provencal [ˈpɛjɾo], Rumanian_List [piatră], Sardinian_C [PERDA], Spanish [ˈpjeðɾa]

**Cognate Set "2884":** Hindi [PETTHER], Urdu [pət̪ʰːəɾ]

**Cognate Set "2216":** Danish [sd̥ʰen], Dutch_List [sten], English [stəʊn], Faroese [STEINUR], Frisian [sti.ən], German [ʃta‿in], Icelandic_ST [steitn], Norwegian [sten], Swedish [ste:n]

**Cognate Set "179":** Persian [SANG], Bulgarian [ˈkamɤk], Byelorussian [KAMENˈ], Czech [kaːmɛn], Latvian [akmens], Lithuanian_ST [AKMUO], Polish [ˈkamʲɛɲ], Russian [ˈkamʲenʲ], Serbocroatian [KAMEN], Slovak [KAMEN], Slovenian [KAMEN], Ukrainian [KAMINˈ]

- - 1. Basic Concept: "straight"

**Cognate Set "740":** Latvian [tàisns], Lithuanian_ST [TIESUS]

**Cognate Set "566":** Hindi [SIDHA], Urdu [sid̪ʰə]

**Cognate Set "294":** Czech [pr̝̊iːmiː], Russian [prʲaˈmoj], Slovak [priamy], Ukrainian [PRJAMYJ]

**Cognate Set "247":** Byelorussian [PROSTY], Polish [ˈprɔstɨ]

**Cognate Set "2224":** Catalan [dɾɛt], Catalan [ˈrɛktə], Digor_Ossetic [rast], Dutch_List [rɛχt], Faroese [RAETTUR], French [dʀwɑ], Frisian [rjoxt], Icelandic_ST [ˈrjɛhtʏr], Irish_A [DIREACH], Italian [diˈritto], Norwegian [RETT], Persian [RAST], Portuguese_ST [diˈɾɐjtu], Provencal [DRE], Rumanian_List [drept], Sardinian_C [DERETTU], Spanish [DERECHO], Spanish [ˈrekto], Swedish [rɑ:k], Swedish [rɛ:t]

**Cognate Set "2124":** Faroese [BEINUR], Icelandic_ST [peitn]

**Cognate Set "180":** Bulgarian [praf], Serbocroatian [USPRAVAN]

- - 1. Basic Concept: "suck"

**Cognate Set "512":** Greek_Mod [ro̞ˈfo̞], Pashto [RUDEL]

**Cognate Set "2885":** Hindi [CUSNA], Urdu [ʧusnə]

**Cognate Set "2231":** Breton_List [SUN], Bulgarian [ˈsut͡ʃɐ], Byelorussian [SSACˈ], Czech [saːt], Danish [ˈsu:ə], Dutch_List [ˈzœyɣə(n)], English [sʌk], Faroese [SUGVA], French [syse], Frisian [ˈsu:gə], German [ˈza‿ugn̩], Icelandic_ST [SJUGA], Italian [sukˈkjare], Norwegian [SU], Polish [ˈssaʨ], Portuguese_ST [suˈɡaɾ], Provencal [SUCA], Rumanian_List [a_ˈsudʒe], Russian [soˈsatʲ], Sardinian_C [SUCCAI], Serbocroatian [SISATI], Slovak [SAT], Slovenian [SOSAT], Swedish [ˈsʉ:ga], Ukrainian [SMOKTATY], Welsh_N [SUGNO]

**Cognate Set "2129":** Catalan [ʃukˈɫa], Portuguese_ST [CHUCHAR], Provencal [CHUCHA]

**Cognate Set "2127":** Portuguese_ST [ʃuˈpaɾ], Spanish [ʧuˈpaɾ]

- - 1. Basic Concept: "sun"

**Cognate Set "2238":** Breton_List [HEOL], Bulgarian [ˈslɤ̞nt͡sɛ], Byelorussian [SONCA], Catalan [sɔɫ], Czech [slʊntsɛ], Danish [sol], Digor_Ossetic [χor], Dutch_List [zɔn], English [sʌn], Faroese [sɔuːl], French [sɔlɛj], Frisian [SINNE], German [ˈzɔnə], Greek_Mod [ˈiʎo̞s], Hindi [su:ɾədʒ], Icelandic_ST [souːl], Italian [ˈsole], Kurdish [xor], Latvian [saũle], Lithuanian_ST [sɑule:], Norwegian [suːl], Persian [KHORSHID], Polish [ˈswɔɲʦɛ], Portuguese_ST [sɔl], Provencal [suˈlel], Rumanian_List [ˈsware], Russian [ˈsolntse], Sardinian_C [SOLI], Serbocroatian [SUNCE], Slovak [SLNKO], Slovenian [SUNCE], Spanish [sol], Swedish [suːl], Ukrainian [SONCE], Urdu [suɾəʤ], Welsh_N [haɨl]

- - 1. Basic Concept: "swell"

**Cognate Set "741":** Serbocroatian [OTECI], Slovenian [ZETEKLU]

**Cognate Set "66":** Breton_List [CˈHOUEZA], Welsh_N [CHWYDDO]

**Cognate Set "382":** Faroese [TRUTNA], Icelandic_ST [BOLGNA], Frisian [FORBOLGEN]

**Cognate Set "2245":** Danish [ˈsvulmə], Dutch_List [ˈzwɛlə(n)], English [swɛl], German [ˈʃvɛlən], Swedish [ˈpø:sa], Swedish [ˈsvala]

**Cognate Set "2142":** Catalan [inˈfla], French [ɑ̃fle], Italian [gonˈfjare], Portuguese_ST [ĩˈʃaɾ], Provencal [BOUFIGA], Provencal [ENFLA], Provencal [GOUNFLA], Rumanian_List [a_se_umfla], Sardinian_C [UNFRAI], Spanish [inˈʧaɾse]

**Cognate Set "2134":** Greek_Mod [fuˈsko̞no̞], Byelorussian [PUXNUCˈ], Czech [opʊxnoʊ̯t], Lithuanian_ST [PUSTI], Polish [ˈpuxnɔ̃ʨ], Russian [ˈpuxnutʲ], Slovak [PUCHNUT], Ukrainian [PUXNUTY]

**Cognate Set "2132":** Urdu [suʤnə], Digor_Ossetic [rɐsuj-]

- - 1. Basic Concept: "swim"

**Cognate Set "567":** Hindi [TERNA], Urdu [t̪ɛɾnə]

**Cognate Set "2248":** Danish [ˈsvœmə], Dutch_List [ˈzwɛmə(n)], English [swɪm], Faroese [SVIMJA], Frisian [ˈsvɪmə], German [ˈʃvɪmən], Icelandic_ST [SYNDA], Norwegian [ˈsʋøme], Swedish [SIMMA]

**Cognate Set "181":** Bulgarian [ˈpluvɐm], Byelorussian [PLAVACˈ], Czech [plavat], Lithuanian_ST [PLAUKTI], Polish [ˈpwɨvaʨ], Russian [plɨtʲ], Serbocroatian [PLIVATI], Slovak [PLAVAT], Ukrainian [PLYVTY]

***Cognate Set "2642":** Albanian_Standard [notoj], Breton_List [NEUNVIER], Irish_A [SNAMH], Welsh_N [NOFIO], Catalan [nəˈða], French [naʒe], Italian [nwoˈtare], Portuguese_ST [nɐˈdaɾ], Provencal [NADA], Rumanian_List [a_înota], Sardinian_C [NARAI], Spanish [naˈðaɾ]

- - 1. Basic Concept: "tail"

**Cognate Set "913":** Serbocroatian [REP], Slovenian [RIPP]

**Cognate Set "515":** Irish_A [EARBALL], Greek_Mod [uˈra]

**Cognate Set "334":** Danish [ˈhɛ:lə], Faroese [HALI], Norwegian [HALE]

**Cognate Set "2993":** Catalan [ˈkua], French [kø], Italian [ˈkoda], Portuguese_ST [ˈkauðɐ], Provencal [CO], Rumanian_List [coadă], Sardinian_C [KOA], Spanish [ˈkola]

**Cognate Set "2812":** Persian [dom], Urdu [d̪ʊm]

**Cognate Set "248":** Slovak [CHVOST], Byelorussian [XVOST], Russian [xvostʲ], Ukrainian [XVIST]

**Cognate Set "2253":** Dutch_List [start], Frisian [STIRT], Swedish [ʃæʈ]

**Cognate Set "1077":** Portuguese_ST [ˈɾaβu], Spanish [RABO]

***Cognate Set "424":** German [ʃvant͜s], Swedish [svans]

- - 1. Basic Concept: "that"

**Cognate Set "2994":** Catalan [əˈkɛʃ], French [səla], Italian [kwel], Portuguese_ST [ɐˈkilu], Provencal [AQUEST], Rumanian_List [aˈʧela], Sardinian_C [KUDDU], Spanish [aˈkel]

**Cognate Set "2261":** Albanian_Standard [atë], Armenian_Mod [ɑɪ̯n], Breton_List [AN_DRA-ZE], Byelorussian [HETY], Czech [tamto], Danish [dεn], Dutch_List [di], English [ðæt], Faroese [TANN], Frisian [dɔt], German [das], Icelandic_ST [THESSI], Irish_A [SAN], Latvian [tas], Lithuanian_ST [TAS], Norwegian [den], Persian [AN_(UN)], Polish [ˈtamtɛn], Russian [tot], Slovak [TEN], Slovenian [TISTO], Swedish [ˈdɛnə], Ukrainian [TOJ], Welsh_N [HWNNW_(MASC.)]

**Cognate Set "1925":** Hindi [vo], Urdu [woʰ]

**Cognate Set "1768":** Greek_Mod [NA], German [je:nɐ]

**Cognate Set "1766":** Bulgarian [onoˈva], Czech [ONEN], Greek_Mod [e̞ˈcino̞]

**Cognate Set "1761":** Catalan [əˈkɛʃ], French [səla], Italian [kwel], Portuguese_ST [ɐˈkilu], Provencal [AQUEST], Rumanian_List [aˈʧela], Sardinian_C [KUDDU], Spanish [aˈkel]

- - 1. Basic Concept: "there"

**Cognate Set "2995":** Catalan [ə’ki], French [la], Italian [la], Portuguese_ST [ALLI], Portuguese_ST [ɐˈi], Provencal [LA], Rumanian_List [aˈkolo], Spanish [ALLA], Spanish [aˈi]

**Cognate Set "2270":** Albanian_Standard [atje], Armenian_Mod [ɑɪ̯nˈtɛʁ], Bulgarian [tam], Byelorussian [TAM], Czech [tam], Danish [dεɐ̯], Dutch_List [dar], English [ðɛə], Faroese [HAR], Frisian [dər], German [dɔrt], Icelandic_ST [THAR(NA)], Latvian [tùr], Norwegian [DER], Polish [tam], Russian [tam], Serbocroatian [TAMO], Slovak [TAM], Slovenian [TOM], Swedish [dɛːr], Ukrainian [TAM]

**Cognate Set "2153":** Hindi [VEHA], Urdu [wəhɑ̃]

- - 1. Basic Concept: "they"

**Cognate Set "2996":** French [il], Portuguese_ST [ˈelɨʃ], Provencal [ELI], Rumanian_List [ELE], Rumanian_List [jej], Spanish [ˈeʎos]

**Cognate Set "2149":** Byelorussian [JANY], Czech [oɲɪ], Polish [ˈɔni], Russian [oˈni], Serbocroatian [ONI], Slovak [ONI], Slovenian [ONI], Ukrainian [VONY]

**Cognate Set "2147":** Pashto [DUJ], Hindi [VE]

**Cognate Set "2146":** Bulgarian [tɛ], Albanian_Standard [ata_(m)], Dutch_List [zɛi], Frisian [sɛɪ], German [zi:], Greek_Mod [TUS_(ACC.)], Greek_Mod [afˈti], Irish_A [SIAD], Italian [esˈsi], Sardinian_C [ISSOS], Welsh_N [hwi]

***Cognate Set "125":** English [ðɛɪ], Danish [di], Faroese [tair], Icelandic_ST [THEIR], Norwegian [DE], Swedish [deː]

- - 1. Basic Concept: "thick"

**Cognate Set "2997":** Catalan [gɾu’ʃut], Catalan [gɾɔs], Italian [ˈgrɔsso], Portuguese_ST [GROSSO], Rumanian_List [gros], Sardinian_C [GRUSSU]

**Cognate Set "296":** Slovenian [GOSTO], Slovak [HUSTY]

**Cognate Set "2887":** Hindi [MOTA], Urdu [ˈmo.ʈə]

**Cognate Set "249":** Czech [tlʊstiː], Byelorussian [TAWSTY], Russian [ˈtolstɨj], Ukrainian [tovstij]

**Cognate Set "2280":** Breton_List [TEO], Irish_A [TIUGH], Danish [d̥ʰyg̥ʰ], Dutch_List [dɪk], English [θɪk], Faroese [TJUKKUR], Frisian [tsjok], German [dɪk], Icelandic_ST [THYKKR], Norwegian [TYKK], Swedish [ɕɔk]

**Cognate Set "2145":** Catalan [əsˈpɛs], French [epɛ], Portuguese_ST [ɨʃˈpesu], Provencal [ESPES], Spanish [ESPESO]

**Cognate Set "2137":** Polish [ˈgrubɨ], Slovak [HRUBY]

**Cognate Set "183":** Bulgarian [dɛˈbɛl], Serbocroatian [DEBEO]

- - 1. Basic Concept: "thin"

**Cognate Set "690":** Italian [sotˈtile], Rumanian_List [SUBTIRE]

**Cognate Set "406":** French [mɛ̃s], Provencal [MINCE]

**Cognate Set "2998":** Portuguese_ST [DELGADO], Spanish [delˈɣaðo]

**Cognate Set "2888":** Hindi [PETLA], Urdu [ˈpət.lə]

**Cognate Set "2289":** Breton_List [TANO], Irish_A [TANAI], Welsh_N [TENAU], Danish [d̥ʰøn], Dutch_List [dʏn], English [θɪn], Faroese [TUNNUR], Frisian [TIN], German [dʏn], Icelandic_ST [MJOR], Icelandic_ST [ˈθʏnːʏr], Norwegian [TYNN], Swedish [tɵn], Bulgarian [ˈtɤ̞nɤk], Byelorussian [CENKI], Czech [tɛŋkiː], Latvian [tiêvs], Polish [ˈʨɛ̃nkʲi], Russian [ˈtonkɨj], Serbocroatian [TANAK], Slovak [TENKY], Ukrainian [TONKYJ], Digor_Ossetic [tɐnɐg]

**Cognate Set "2159":** Sardinian_C [FINI], Portuguese_ST [ˈfinu]

**Cognate Set "2157":** Armenian_Mod [bɑˈɾɑk], Persian [BARIK]

- - 1. Basic Concept: "think"

**Cognate Set "570":** Hindi [sotʃn̪a], Urdu [soʧnə]

**Cognate Set "419":** Pashto [FIKR_KAVEL], Persian [FEKR_KARDAN]

**Cognate Set "384":** Faroese [HUGSA], Icelandic_ST [HUGSA]

**Cognate Set "2999":** Catalan [DONAR_PIENSO], Catalan [pənˈsa], French [pɑ̃se], Italian [penˈsare], Portuguese_ST [pẽˈsaɾ], Provencal [PENSA], Sardinian_C [PENSAI], Spanish [penˈsaɾ]

**Cognate Set "2299":** Danish [ˈd̥ʰεŋ_g̥ʰə], Dutch_List [ˈdɛŋkə(n)], English [θɪŋk], Frisian [ˈtɪŋkə], German [ˈdɛŋkn̩], Norwegian [TENKE], Swedish [ˈtɛŋka]

**Cognate Set "184":** Bulgarian [ˈmisljɐ], Czech [mɪslɛt], Lithuanian_ST [MASTYTI], Polish [ˈmɨɕlɛʨ], Serbocroatian [MISLITI], Slovak [MYSLET], Slovenian [ZAMISLIT], Ukrainian [MYSLYTY]

***Cognate Set "2163":** Latvian [dõmã], Byelorussian [DUMACˈ], Russian [ˈdumatʲ], Ukrainian [DUMATY]

- - 1. Basic Concept: "this"

**Cognate Set "2814":** Kurdish [ev], Persian [IN]

**Cognate Set "2309":** Armenian_Mod [ɑɪ̯s], Bulgarian [toˈva], Byelorussian [HETA], Catalan [ə’kɛt], Czech [toto], Danish [ˈd̥εnə], Dutch_List [ˈdezə], English [ðɪs], Faroese [HETTA], French [səsi], Frisian [di], German [di:s], Greek_Mod [(e̞)ˈtuto̞], Greek_Mod [afˈto̞], Icelandic_ST [ˈθɛsːɪ], Irish_A [SO], Italian [ˈkwesto], Norwegian [denne], Polish [tɛn], Portuguese_ST [ˈiʃtu], Provencal [CO], Rumanian_List [aˈʧesta], Sardinian_C [KUSTU], Slovak [TEN], Slovenian [TU], Spanish [ˈeste], Swedish [dɛn], Ukrainian [CEJ], Welsh_N [HWN_(MASC.)]

**Cognate Set "1738":** Russian [ˈetot], Hindi [jeː], Urdu [jeʰ], Catalan [ə’kɛt], French [səsi], Italian [ˈkwesto], Portuguese_ST [ˈiʃtu], Provencal [CO], Rumanian_List [aˈʧesta], Sardinian_C [KUSTU], Spanish [ˈeste]

**Cognate Set "1722":** Greek_Mod [(e̞)ˈtuto̞], Greek_Mod [afˈto̞], Serbocroatian [OVAJ]

**Cognate Set "1691":** Latvian [šis], Lithuanian_ST [SIS], Catalan [ə’kɛt], French [səsi], Italian [ˈkwesto], Portuguese_ST [ˈiʃtu], Provencal [CO], Rumanian_List [aˈʧesta], Sardinian_C [KUSTU], Spanish [ˈeste]

- - 1. Basic Concept: "thou"

**Cognate Set "436":** Albanian_Standard [ti], Armenian_Mod [du], Breton_List [TE], Bulgarian [ti], Byelorussian [TY], Catalan [tu], Czech [tɪ], Danish [du], Digor_Ossetic [du], English [ðaʊ], Faroese [tʉu], French [ty], Frisian [du], German [du:], Greek_Mod [e̞ˈsi], Hindi [t̪uː], Icelandic_ST [θu:], Irish_A [TU], Italian [tu], Kurdish [tu], Latvian [tu], Lithuanian_ST [tʊ], Norwegian [DU], Pashto [TE], Persian [t̪o], Polish [tɨ], Portuguese_ST [tu], Provencal [TU], Rumanian_List [tu], Russian [tɨ], Sardinian_C [TUI], Serbocroatian [TI], Slovak [TY], Spanish [tu], Swedish [dʉ], Ukrainian [TY], Urdu [t̪u], Welsh_N [TI]

- - 1. Basic Concept: "three"

**Cognate Set "446":** Albanian_Standard [tre], Armenian_Mod [jɛˈɾɛkʰ], Breton_List [TRI_(M)], Bulgarian [tri], Byelorussian [TRY], Catalan [trɛs], Czech [tr̝̊ɪ], Danish [d̥ʰʁε], Digor_Ossetic [ɐrtɐ], Dutch_List [dri], English [θri:], Faroese [trʊdʒɪr], French [tʀwa], Frisian [ˈtrɛi̯ə], German [dra‿i], Greek_Mod [ˈtriˌa], Hindi [t̪in], Icelandic_ST [THRIR], Irish_A [TRI], Italian [tre], Latvian [trîs], Lithuanian_ST [TRYS], Norwegian [TRE], Pashto [DRE], Persian [SE], Polish [tʃɨ], Portuguese_ST [tɾeʃ], Provencal [tres], Rumanian_List [trej], Russian [tri], Sardinian_C [TRESI], Serbocroatian [TRI], Slovak [TRI], Slovenian [TRI], Spanish [tɾes], Swedish [treː], Ukrainian [TRY], Urdu [t̝in], Welsh_N [TRI]

- - 1. Basic Concept: "throw"

**Cognate Set "69":** Breton_List [TEUREL], Welsh_N [TAFLU]

**Cognate Set "407":** French [ʒəte], Italian [dʒetˈtare], Provencal [JITA], Sardinian_C [GETTAI], Spanish [ECHAR]

**Cognate Set "335":** Danish [g̥ʰasd̥ʰə], Faroese [KASTA], Icelandic_ST [KASTA], Norwegian [KASTE], Swedish [ˈkasta]

**Cognate Set "297":** Czech [ɦaːzɛt], Slovak [DODIT_]

**Cognate Set "2889":** Hindi [PHEKNA], Urdu [pʰẽknə]

**Cognate Set "2327":** Greek_Mod [ˈrixno̞], Slovenian [VRZI], Dutch_List [ˈwɛrpə(n)], Frisian [ˈvɛrpə], German [ˈvɛrfn̩]

**Cognate Set "218":** Latvian [męt], Lithuanian_ST [MESTI]

**Cognate Set "2173":** Catalan [tiˈɾa], Portuguese_ST [ɐtiˈɾaɾ], Provencal [TRAIRE], Spanish [tiˈɾaɾ]

**Cognate Set "2169":** English [θrəʊ], Frisian [ˈdro.əi̯ə]

**Cognate Set "2167":** Albanian_Standard [hedh], Byelorussian [KIDACˈ], Russian [kidatʲ], Ukrainian [KYDATY]

***Cognate Set "2171":** Rumanian_List [LANSA], Portuguese_ST [lɐ̃ˈsaɾ]

- - 1. Basic Concept: "tie"

**Cognate Set "2620":** Albanian_Standard [lidh], Catalan [ʎiˈɣa], French [lje], Italian [leˈgare], Portuguese_ST [LIGAR], Provencal [LIA], Rumanian_List [a_lega]

**Cognate Set "250":** Byelorussian [VJAZACˈ], Czech [vaːza], Polish [ˈvʲɔ̃zaʨ], Russian [ˈvʲazatʲ], Serbocroatian [VEZATI], Slovak [VIAZAT_], Slovenian [ZVEZI], Ukrainian [ZVˈJAZUVATY]

**Cognate Set "2338":** Digor_Ossetic [bɐtt-], Hindi [BADHNA], Persian [BASTAN], Urdu [bɑ̃d̪ʰnə], Danish [ˈb̥enə], Dutch_List [ˈbɪndə(n)], Faroese [BINDA], German [ˈbɪndn̩], Icelandic_ST [ˈpɪnta], Norwegian [BINDE], Swedish [ˈbinda]

**Cognate Set "2180":** Portuguese_ST [ɐˈtaɾ], Spanish [aˈtaɾ]

- - 1. Basic Concept: "tongue"

**Cognate Set "2347":** Armenian_Mod [lɛˈzu], Breton_List [TEOD], Bulgarian [ɛˈzik], Byelorussian [JAZYK], Catalan [ˈʎɛŋɡwə], Czech [jazɪk], Danish [d̥ʰɔŋə], Digor_Ossetic [ɐvzɐg], Dutch_List [tɔŋ], English [tʌŋ], Faroese [ˈtʊŋɡa], French [lɑ̃g], Frisian [ˈtoŋə], German [ˈt͜sʊŋə], Hindi [dʒiːbʱ], Icelandic_ST [ˈtʰuŋka], Irish_A [TEANGA], Italian [ˈlingwa], Kurdish [ziman], Lithuanian_ST [LIEZUVIS], Norwegian [TUNGE], Pashto [ZEBA], Persian [zæˈbɒːn], Polish [ˈjɛ̃zɨk], Portuguese_ST [ˈlĩguɐ], Provencal [ˈleŋɡo], Rumanian_List [ˈlimbə], Russian [jaˈzɨk], Sardinian_C [LINGUA], Serbocroatian [JEZIK], Slovak [JAZYK], Slovenian [JEZIK], Spanish [ˈleŋɡwa], Swedish [ˈtɵŋa], Ukrainian [JAZYK], Urdu [zəbɑn], Welsh_N [TAFOD]

- - 1. Basic Concept: "tooth"

**Cognate Set "2622":** Bulgarian [zɤ̞p], Byelorussian [ZUB], Czech [zʊp], Latvian [zòbs], Polish [zɔ̃p], Russian [zub], Serbocroatian [ZUB], Slovak [ZUB], Slovenian [OD_NAPREJ_ZOBY], Ukrainian [ZUB], Albanian_Standard [dhëmb]

**Cognate Set "2358":** Russian [desˈna], Armenian_Mod [ɑˈtɑm], Breton_List [DANT], Catalan [den], Danish [d̥ʰan], Digor_Ossetic [dɐndag], Dutch_List [tɑnt], English [tu:θ], Faroese [tɔnː], French [dɑ̃], Frisian [tosk], German [t͜sa:n], Greek_Mod [ˈðo̞(n)di], Hindi [d̪ãːt̪], Icelandic_ST [tʰœnː], Italian [ˈdɛnte], Kurdish [didan], Kurdish [diran], Lithuanian_ST [DANTIS], Norwegian [TANN], Persian [dændɒːn], Portuguese_ST [ˈdẽtɨ], Provencal [DENT], Rumanian_List [ˈdinte], Sardinian_C [DENTI], Spanish [ˈdjente], Swedish [tand], Urdu [d̪ɑ̃t̪], Welsh_N [DANT]

- - 1. Basic Concept: "tree"

**Cognate Set "675":** Irish_A [CRANN], Welsh_N [pren]

**Cognate Set "489":** Kurdish [dar], Pashto [DIRAXT], Persian [deˈɾæxtʰ], Urdu [d̪ɾəχt]

**Cognate Set "3000":** Catalan [ˈaßɾə], French [aʀbʀ], Italian [ˈalbero], Portuguese_ST [ˈaɾvuɾɨ], Provencal [AUBRE], Rumanian_List [ˈarbore], Spanish [ˈaɾβol]

**Cognate Set "298":** Czech [strom], Slovak [STROM]

**Cognate Set "2623":** Greek_Mod [ˈðe̞n̪ðro̞], English [tri:], Danish [d̥ʰʁɛ], Faroese [trɛa], Icelandic_ST [tʰrjɛː], Norwegian [TRE], Swedish [trɛ:d], Bulgarian [dɤˈrvɔ], Byelorussian [DREVA], Latvian [darva], Lithuanian_ST [derva], Lithuanian_ST [drevė], Polish [ˈdʒɛvɔ], Russian [ˈdʲerʲevo], Serbocroatian [DRVO], Slovenian [DREV], Ukrainian [DEREVO]

**Cognate Set "2363":** Dutch_List [bom], Frisian [bɪəm], German [ba‿um]

- - 1. Basic Concept: "turn"

**Cognate Set "602":** Hindi [MURNA], Urdu [mʊɽnə]

**Cognate Set "336":** Faroese [ˈsnɪɡva], Icelandic_ST [SNUA(SK)], Norwegian [SNU]

**Cognate Set "3001":** Portuguese_ST [viˈɾaɾ], Provencal [VIRA], Rumanian_List [A_COTI], Rumanian_List [A_VIRA], Sardinian_C [FURRIAI]

**Cognate Set "2185":** Danish [ˈvεnə_sɑj], Dutch_List [ˈwɛndə(n)], German [ˈvɛndn̩], Swedish [ˈvɛ:nda]

**Cognate Set "2183":** Czech [totʃɪt], Slovak [TOCIT]

**Cognate Set "2182":** Catalan [ʒiˈɾa], Italian [dʒiˈrare], Portuguese_ST [ʒiˈɾaɾ], Spanish [xiˈɾaɾ]

**Cognate Set "186":** Spanish [VOLVER], Bulgarian [vɤ̞rˈtjɐ_sɛ], Byelorussian [PAVORACYVACˈ], Latvian [vḕrš], Russian [povoˈratʃɨvatʲ], Slovak [OBRACAT], Slovenian [ABRNI_SE], Ukrainian [OBERTATYSˈ]

***Cognate Set "276":** French [tuʀne], English [tɜ:n]

- - 1. Basic Concept: "two"

**Cognate Set "506":** Albanian_Standard [dy], Armenian_Mod [jɛɾˈku], Breton_List [DAOU_(M)], Bulgarian [dva], Byelorussian [DVA], Catalan [dos], Czech [dva], Danish [d̥ʰo], Digor_Ossetic [duwɐ], Dutch_List [twe], English [tu:], Faroese [TVEIR], French [dø], Frisian [tva:], German [t͜sva‿i], Greek_Mod [ˈðiˌo̞], Hindi [DO], Icelandic_ST [TVEIR], Irish_A [DO_(DHA)], Italian [ˈdue], Kurdish [du], Latvian [divi], Lithuanian_ST [DU], Norwegian [TO], Pashto [DVA], Persian [DO], Polish [dva], Portuguese_ST [doiʃ], Provencal [DOUS], Rumanian_List [doj], Russian [dva], Sardinian_C [DUSU], Serbocroatian [DVA], Slovak [DVA], Slovenian [DUA], Spanish [dos], Swedish [tvo:], Ukrainian [DVA], Urdu [d̪o], Welsh_N [DAU]

- - 1. Basic Concept: "vomit"

**Cognate Set "71":** Breton_List [CˈHOUEDI], Welsh_N [TAFLUIFYNY]

**Cognate Set "571":** Hindi [ULTI_KERNU], Urdu [qɛ_kəɾnə]

**Cognate Set "337":** Danish [ˈg̥ʰasd̥ʰə_ʌb̥ʰ], Icelandic_ST [KASTA_UPP], Norwegian [KASTE_OPP], Swedish [KASTA_(FA)_UPP]

**Cognate Set "2643":** Albanian_Standard [vjell], Bulgarian [pʊˈvrɤ̞ʃtɐm], Byelorussian [VYRACACˈ], Czech [zvratsɛt], Serbocroatian [POVRACATI], Ukrainian [VERTATY]

**Cognate Set "2366":** Dutch_List [ˈbrakə(n)], German [ˈa‿usbrɛçn̩]

**Cognate Set "2188":** Frisian [ˈspɔ.i̯ə], Faroese [SPYGGJA], Swedish [spy:]

**Cognate Set "187":** Russian [blʲiˈvat͡ɕ], Ukrainian [BLJUVATY]

***Cognate Set "2891":** English [ˈvɑmɪt], Greek_Mod [e̞ˈmo̞], Digor_Ossetic [wom-], Catalan [ˈbumiˈta], French [vɔmiʀ], Italian [vomiˈtare], Portuguese_ST [vumiˈtaɾ], Provencal [BOUMI], Rumanian_List [a_vomita], Sardinian_C [VOMITAI], Spanish [bomiˈtaɾ], Latvian [vemj], Lithuanian_ST [VEMTI]

- - 1. Basic Concept: "walk"

**Cognate Set "72":** Breton_List [KERZOUT], Welsh_N [CERDDED]

**Cognate Set "572":** Hindi [CELNA], Urdu [ʧəlnə]

**Cognate Set "408":** French [maʀʃe], Provencal [MARCHA]

**Cognate Set "338":** German [ˈge:ən], Danish [g̥ɔ], Faroese [GANGA], Icelandic_ST [ˈkauŋka], Norwegian [GA], Swedish [GA__TILL_FOTS], Swedish [go:]

**Cognate Set "3003":** Sardinian_C [PASSILLAI], Catalan [əˈna], Portuguese_ST [PASSEAR], Portuguese_ST [ɐ̃ˈdaɾ], Spanish [PASEAR]

**Cognate Set "2317":** Digor_Ossetic [cɐw-], Kurdish [çûn]

**Cognate Set "219":** Czech [xoɟɪt], Polish [ˈxɔʥiʨ], Russian [xoˈditʲ], Serbocroatian [SETATI], Slovak [CHODIT], Slovenian [HODIT], Ukrainian [XODYTY]

**Cognate Set "1113":** Catalan [kəmiˈna], Italian [kammiˈnare], Portuguese_ST [kɐmiˈɲaɾ], Spanish [kamiˈnaɾ]

**Cognate Set "1109":** Byelorussian [ISCI], Czech [jiːt], Latvian [iêt], Ukrainian [ITY]

- - 1. Basic Concept: "warm"

**Cognate Set "73":** Breton_List [TOMM], Welsh_N [CYNNES], Welsh_N [TEG], Bulgarian [ˈtɔpɤl], Byelorussian [CEPLY], Czech [tɛpliː], Polish [ˈʨɛpwɨ], Russian [ˈtʲoplɨj], Serbocroatian [TOPLO], Slovak [TEPLY], Ukrainian [TEPLYJ]

**Cognate Set "520":** Armenian_Mod [TOT`], Pashto [TOD]

**Cognate Set "277":** Faroese [LYGGJUR], Latvian [sìlts], Lithuanian_ST [‘ʃʲɪltɐs], Catalan [kəˈlen], French [ʃo], Italian [ˈkaldo], Provencal [CAUD], Rumanian_List [cald], Sardinian_C [KALLENTI], Spanish [ˈkaliðo]

**Cognate Set "2371":** Albanian_Standard [i_ngrohtë], Digor_Ossetic [ʁar], Hindi [GEREM], Kurdish [germ], Persian [GARM], Urdu [ɡəɾm], Danish [vɑm], Dutch_List [wɑrm], English [wɔ:m], Frisian [vaːrm], German [varm], Norwegian [VARM], Swedish [varm]

**Cognate Set "1115":** Faroese [HEITUR], Icelandic_ST [ˈheiːtʏr]

- - 1. Basic Concept: "wash"

**Cognate Set "74":** Breton_List [GWALCˈHI], Welsh_N [GOLCHI]

**Cognate Set "385":** Faroese [ˈtvɔːa], Icelandic_ST [THVO], Swedish [ˈtvɛta]

**Cognate Set "3005":** Albanian_Standard [laj], French [lave], Italian [laˈvare], Portuguese_ST [lɐˈvaɾ], Provencal [LAVA], Rumanian_List [a_spăla], Spanish [laˈβaɾ]

**Cognate Set "2892":** Hindi [DHONA], Urdu [d̪ʰonə]

**Cognate Set "2750":** Armenian_Mod [ləˈvɑl], Greek_Mod [ˈple̞no̞]

**Cognate Set "2379":** Danish [ˈvasg̥ʰə], Dutch_List [ˈwɑsə(n)], English [wɒʃ], Frisian [WAECHSE], German [ˈvaʃn̩], Norwegian [VASKE]

**Cognate Set "2194":** Latvian [mazgã], Lithuanian_ST [MAZGOTI]

**Cognate Set "188":** Bulgarian [ˈmijɐ], Byelorussian [MYCˈ], Czech [miːt], Polish [mɨʨ], Russian [mɨtʲ], Slovak [MYT], Ukrainian [MYTY]

- - 1. Basic Concept: "water"

**Cognate Set "75":** Breton_List [DOUR], Welsh_N [duːr]

**Cognate Set "538":** Kurdish [av], Pashto [OBE], Persian [AB]

**Cognate Set "3006":** Catalan [ˈajɣwə], French [o], Italian [ˈakkwa], Portuguese_ST [ˈaɡwɐ], Provencal [AIGO], Rumanian_List [ˈapə], Sardinian_C [AKKUA], Spanish [ˈaɣwa]

**Cognate Set "2893":** Hindi [pa:ni:], Urdu [pɑni]

**Cognate Set "2388":** Digor_Ossetic [don], Albanian_Standard [ujë], Bulgarian [voˈda], Byelorussian [VADA], Czech [voda], Danish [van], Dutch_List [ˈwatər], English [ˈwɔ:tə], Faroese [vatn], Frisian [ˈvɛtər], German [ˈvasɐ], Icelandic_ST [vaʰtn̥], Irish_A [UISGE], Latvian [ûdens], Lithuanian_ST [ʋɐn’dʊɔ], Norwegian [VANN], Polish [ˈvɔda], Russian [voˈda], Serbocroatian [VODA], Slovak [VODA], Slovenian [VODA], Swedish [ˈvatɛn], Ukrainian [VODA]

- - 1. Basic Concept: "we"

**Cognate Set "547":** Albanian_Standard [ne], Armenian_Mod [mɛnkʰ], Breton_List [NI], Bulgarian [ˈniɛ], Byelorussian [MY], Catalan [nuz’altrəs], Czech [mɪ], Danish [vi], Digor_Ossetic [maχ], Dutch_List [wɛi], English [wi:], Faroese [viːt], French [nu], Frisian [vɛɪ], German [vi:ɐ̯], Greek_Mod [e̞ˈmis], Hindi [ɦəm], Icelandic_ST [vɪ(ː)ð], Irish_A [SINN], Italian [NOI], Kurdish [em], Kurdish [me], Latvian [mẽs], Lithuanian_ST [mæ:s], Norwegian [VI], Pashto [MUZ], Persian [mɒː], Polish [mɨ], Portuguese_ST [ˈnɔʃ], Provencal [NOUS], Rumanian_List [noj], Russian [mɨ], Sardinian_C [NOSUS], Serbocroatian [MI], Slovak [MY], Slovenian [MI], Spanish [noˈsotɾos], Swedish [viː], Ukrainian [MY], Urdu [həm], Welsh_N [ni]

- - 1. Basic Concept: "wet"

**Cognate Set "76":** Breton_List [(TO_WET)__GLEBIA], Irish_A [FLIUCH], Welsh_N [GWLYB]

**Cognate Set "742":** Latvian [slapjš], Lithuanian_ST [SLAPIAS]

**Cognate Set "553":** Digor_Ossetic [čˈifɐ/χuluj], Pashto [XIST]

**Cognate Set "536":** Greek_Mod [iˈɣro̞s], Portuguese_ST [ˈumidu], Rumanian_List [UD]

**Cognate Set "339":** Danish [vɔˀð], English [wɛt], Faroese [VATUR], Frisian [vɪət], Icelandic_ST [votur], Icelandic_ST [ˈpløyːtʏr], Norwegian [VAT], Swedish [voːt]

**Cognate Set "3007":** French [muje], Portuguese_ST [MOLHADO], Provencal [MUIA], Spanish [moˈxaðo]

**Cognate Set "2398":** Dutch_List [nɑt], German [nas]

**Cognate Set "190":** Bulgarian [ˈmɔkɤr], Byelorussian [MOKRY], Czech [mokriː], Polish [ˈmɔkrɨ], Russian [ˈmokrɨj], Serbocroatian [MOKAR], Slovak [MOKRY], Slovenian [MAKRO], Ukrainian [MOKRYJ]

- - 1. Basic Concept: "what"

**Cognate Set "560":** Albanian_Standard [çfarë], Armenian_Mod [inʧʰ], Breton_List [PETRA], Bulgarian [kɐkˈvɔ], Byelorussian [STO], Catalan [kɛ], Czech [tso], Danish [va], Digor_Ossetic [či], Dutch_List [wɑt], English [wɒt], Faroese [kvɛat], French [kə], Frisian [vɔt], German [vas], Greek_Mod [ti], Hindi [kjaː], Icelandic_ST [xʷaːð], Irish_A [CAD], Italian [ke], Kurdish [çi], Latvian [kas], Lithuanian_ST [KAS], Norwegian [HVAD], Pashto [CE], Persian [tʃʰiː], Polish [ʦɔ], Portuguese_ST [kɨ], Provencal [QUE], Rumanian_List [ʧe], Russian [ʃto], Serbocroatian [STO], Slovak [CO], Slovenian [KAY], Spanish [ke], Swedish [vɑː(d)], Ukrainian [SCO], Urdu [kjɑ], Welsh_N [BETH]

- - 1. Basic Concept: "when"

**Cognate Set "568":** Albanian_Standard [kur], Breton_List [PEUR], Bulgarian [koˈga], Byelorussian [KALI], Catalan [kwan], Czech [ɡdɪ], Danish [vɒˈnɒ], Digor_Ossetic [kɐd/ku], Dutch_List [wɑˈner], English [wɛn], Faroese [NAER], French [kɑ̃], Frisian [HONEAR], German [van], Greek_Mod [ˈpo̞te̞], Hindi [kəb], Icelandic_ST [HVENAER], Italian [ˈkwando], Latvian [kad], Lithuanian_ST [KADA], Norwegian [nor], Pashto [KELA], Persian [kʰej], Polish [ˈkʲɛdɨ], Portuguese_ST [ˈkuɐ̃ðu], Provencal [QUAND], Rumanian_List [când], Russian [kogˈda], Sardinian_C [KANDU], Serbocroatian [KADA], Slovak [KEDY], Slovenian [KEDAJ], Spanish [ˈkwando], Swedish [næ:r], Ukrainian [KOLY], Urdu [kəb], Welsh_N [PRYD]

- - 1. Basic Concept: "where"

**Cognate Set "77":** Breton_List [PELECˈH], Welsh_N [PLE]

**Cognate Set "2408":** Albanian_Standard [ku], Bulgarian [kɤˈdɛ], Byelorussian [DZE], Catalan [on], Czech [ɡdɛ], Danish [vɒ], Digor_Ossetic [kɐmi], Dutch_List [war], English [wɛə], Faroese [HVAR], French [u], German [vo:], Greek_Mod [pu], Hindi [KEHA], Icelandic_ST [kʰvaːr], Italian [ˈdove], Latvian [kùr], Lithuanian_ST [kʊr], Norwegian [HVOR], Persian [kʰoˈdʒɒː], Polish [ɡʥɛ], Portuguese_ST [ˈõðɨ], Provencal [OUNTE], Rumanian_List [INCOTRO], Rumanian_List [ˈunde], Russian [gdʲe], Sardinian_C [AUNDI], Serbocroatian [GDE], Slovak [KDE], Slovenian [KJE], Spanish [ˈdonde], Swedish [vɑːɾ], Ukrainian [DE_KUDY], Urdu [kəhɑ̃]

- - 1. Basic Concept: "white"

**Cognate Set "78":** Breton_List [GWENN], Welsh_N [GWYN]

**Cognate Set "3008":** Rumanian_List [alb], Portuguese_ST [ALVO]

**Cognate Set "2414":** Kurdish [spî], Pashto [SPIN], Persian [SEFID], Danish [við], Dutch_List [wɪt], English [waɪt], Faroese [ˈkvʊitʊr], Frisian [vit], German [va‿is], Icelandic_ST [ˈxʷiːtʏr], Norwegian [HVIT], Swedish [vi:t]

**Cognate Set "192":** Catalan [blaŋ], French [blɑ̃], Italian [ˈbjanko], Portuguese_ST [ˈbɾɐ̃ku], Provencal [BLANC], Sardinian_C [BIANKU], Spanish [ˈblaŋko], Bulgarian [bjal], Byelorussian [BELY], Czech [biːliː], Latvian [bal_̃-ts], Lithuanian_ST [‘ba:ltɐs], Polish [ˈbʲawɨ], Russian [ˈbʲelɨj], Serbocroatian [BELO], Slovak [BIELY], Ukrainian [BILYJ]

- - 1. Basic Concept: "who"

**Cognate Set "2416":** Albanian_Standard [kush], Armenian_Mod [ov], Breton_List [PIOU], Bulgarian [kɔj], Byelorussian [XTO], Catalan [ki], Czech [gdo], Danish [vεm], Digor_Ossetic [ka], Dutch_List [wi], English [hu:], Faroese [kvøːr], French [ki], Frisian [HWA], German [ve:ɐ̯], Greek_Mod [pço̞s], Hindi [KON], Icelandic_ST [xʷɛːr], Irish_A [CIA], Italian [ki], Kurdish [kî], Latvian [kas], Lithuanian_ST [KAS], Norwegian [HVEM], Pashto [COK], Persian [kʰiː], Polish [ktɔ], Portuguese_ST [kɐ̃ĩ], Provencal [QUE], Rumanian_List [cine], Russian [kto], Sardinian_C [KINI], Serbocroatian [TKO], Slovak [KTO], Slovenian [KEDU], Spanish [kjen], Swedish [vem], Ukrainian [XTO], Urdu [kɔn], Welsh_N [PWY]

- - 1. Basic Concept: "wide"

**Cognate Set "583":** Pashto [PRAX], Persian [PAHN]

**Cognate Set "2895":** Hindi [CORA], Urdu [ˈʧɔ.ɽə]

**Cognate Set "278":** Catalan [ˈamplə], Spanish [ˈanʧo]

**Cognate Set "2755":** Armenian_Mod [lɑɪ̯n], Breton_List [LEDAN], Greek_Mod [plaˈtis], Irish_A [LEATHAN], Latvian [plats], Lithuanian_ST [PLATUS], Welsh_N [ˈɬədan]

**Cognate Set "2420":** Dutch_List [wɛit], English [waɪd], Faroese [VIDUR], Frisian [vi:t], Icelandic_ST [VIOR], Norwegian [VID], Swedish [viːd]

**Cognate Set "2198":** Danish [b̥ʁεð], Dutch_List [breed], English [broad], Faroese [ˈbraijʊr], German [bra‿it], Icelandic_ST [ˈpreiːðʏr], Norwegian [BRED]

**Cognate Set "193":** Bulgarian [ʃiˈrɔk], Byelorussian [SYROKI], Czech [ʃɪrokiː], Polish [ʃɛˈrɔki], Russian [ʃɨˈrokɨj], Serbocroatian [SIROK], Slovak [SIROKY], Slovenian [SROKO], Ukrainian [SYROKYJ]

***Cognate Set "3009":** Breton_List [LARK], French [laʀʒ], Italian [ˈlargo], Portuguese_ST [ˈlaɾɣu], Provencal [LARG], Rumanian_List [larg], Sardinian_C [LARGU]

- - 1. Basic Concept: "wife"

**Cognate Set "79":** Breton_List [GWREG], Welsh_N [GWRAIG]

**Cognate Set "3010":** Catalan [muˈʎe], Italian [ˈmoʎʎe], Portuguese_ST [muˈʎɛɾ], Provencal [MOUIE], Sardinian_C [MULLERI], Spanish [muˈxeɾ]

**Cognate Set "2756":** Armenian_Mod [kin], Byelorussian [ZONKA], Czech [manʒɛlka], Danish [ˈg̥ʰo:nə], Faroese [KONA], Greek_Mod [ʝiˈne̞ka], Icelandic_ST [ˈeiːjɪn.kʰɔːna], Irish_A [BEAN], Norwegian [KONE], Persian [ZAN], Polish [ˈʒɔna], Russian [ʒeˈna], Serbocroatian [ZENA], Slovak [MANZELKA], Slovak [ZENA], Slovenian [ZENA], Ukrainian [ZINKA]

**Cognate Set "2208":** French [epuz], Italian [ˈspoza], Portuguese_ST [ɨʃˈpozɐ], Spanish [esˈposa]

**Cognate Set "2205":** Dutch_List [vrɑu], Frisian [frɔ.u̯], German [fra‿u]

**Cognate Set "2201":** Bulgarian [sɤˈpruɡɐ], Serbocroatian [SUPRUGA]

**Cognate Set "127":** Dutch_List [ˈhœysfrɑu], Faroese [ˈhʉusfrʉu]

- - 1. Basic Concept: "wind"

**Cognate Set "705":** Hindi [HEVA], Urdu [həwɑ]

**Cognate Set "594":** Pashto [BAD], Persian [BAD]

**Cognate Set "2423":** Breton_List [AVEL], Bulgarian [ˈvjatɤr], Byelorussian [VECER], Catalan [ben], Czech [viːtr̩], Danish [ven], Dutch_List [wɪnt], English [wɪnd], Faroese [VINDUR], French [vɑ̃], Frisian [vin], German [vɪnt], Hindi [VAYU], Icelandic_ST [ˈvɪntʏr], Italian [ˈvɛnto], Latvian [vẽjš], Lithuanian_ST [‘ʋʲe:jɛs], Norwegian [ʋin], Polish [vʲatr], Portuguese_ST [ˈvẽtu], Provencal [VENT], Rumanian_List [vânt], Russian [ˈvʲetʲer], Sardinian_C [BENTU], Serbocroatian [VETAR], Slovak [VIETOR], Spanish [ˈbjento], Swedish [vɪnd], Ukrainian [VITER], Welsh_N [GWYNT]

**Cognate Set "2212":** Catalan [ˈaijrə], Provencal [AURO], Greek_Mod [aˈʝe̞ras]

- - 1. Basic Concept: "wing"

**Cognate Set "526":** Armenian_Mod [tʰɛv], Greek_Mod [fte̞ˈruɣa], Latvian [spā̀rns], Lithuanian_ST [SPARNAS], Persian [PAR], Urdu [pəɾ], Welsh_N [ADAIN]

**Cognate Set "2425":** Dutch_List [ˈvløɣəl], German [ˈflʏ:gl̩]

**Cognate Set "2214":** Digor_Ossetic [bazur], Pashto [VAZAR]

**Cognate Set "195":** Bulgarian [kriˈlɔ], Byelorussian [KRYLO], Czech [kr̝̊ɪːdlo], Polish [ˈskʃɨdwɔ], Russian [ˈkrɨlo], Serbocroatian [KRILO], Slovak [KRIDLO], Slovenian [KRELUTA], Ukrainian [KRYLO]

***Cognate Set "340":** English [wɪŋ], Danish [ˈveŋə], Faroese [VONGUR], Icelandic_ST [ˈvaiŋkʏr], Norwegian [VINGE], Swedish [ˈvɪŋɛ]

***Cognate Set "3011":** Catalan [ˈaɫə], French [ɛl], Italian [ˈala], Portuguese_ST [ˈazɐ], Provencal [ALO], Sardinian_C [ALA], Spanish [ˈala], Breton_List [ASKELL]

- - 1. Basic Concept: "wipe"

**Cognate Set "80":** Breton_List [TORCHA], Welsh_N [SYCHU]

**Cognate Set "797":** Serbocroatian [OBRISAC], Slovenian [ABRESI]

**Cognate Set "743":** Latvian [slaũka], Lithuanian_ST [SLUOSTYTI]

**Cognate Set "341":** Danish [ˈd̥ʰɶɐ̯ʌ], Faroese [TURKA], Icelandic_ST [THURRKA], Norwegian [TORKE], Swedish [ˈtɔrka]

**Cognate Set "3012":** Portuguese_ST [ALIMPAR], Spanish [limˈpjaɾ]

**Cognate Set "2429":** Dutch_List [ˈveɣə(n)], Frisian [ˈfai̯ə]

**Cognate Set "2219":** Catalan [əʃuˈɣa], French [ɛsɥije], Portuguese_ST [ẽʃuˈɡaɾ], Provencal [ejsyˈɣa]

**Cognate Set "196":** Bulgarian [iztˈrivɐm], Byelorussian [VYCIRACˈ], Czech [vɪciːrat], Polish [ˈɕʨɛraʨ], Russian [ˈvɨtiratʲ], Slovak [UTRET], Ukrainian [ZYRATY]

- - 1. Basic Concept: "with"

**Cognate Set "619":** Digor_Ossetic [χɐccɐ], Hindi [SATH], Hindi [se], Urdu [sɑt̪ʰ], Bulgarian [s], Byelorussian [Z], Czech [s], Lithuanian_ST [sʊ], Polish [z], Russian [s], Serbocroatian [SA], Slovak [S], Slovenian [ZMONO], Ukrainian [Z]

**Cognate Set "363":** Faroese [viː], English [wɪð]

**Cognate Set "3014":** Breton_List [GANT], Italian [kon], Portuguese_ST [kõ], Rumanian_List [ku], Sardinian_C [KUN], Spanish [kon]

**Cognate Set "279":** Catalan [əmb], French [avɛk]

**Cognate Set "2648":** Albanian_Standard [me], Greek_Mod [me̞], Danish [með], Dutch_List [mɛt], Frisian [mai̯], German [mɪt], Icelandic_ST [MEO], Norwegian [MED], Swedish [meː(d)]

- - 1. Basic Concept: "woman"

**Cognate Set "3015":** Rumanian_List [muˈjere], Portuguese_ST [muˈʎɛɾ], Spanish [muˈxeɾ]

**Cognate Set "280":** Sardinian_C [FEMMINA], French [fam], Provencal [ˈfenno]

**Cognate Set "2759":** Armenian_Mod [kin], Bulgarian [ʒɛˈna], Byelorussian [ZANCYNA], Czech [ʒɛna], Danish [ˈg̥ʰvenə], Faroese [KONA], Greek_Mod [ʝiˈne̞ka], Icelandic_ST [ˈkʰɔːna], Irish_A [BEAN], Kurdish [jin], Norwegian [kvinne], Persian [ZAN], Russian [ˈʒenʃtʃɨna], Serbocroatian [ZENA], Slovak [ZENA], Slovenian [ZENSKA], Swedish [ˈkvɪnːa], Ukrainian [ZINKA], Welsh_N [DYNES], Welsh_N [GWRAIG]

**Cognate Set "2434":** Dutch_List [vrɑu], German [fra‿u]

**Cognate Set "1352":** Catalan [ˈdɔnə], Italian [ˈdɔnna], Norwegian [DAME], Swedish [dɑ:m]

****Cognate Set "1431":** Hindi [əʊrət̪], Urdu [ˈʔɔ.ɾət̪], Kurdish [afret]

- - 1. Basic Concept: "woods"

**Cognate Set "82":** Breton_List [KOAD], Welsh_N [COED]

**Cognate Set "342":** Danish [sg̥ɒw], Faroese [SKOG(V)UR], Icelandic_ST [ˈskouːʏr], Norwegian [SKOG], Swedish [skuːg]

**Cognate Set "3016":** Portuguese_ST [fluˈɾɛʃtɐ], Provencal [FOUREST]

**Cognate Set "252":** Byelorussian [LES], Czech [lɛs], Polish [las], Russian [lʲes], Slovak [LES], Ukrainian [LIS]

**Cognate Set "2226":** Provencal [SEUVO], Portuguese_ST [SELVA]

**Cognate Set "197":** Latvian [mežs], Lithuanian_ST [MISKAS]

***Cognate Set "2436":** Dutch_List [bɔs], Frisian [bosk], Catalan [bɔsk], French [bwa], Italian [ˈbɔsko], Portuguese_ST [BOSQUE], Sardinian_C [BOSKU], Spanish [ˈboske]

****Cognate Set "637":** Hindi [JENGEL], Urdu [ʤə̃ɡəl], Pashto [DZANGAL], Persian [dʒænɡæl]

- - 1. Basic Concept: "worm"

**Cognate Set "648":** Albanian_Standard [krimb], Breton_List [PRENV], Bulgarian [ˈt͡ʃɛrvɛj], Byelorussian [CARVJAK], Czech [tʃɛrf], Lithuanian_ST [KIRMELE], Pashto [KIRM], Persian [KERM], Russian [tʃervʲ], Serbocroatian [CRV], Slovak [CERV], Ukrainian [CERVˈJAK], Welsh_N [PRYF_GENWAIR]

**Cognate Set "2438":** French [vɛʀ], Italian [ˈvɛrme], Portuguese_ST [ˈvɛɾmɨ], Provencal [VERME], Rumanian_List [vierme], Sardinian_C [BREMI], Danish [oɐ̯m], Dutch_List [wɔrm], English [wɜ:m], Faroese [ORMUR], Frisian [vjɪrm], German [vʊrm]

**Cognate Set "2232":** Portuguese_ST [GUSANO], Spanish [guˈsano]

**Cognate Set "2230":** Faroese [ˈmakːʊr], Icelandic_ST [ˈmaθkʏr], Norwegian [MARK], Swedish [larv], Swedish [mask]

- - 1. Basic Concept: "year"

**Cognate Set "83":** Breton_List [BLOAZ], Irish_A [BLIADHAIN], Welsh_N [BLWYDDYN]

**Cognate Set "3017":** Catalan [aɲ], French [ɑ̃], Italian [ˈanno], Portuguese_ST [ˈɐnu], Provencal [AN], Rumanian_List [an], Sardinian_C [ANNU], Spanish [ˈaɲo]

**Cognate Set "299":** Ukrainian [RIK], Czech [rok], Polish [rɔk], Slovak [ROK]

**Cognate Set "2444":** Danish [ɒ], Dutch_List [jar], English [jɪə], Faroese [ɔar], Frisian [(j)i.ər], German [ja:ɐ̯], Icelandic_ST [auːr], Norwegian [AR], Swedish [o:r]

****Cognate Set "2817":** Persian [SAL], Hindi [SAL], Urdu [sɑl]

****Cognate Set "198":** Bulgarian [ɡoˈdinɐ], Byelorussian [HOD], Russian [god], Serbocroatian [GODINA], Latvian [gads]

- - 1. Basic Concept: "yellow"

**Cognate Set "84":** Breton_List [MELEN], Welsh_N [MELYN]

**Cognate Set "668":** Kurdish [zer], Pashto [ZER], Persian [ZARD], Danish [gul], Dutch_List [ɣel], English [ˈjɛləʊ], Faroese [GULUR], Frisian [ge:l], German [gɛlp], Icelandic_ST [ˈkʏːlʏr], Norwegian [ɡʉɽ], Swedish [ɡʉːl], Bulgarian [ʒɤ̞lt], Byelorussian [ZOWTY], Czech [ʒlʊtiː], Latvian [dzęl_̂-tęns], Lithuanian_ST [GELTONAS], Polish [ˈʒuwtɨ], Russian [ˈʒoltɨj], Serbocroatian [ZUT], Slovak [ZLTY], Ukrainian [ZOVTYJ]

**Cognate Set "578":** Hindi [pi:la], Urdu [pilɑ]

**Cognate Set "409":** French [ʒon], Italian [ˈdʒallo], Provencal [JAUNE], Rumanian_List [ˈɡalben]

**Cognate Set "3018":** Portuguese_ST [ɐmɐˈɾɛlu], Spanish [amaˈɾiʎo]

**Cognate Set "281":** Catalan [gɾɔk], Sardinian_C [GROGU]

- - 1. Basic Concept: "you"

**Cognate Set "386":** Faroese [TIT], Icelandic_ST [THIO]

**Cognate Set "2898":** Hindi [AP_(FORMAL)], Hindi [tʊm], Urdu [t̪ʊm]

**Cognate Set "2440":** Albanian_Standard [ju], Breton_List [CˈHOUI], Bulgarian [ˈviɛ], Byelorussian [VY], Catalan [buz’altrəs], Czech [vɪ], Danish [i], Digor_Ossetic [sumaχ], English [ji:], French [vu], Frisian [ji], German [i:ɐ̯], Irish_A [SIBH], Italian [ˈvoi], Latvian [jũs], Lithuanian_ST [JUS], Norwegian [DERE], Pashto [TASI], Persian [ʃomɒː], Polish [vɨ], Portuguese_ST [ˈvɔʃ], Provencal [VOUS], Rumanian_List [voj], Russian [vɨ], Sardinian_C [BOSATRUS], Serbocroatian [VI], Slovak [VY], Slovenian [JE], Spanish [boˈsotɾos], Swedish [niː], Welsh_N [xwi]
