# Supplementary material for: Networks of lexical borrowing and lateral gene transfer in language and genome evolution
Source: Bioessays. 2013 Dec 27;36(2):141–50. doi: 10.1002/bies.201300096 (PMC3910147; doi:10.1002/bies.201300096)
Supplement: Supplementary file 2 [file bies0036-0141-sd2.docx]

**Supplemental Material II: Comparing the findings of the MLN approach with known borrowings in English**

**A) Notes on the Tables**

The following table compares the results of the MLN method with known borrowings in English. For the borrowing assignments, we follow the data provided in Donohue et al. (2012, Supplemental material). There may be cases where scholars disagree, but in order to keep the comparison simple, we prefer to have but one source, instead of embarking on all known issues on the borrowing status of English basic words.

From the table, we excluded the following 8 words, since they are not cognate with any other word in the sample and hence cannot be shown to be borrowed by the MLN approach: *because*, *big*, *cut*, *dig*, *dirty*, *hit*, *pull*, and *sky*. We also exclude 5 words that have reflexes in all Germanic languages and can thus also not be identified as borrowings by our method, these are: *egg*, *fruit*, *give*, *root*, *stick*.

In the following, we list the words, the status, and the MLN-inference. The status-field lists the word's *status* with an abbreviation for the donor language, or the deepest etymology. The *MLN-inference* is either an “irregular pattern” or a “regular pattern”. The *note* field contains further information. Rows whose background is not shaded are generally correctly identified. Those cases where we are quite sure that the method detected an error or an undetected borrowing in the data, are shaded in yellow. Cases of probably erroneous inferences (false positives, false negatives) are shaded in gray.

**B) Known borrowings in English compared to inferences by the MLN approach**

| **Word** | **Status** | **MLN-Inference** | **Note** |
| --- | --- | --- | --- |
| *animal* | loan F | irregular pattern | Correctly identified irregular pattern. |
| *bark* | loan ON | irregular pattern | Correctly identified irregular pattern. |
| *count* | loan F | irregular pattern | Correctly identified irregular pattern. |
| *husband* | loan ON | irregular pattern | Correctly identified irregular pattern. |
| *lake* | loan F | irregular pattern | Correctly identified irregular pattern. |
| *leg* | loan ON | irregular pattern | Correctly identified irregular pattern. |
| *mountain* | loan F | irregular pattern | Correctly identified irregular pattern. |
| *person* | loan F | irregular pattern | Correctly identified irregular pattern. |
| *push* | loan F | irregular pattern | Correctly identified irregular pattern. |
| *river* | loan F | irregular pattern | Correctly identified irregular pattern. |
| *round* | loan L | irregular pattern | Correctly identified irregular pattern. |
| *rub* | loan LG | irregular pattern | Correctly identified irregularity due to Welsh *RHWBIO*, not due to the pattern of the English word itself. |
| *skin* | loan ON | irregular pattern | Correctly identified irregular pattern. |
| *they* | loan ON | irregular pattern | Correctly identified irregular pattern. |
| *turn* | loan F | irregular pattern | Correctly identified irregular pattern. |
| *vomit* | loan F | irregular pattern | Correctly identified irregular pattern. |
| *wing* | loan ON | irregular pattern | Correctly identified irregular pattern. |
| *die* | loan ON | regular pattern | Irregular pattern not identified. |
| *split* | loan LG | regular pattern | Irregular pattern not identified. |

**C) Further cognate sets inferred to be patchy by the MLN approach**

| **Word** | **Status** | **MLN-Inference** | **Note** |
| --- | --- | --- | --- |
| *at* | PIE | irregular pattern | Clear case of parallel semantic development erroneously found to be suggestive of borrowing. |
| *back* | PG | irregular pattern | Parallel semantic development (only reflexes in Scandinavian languages), or so far unrecognised borrowing in English. |
| *belly* | PG | irregular pattern | Reflexes in Irish *BOLG* and Welsh *BOL*. The fact that there are only reflexes in these neighbouring languages suggests an undetected borrowing. |
| *dull* | PG | irregular pattern | Proposed reflex in Breton_List *DALLA* could be a borrowing or a miscoding. Anyway it is strange, since the English word is normally not reconstructed deeper than to the PG level. |
| *few* | PG | irregular pattern | Parallel semantic development (only reflexes in Scandinavian languages inside Germanic), or so far unrecognised borrowing in English. |
| *know* | PIE | irregular pattern | Clear case of parallel semantic development erroneously found to be suggestive of borrowing. |
| *leaf* | PIE | irregular pattern | Clear case of parallel semantic development erroneously found to be suggestive of borrowing. |
| *many* | PG | irregular pattern | Parallel semantic development (only reflexes in Scandinavian languages inside Germanic), or so far unrecognised borrowing in English. |
| *narrow* | PG | irregular pattern | Proposed reflex in Armenian_Mod *nɛʁ* could be a borrowing or a miscoding. Anyway it is strange, since the English word is normally not reconstructed deeper than to the PG level. |
| *small* | PIE | irregular pattern | Clear case of parallel semantic development erroneously found to be suggestive of borrowing. |
| *smoke* | PIE | irregular pattern | Reflexes in Breton_List *MOGED* and Welsh_N *MWG*. The fact that there are only reflexes in these neighbouring languages suggests an undetected borrowing. |
| *snake* | PG | irregular pattern | Parallel semantic development (only reflexes in Scandinavian languages), or so far unrecognised borrowing in English. |
| *tree* | PIE | irregular pattern | Parallel semantic development (only reflexes in Scandinavian languages inside Germanic), or so far unrecognised borrowing in English. |
| *with* | PIE | irregular pattern | Parallel semantic development (only reflexes in Scandinavian languages inside Germanic), or so far unrecognised borrowing in English. |
| *dust* | reflex of donor | irregular pattern | Correctly identified irregularity due to Irish *DUSTA.*. |
| *rope* | reflex of donor | irregular pattern | Correctly identified irregularity due to Irish *ROPA.* |
| *short* | reflex of donor | irregular pattern | Correctly identified irregularity due to Albanian *i shkurtër.* |
